# *Larrea tridentata*: A novel source for anti-parasitic agents active against *Entamoeba histolytica*, *Giardia lamblia* and *Naegleria fowleri*

**DOI:** 10.1371/journal.pntd.0005832

**Published:** 2017-08-09

**Authors:** Bharat Bashyal, Linfeng Li, Trpta Bains, Anjan Debnath, Daniel V. LaBarbera

**Affiliations:** 1 Department of Pharmaceutical Sciences, Skaggs School of Pharmacy and Pharmaceutical Sciences, University of Colorado Anschutz Medical Campus, Aurora, Colorado, United States of America; 2 Center for Discovery and Innovation in Parasitic Diseases, Skaggs School of Pharmacy and Pharmaceutical Sciences, University of California San Diego, La Jolla, California, United States of America; University of Tokyo, JAPAN

## Abstract

Protozoan parasites infect and kill millions of people worldwide every year, particularly in developing countries where access to clean fresh water is limited. Among the most common are intestinal parasites, including *Giardia lamblia* and *Entamoeba histolytica*. These parasites wreak havoc on the epithelium lining the small intestines (*G*. *lamblia*) and colon (*E*. *histolytica*) causing giardiasis and amebiasis, respectively. In addition, there are less common but far more deadly pathogens such as *Naegleria fowleri* that thrive in warm waters and infect the central nervous systems of their victims via the nasal passages. Despite their prevalence and associated high mortality rates, there remains an unmet need to identify more effective therapeutics for people infected with these opportunistic parasites. To address this unmet need, we have surveyed plants and traditional herbal medicines known throughout the world to identify novel antiparasitic agents with activity against *G*. *lamblia*, *E*. *histolytica*, and *N*. *fowleri*. Herein, we report *Larrea tridentata*, known as creosote bush, as a novel source for secondary metabolites that display antiparasitic activity against all three pathogens. This report also characterizes the lignan compound classes, nordihydroguairetic acid and demethoxyisoguaiacin, as novel antiparasitic lead agents to further develop more effective drug therapy options for millions of people worldwide.

## Author summary

*Entamoeba histolytica*, *Giardia lamblia*, and *Naegleria fowleri* pathogens are widespread throughout the world infecting and killing hundreds of thousands of people every year. They are also listed as category B bioterrorism agents by the NIH and the CDC. However, there is a serious unmet need to develop more effective therapies to treat these deadly pathogens. Herein we describe that lignans isolated from the creosote bush, common to the southwestern U.S.A. and throughout Mexico, display relatively potent antiparasitic activity against *E*. *histolytica*, *G*. *lamblia*, and *N*. *fowleri*.

## Introduction

**Fig 1:**
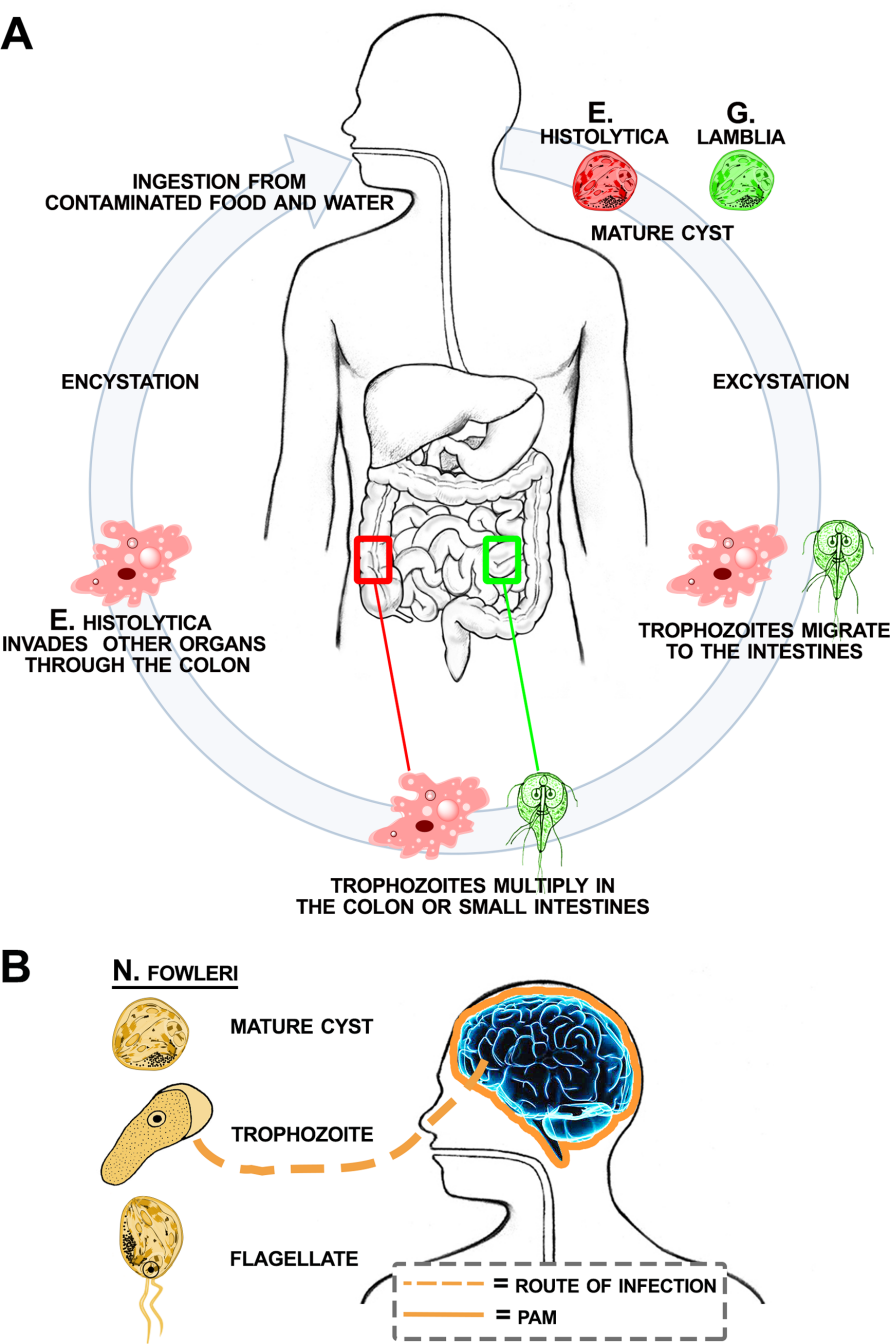
(A) The infection cycle of *G*. *lamblia* (green) and *E*. *histolytica* (red). The protozoan cysts are ingested through contaminated food or water. Viable cysts undergo excystation after passing through the acidic environment of the stomach to release the trophozoites that attach or migrate to the intestines. Trophozoites will either remain in the lumen of the small intestines (*G*. *lamblia*) or invade through the colon mucosa (*E*. *histolytica*). The parasites also undergo encystation and excretion from the host body to further infect human populations through contaminated food and water supplies. (B) *N*. *fowleri* thrives in warm fresh water and hot springs. Under these conditions, *N*. *fowleri* infects humans via the nasal sinuses invading the brain and its infection causes swelling of the brain, termed primary amoebic meningoencephalitis (PAM).

Intestinal protozoan parasite infections, through contaminated water and food supplies, are global health problems affecting hundreds of millions of people annually. The two most common intestinal parasites are *Giardia lamblia* and *Entamoeba histolytica*, which can lead to giardiasis or invasive amebiasis, respectively. *G*. *lamblia and E*. *histolytica* have simple infection life cycles that begin with ingesting viable cysts, excystation, followed by trophozoite multiplication in the small intestine or trophozoite migration and invasion in the colon (Fig 1A) [1–3]. Annually, giardiasis, has an estimated worldwide prevalence of 200 million cases [4], and according to the World Health Organization (WHO) giardia infections contribute substantially to the 846,000 deaths annually from diarrheal disease [5, 6]. Once *G*. *lamblia* has excysted in the small intestines, trophozoites attach to epithelial cells and elicit aberrant signaling events that disrupt organ function including the induction of programed cell death or apoptosis [3]. Although less prevalent than *G*. *lamblia*, *E*. *histolytica* infections lead to 50 million cases of invasive disease and up to 100,000 deaths, annually [7]. Invasive amebiasis is characterized by profound intestinal tissue damage and ulceration [8]. Recently, Ralston and colleagues determined trogocytosis as the mechanism by which *E histolytica* feeds on its host. The term ‘trogocytosis’ was taken from the Greek word trogo which means to nibble [8, 9]. The amebae damage and consume the intestinal mucosa epithelium by nibbling away at epithelial cell membranes, triggering cell death. Interestingly, Ralston *et al*. concluded that amebae feed on bacteria in the gut for nutrition but that host cell ingestion is done by the amebae to create a more spacious environment [8].

Free-living ameba *Naegleria fowleri* has been described as the cause of primary amebic meningoencephalitis (PAM) in more than 16 countries [10]. Until 2012, about 310 cases have been reported globally with a fatality rate of more than 95% [11]. According to the Centers for Disease Control and Prevention (CDC), 138 cases of PAM have been reported in the U.S.A. from 1962–2015 with a 98% mortality rate. PAM results from water containing *N*. *fowleri* entering the nasal cavity followed by migration of the amebae to the brain (Fig 1B) [12–17]. Within the brain, *N*. *fowleri* causes extensive inflammation, hemorrhage, and necrosis. The time from initial exposure to onset of illness is usually 5–7 days but may be as early as 24 h, leading to death in 3 to 7 days [18].

Treatment for giardiasis and invasive amebiasis is largely limited to the nitroimidazole drug class (e.g. metronidazole) [19]. Metronidazole, is the primary drug of choice, which requires a relatively long treatment time and high dosage to eradicate intestinal parasite infections [20]. Moreover, metronidazole is both mutagenic and carcinogenic and its use presents other significant adverse effects [21, 22]. In addition, *G*. *lamblia* and *E*. *histolytica* drug resistance and treatment failures remain an increasing problem [23–26]. Amphotericin B remains a cornerstone of therapy for PAM but is not FDA-approved for this indication and has had limited success despite its worldwide use [27]. Treatment with amphotericin B requires a high dosage and its use is frequently associated with renal toxicity and anemia, among other adverse effects [27]. Recently, an investigational drug, miltefosine, clinically used to treat leishmaniasis, has shown some promise in combination with other drugs as a treatment for PAM [28]. The CDC, through an established protocol with the FDA, is now directly providing miltefosine to the clinicians as a treatment option for PAM. However, it is still not FDA approved and has limited availability in the U.S.A. Furthermore, *G*. *lamblia*, *E*. *histolytica* and *N*. *fowleri* are listed by the United States National Institutes of Health and the Centers for Disease Control as a category B biodefense/bioterrorism pathogens due to their low infectious dose and potential for dissemination through compromised food and water supplies. Given the prevalence and mortality caused by these protozoan pathogens, compounded by their potential bioterrorism threat, more effective antiparasitic agents is a critical unmet need to treat the current pandemic and avert future outbreaks and deaths.

**Fig 2:**
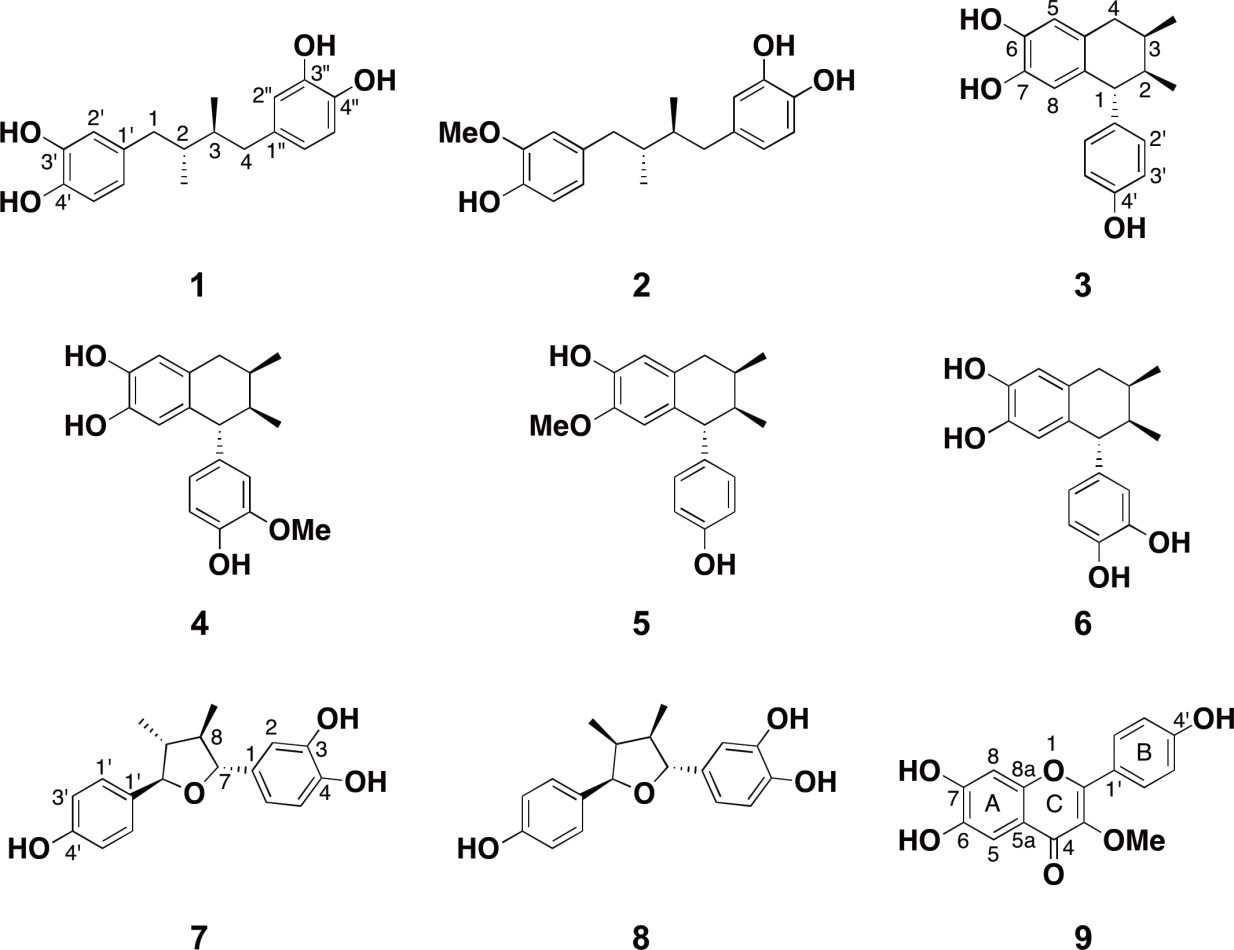
Structures of compound 1–9.

Natural products have played an important role throughout history in the treatment of human disease through traditional medicines and as a source for effective pharmaceutical development [29, 30]. In particular, plants have been a vast source of secondary metabolites that display potent antiparasitic activity, including protozoan parasites [30–33]. For example, *G*. *lamblia* and *E*. *histolytica* are endemic to Mexico and infections are prevalent [34, 35]. Moreover, nitroimidazole drugs display limited efficacy in the Mexican population [36]. Therefore, scientists have turned to native plants used as Mexican traditional medicines for intestinal diseases in the search for novel more effective antiparasitic agents [37, 38]. Similarly, using our established assays [39, 40], we have surveyed plants used as traditional medicines from around the world and that are common to the southwestern United States and throughout Mexico. Herein, we report the discovery of *Larrea tridentata*, commonly known as creosote bush or chaparral, as a novel source for antiparasitic secondary metabolites [41]. Though the extract of *L*. *tridentata* earlier showed antiparasitic activity against *Trypanosoma brucei rhodesiense*, *T*. *cruzi*, *Leishmania donovani* and *Plasmodium falciparum* [42], this is the first report to show their activity against a free-living amoeba *N*. *fowleri* and against diarrhea causing parasites *E*. *histolytica* and *G*. *lamblia*. We have identified seven known compounds **1–7** (Fig 2) with **1–6** displaying antiparasitic activity against *E*. *histolytica*, *G*. *lamblia*, and *N*. *fowleri*. Compounds **1** and **2** showed better activity against *N*. *fowleri* than the current drug miltefosine. In addition, we have identified two secondary metabolites, compounds **8** and **9** (Fig 2), that we isolated from the same active fractions as **1–7** that appeared to have novel structures. Compound **9** displayed modest antiparasitic activity against *G*. *lamblia* and *N*. *fowleri*. An examination of the literature indicated that **8** and **9** structures have been reported [43, 44]. Interestingly, compound **8** has not previously been isolated or structurally characterized from the creosote plant, rather, Cho and colleagues used Larreatricin 3’-hydroxylase enzyme purified from creosote and the known secondary metabolite from creosote, larreatricin, to enzymatically prepare **8**, albeit in very low yield [44]. However, the structure of **9** was dubiously deduced from *Graziela mollisima* as an impure mixture with insufficient analytical data to accurately characterize the structure [43]. Therefore, this is the first report to unambiguously characterize the novel secondary metabolites **8** and **9** from *L*. *tridentata*. Since compounds **1** and **2** were found more active against *N*. *fowleri* than miltefosine, we selected these two compounds to investigate their ability to inhibit *N*. *fowleri* cysteine protease, an enzyme shown to play an important role in host tissue invasion by *N*. *fowleri* [45].

## Methods

### General experimental procedures

^1^H, ^13^C and 2D NMR spectra were recorded on a Bruker Avance III spectrometer (400 MHz for ^1^H NMR and 100 MHz ^13^C NMR). Chemical shifts are recorded in ppm (δ) using residual solvent signal as internal reference, and coupling constants (*J*) are reported in Hz. The following splitting abbreviations were used for NMR signals: s = singlet, d = doublet, t = triplet, q = quartet, m = multiplet, br = broad. High-resolution mass spectra (HRMS) were recorded on a Bruker Q-TOF-2 Micromass spectrometer equipped with lock spray, using ESI with methanol as the carrier solvent. Accurate mass measurements were performed using leucine enkephalin as a lock mass and the data were processed using MassLynx 4.1. Exact *m/z* values are reported in Daltons. Optical rotations were measured in CH_3_OH on a JASCO P1010 polarimeter at 589 nm (Na D-line) with a path length of 1 dm and are reported with implied units of 10^−1^ deg cm^2^ g^-1^. Concentrations (c) are given in g/100 mL. UV was measured in CH_3_OH on an Agilent 8453 UV-Visible Spectrophotometer. Analytical and preparative HPLC were performed on a Shimadzu Prominence HPLC system equipped with LC-6AD pumps, an autosampler (SIL-20AC) and manual injection port (Rheodyne, 3725i), a column oven (CTO-20A, temperature set at 27°C), a photo diode array detector (SPD-M20A, using a Deuterium lamp and a tungsten lamp as light sources) and a system controller (CBM-20A). A Phenomenex Kinetex C18 reversed phase column (5 μm, 100 Å, 250 ✕ 4.6 mm) fitted with a guard cartridge, with a flow rate of 0.7 mL/min was used for analytical chromatography, and a Phenomenex Kinetex C18 reversed phase column (5 μm, 100 Å, 250 ✕ 21.1 mm) fitted with a guard cartridge with a flow rate of 5.0 mL/min was used for preparative chromatography. The HPLC data were processed using LabSolutions Lite software (version 5.22).

### Extraction and isolation

The dried powdered material (11.0 g) of *L*. *tridentata* (Mountain Rose Herbs) was extracted with methanol at room temperature for 72 h. After filtration through Celite, the methanol extract was concentrated under reduced pressure to give a crude residue (2.55 g). The extract residue (2.53 g) was treated with water (150 mL) and partitioned against hexane (150 mL × 3), ethyl acetate (150 mL × 3) and n-butanol (150 mL × 2) successively to yield a hexane fraction (128.6 mg), an ethyl acetate fraction (1.5 g), a n-butanol fraction (411.7 mg), and a water fraction (504.2 mg), respectively. The parasite active ethyl acetate fraction (808.5 mg) was then chromatographed on a Sephadex LH-20 column eluted with 20% hexane in CH_2_Cl_2_ (200 mL), 60% CH_2_Cl_2_ in acetone (400 mL), 20% CH_2_Cl_2_ in acetone (200 mL), 20% CH_2_Cl_2_ in methanol (200 mL), and 100% methanol (200 mL). Ten fractions were collected: fractions A (12.9 mg) and B (13.4 mg) from 20% hexane in CH_2_Cl_2_; fractions C (28.6 mg), D (386.1 mg), E (181.9 mg), and F (81.6 mg) from 60% CH_2_Cl_2_ in acetone; fractions G (48.7 mg) and H (35.9 mg) from 20% CH_2_Cl_2_ in acetone; fraction I (52.6 mg) from 20% CH_2_Cl_2_ in methanol and fraction J (2.1 mg) from 100% methanol. Fraction E (138.9 mg) was chromatographed on reverse phase preparative HPLC and eluted with gradient 20–100% acetonitrile in water for 40 min to yield **1** (11.7 mg) and **3** (43.3 mg) as yellowish resinous solid along with sub-fraction E1 (11.0 mg). Sub-fraction E1 was re-chromatographed under similar HPLC conditions to afford **2** (4.0 mg), **7** (3.3 mg), and **8** (1.8 mg). Fraction D (386.1 mg) was chromatographed on silica gel column (13.0 g) eluted with increasing amounts of methanol in CH_2_Cl_2_ to afford seven fractions, D1 (0.6 mg), D2 (181.1 mg), D3 (73.5 mg), D4 (65.6 mg), D5 (5.6 mg), D6 (5.5 mg), D7 (7.5 mg). Fraction D2 (133.0 mg) was chromatographed on preparative HPLC and eluted with isocratic 50% acetonitrile in water to yield **2** (29.7 mg), **4** (14.6 mg), **5** (2.0 mg) and **6** (4.5 mg) as yellow resinous solids.

### Characterization of compounds 8 and 9

*(7R*, *7’R)-7*, *7’-bis(4’*, *3*, *4-trihydroxyphenyl)-(8R*, *8’S)-8*, *8’-dimethyltetrahydrofuran (****8****)*: colorless oil; [α]_D_^25^–88.1 (*c* 0.16, CH_3_OH); UV (MeOH) λ_max_ (log ε) 211 (3.44); 236 (2.54), 282 (1.64); ^1^H and ^13^C NMR data, see Table 1; HRESIMS m/z 301.1506 [M + H]^+^ (calcd for C_18_H_21_O_4,_ 301.1439)

**Table 1:** ^1^H (400 MHz) and ^13^C NMR (100 MHz) data for 8 compared to the crude reported ^1^H NMR data ((CD_3_)_2_CO) ^a^.

Position	Compound 8		Reported^a^	
	δ_H_	δ_C_	δ_H_	δ_C_
1	—	136.5	—	136.1
2	6.91 br s	114.0	6.93 d (1.8)	113.7
3	—	146.0	—	145.5
4	—	145.1	—	144.8
5	6.81 d (7.2)	115.7	6.80 d (8.3)	115.4
6	6.72 br d (7.2)	118.5	6.73 dd (8.3, 1.8)	118.1
7	4.54 d (9.4)	86.2	4.55 d (9.5)	85.8
8	2.44 m	44.0	2.40 m	48.1
9	0.97 d (6.6)	12.2	0.99 d (6.7)	11.8
1’	—	132.8	—	132.4
2’, 6’	7.17 d (8.1)	128.0	7.19 d (8.4)	127.6
3’, 5’	6.81 d (7.8)	115.5	6.82 (8.4)	115.1
4’	—	157.0	—	156.6
7’	5.38 d (4.2)	85.2	5.40 d (5.4)	84.8
8’	2.38 m	48.4	2.44 m	43.7
9’	0.57 d (7.1)	9.7	0.58 d (7.0)	9.4
OH × 3	7.94	—	7.96	—

^a^ The data reported for the ± racemic mixture [44].

*3-Methoxy-6*, *7*, *4’-trihydroxyflavonol (****9****)*: Yellow solid; UV (MeOH) λ_max_ (log ε) 211 (5.06), 266 (4.90), 348 (4.86); ^1^H and ^13^C NMR data, see Table 2; HRESIMS m/z 301. 0690 [M + H]^+^ (calcd for C_16_H_13_O_6,_ 301.0712).

**Table 2:** ^1^H (400 MHz) and ^13^C NMR (100 MHz) data for 9 (CDCl_3_ + CD_3_OD) compared to the crude reported ^1^H NMR data (CD_3_OD)^a^.

Position	δ_H_	δ_C_	Reported δ_H_^a^
1	—	—	—
2	—	156.5	—
3	—	138.4	—
4	—	178.8	—
5	6.20	98.9	6.51 s
6	—	161.5	—
7	—	163.9	—
8	6.35 s	94.1	6.48 s
5a	—	105.2	—
8a	—	157.0	—
1’	—	121.7	—
2’, 6’	7.93 d (8.6)	130.3	7.76 d (9.0)
3’, 5’	6.88 d (8.6)	115.6	6.85 d (9.0)
4’	—	159.7	—
OMe	3.74 s	60.1	3.76

^a^The data from the reported crude 2:1 mixture [43].

### Parasite assay

Trophozoites of *E*. *histolytica* HM1: IMSS and *G*. *lamblia* WB strains were axenically maintained in TYI-S-33 medium supplemented with penicillin (100 U/ml), streptomycin (100 μg/ml) [46, 47]. Trophozoites of *N*. *fowleri* strain KUL were axenically cultured in Nelson’s medium supplemented with 10% FBS at 37°C [45]. All experiments were performed using trophozoites harvested during the logarithmic phase of growth. Four solvent partitioned fractions of an aqueous methanolic extract of *L*. *tridentata* and compounds **1**–**9** were screened for activity against *E*. *histolytica*, *G*. *lamblia*, *and N*. *fowleri*. For primary screening, the positive control for *E*. *histolytica* and *G*. *lamblia* was 5 μg/mL of metronidazole (Sigma-Aldrich) and 46 μg/mL of amphotericin B for *N*. *fowleri* (Sigma-Aldrich). Test samples were diluted to 10 mg/mL of extracts, HPLC fractions, and pure compounds in DMSO. Finally, 0.5 μL of diluted sample was transferred to white, solid bottom tissue culture 96-well plates (E&K Scientific) followed by addition of 99.5 μL trophozoites (5,000 *E*. *histolytica* and *G*. *lamblia*, and 10,000 *N*. *fowleri*) in TYI-S-33 medium or Nelson’s medium. The final concentration for test compounds was 50 μg/mL and 0.5% DMSO, which was the negative control and compound vehicle that we have shown has no effect the growth rate of trophozoites [39, 40, 48]. Assay plates were incubated for 48 h at 37°C. *E*. *histolytica* and *G*. *lamblia* plates were kept in the GasPak EZ Anaerobe Gas Generating Pouch System (VWR) to maintain anaerobic condition throughout the incubation period. Screening was performed in duplicate using the CellTiter-Glo assay (Promega) and luminescence was measured using an EnVision plate reader (PerkinElmer) [40, 48].

The antiparasitic activity of **1–6** and **9** were confirmed by EC_50_ dose response experiments, using the CellTiter-Glo assay, conducted in triplicate over a concentration range from 5-to-700 μM against trophozoites (Table 3). Miltefosine and metronidazole, current drugs for the treatment of PAM and amebiasis and giardiasis were also tested in triplicate as positive controls for EC_50_ determination (Table 3). Dose response curves including standard deviation (SD) calculation were processed using GraphPad Prism software 5.0. Percent inhibition relative to maximum and minimum reference signal controls was calculated using the formula: % Inhibition = [(mean of Maximum Signal Reference Control—Experimental Value)/(mean of Maximum Signal Reference Control—mean of Minimum Signal Reference Control)] × 100.

**Table 3:** EC_50_^a^ antiparasitic activity of *L*. *tridentata* lignans 1–8 and flavonol 9.

Compounds	*E*. *histolytica* EC_50_ (pEC_50_ ± SE) (μM)	*G*. *lamblia* EC_50_ (pEC_50_ ± SE) (μM)	*N*. *fowleri* EC_50_ (pEC_50_ ± SE) (μM)
**1**	103 (4 ± 0.03)	36 (4.4 ± 0.02)	37 (4.4 ± 0.03)
**2**	171 (3.8 ± 0.07)	38 (4.4 ± 0.02)	38 (4.4 ± 0.02)
**3**	94 (4 ± 0.03)	49 (4.3 ± 0.03)	73 (4.1 ± 0.04)
**4**	83 (4.1 ± 0.04)	74 (4.1 ± 0.02)	75 (4.1 ± 0.02)
**5**	236 (3.6 ± 0.03)	188 (3.7 ± 0.02)	155 (3.8 ± 0.01)
**6**	146 (3.8 ± 0.02)	96 (4 ± 0.07)	150 (3.8 ± 0.02)
**7**	—	—	—
**8**	—	—	—
**9**	—	153 (3.8 ± 0.02)	235 (3.6 ± 0.02)
**Metronidazole**	5 (5.3 ± 0.03)	6.4 (5.2 ± 0.02)	—
**Miltefosine**	—	—	54.5 (4.3 ± 0.01)

^a^EC_50_ minimum n = 3

(—) represents inactivity

### HUVEC cell cytotoxicity

The HUVEC-TERT2 cell line was purchased from Evercyte GmbH (Vienna, Austria) and cultured and maintained in endothelial cell basal medium (Lonza) as described previously [49, 50]. Briefly, cells were seeded into a white 384-well solid bottom plate (Nunc, ThermoFisher) at a density of 1000 cells/well in 39 μL of media using a Janus liquid handler (PerkinElmer). Serial dilutions using 1 μL of compound **1** and **2** at varying concentrations were dispensed into each well in triplicate. After 48 h incubation, 40 μL of CellTiter-Glo reagent (Promega) was added into each well. The contents were mixed for 2 min on a microplate shaker to induce cell lysis and further incubated at room temperature for 10 min to stabilize the luminescent signal. Luminescence was measured with an EnVision plate reader (PerkinElmer) and %inhibition calculations were performed using the following formula for single-point normalization: %Inhibition = (1-Raw Sample Value/Mean of DMSO Signal Reference Value) × 100. Dose response curves including EC_50_ calculations were processed using GraphPad Prism software.

### Cysteine protease activity assay

To prepare the cell lysate, *N*. *fowleri* trophozoites were removed from the culture flask surface by incubating in an ice bath for 10 min, centrifuged at 300 g for 10 min, and washed twice with PBS (pH 7.2). The cells were disrupted by four cycles of freeze thawing in PBS [51]. Protein concentration was quantified by the method of Bradford (Bio-Rad). The activity of the cysteine protease present in the crude extract after incubating in presence and absence of different concentrations of compounds **1** and **2** was assayed by the liberation of the fluorescent leaving group, 7-amino-4-methyl coumarin (AMC), from the peptide substrate Z–Phe–Arg–AMC (40 μM) (where Z is benzyloxycarbonyl, R&D Systems) [45]. The assay was performed at 25°C in an automated microtiter plate spectrofluorometer (EnVision, PerkinElmer) with excitation wavelength at 355 nm and emission wavelength at 460 nm [52]. Enzyme samples were added to the reactivation buffer (10 mM Tris, 5 mM EDTA, 50 mM NaCl, pH 7.4, 10 mM DTT), and preincubated for 20 min at 37°C prior to the hydrolysis of substrate. The rate of substrate hydrolysis at ambient temperature was determined from the rate of increase of fluorescence, monitored on a continuously recording spectrofluorometer and measured as RFU/min/μg protein.

## Results

### Isolation and structure elucidation

An aqueous methanolic extract of the creosote plant was partitioned against hexane, ethyl acetate and n-butanol successively to obtain four solvent partitioned fractions. These fractions were tested for antiparasitic activity, the ethyl acetate fraction showed activity at 50 μg/mL and was selected for further study. It was fractionated on Sephadex LH-20 and the fractions were subjected to chromatographic separation by HPLC to yield **1–9** as pure compounds.

Compound **1** was obtained as a yellow resinous mass. The ^1^H, ^13^C, and HMQC NMR (acetone-d_6_) indicated 9 carbon resonances and corresponding proton signals, consisting of one methyl [δ_H_ 0.83 d (6.6)], four methines [δ_H_ 1.74 m], three aromatic signals displaying an ABC splitting pattern [δ_H_ 6.52 dd (7.9, 1.8); δ_H_ 6.69 d (1.8); and δ_H_ 6.73 d (7.9)], and one methylene [δ_H_ 2.21 dd (13.3, 9.2); δ_H_ 2.70 dd (13.3, 5.0)]. These data were identical with the known creosote secondary metabolite, nordihydroguairetic acid (NDGA) (Table S1 and Fig. S1-S3 in S1 Appendix) [53]. Next, we identified known compound **2** as 3’-*O*-methylnordihydroguairetic acid (3’-*O*-methyl-NDGA) [54]. Although similar in structure to **1**, compound **2** is non-symmetrical, which revealed the full 19 carbon resonances and corresponding proton signals as follows: two methyls [δ_H_ 0.82 d (6.6), 0.83 d (6.6)], eight methines (δ_H_ 1.74 m, 2H), six aromatics [δ_H_ 6.58 dd (8.0, 2.0), δ_H_ 6.61 d (1.9), δ_H_ 6.64 dd (8.0, 1.9), δ_H_ 6.67 d (2.0), δ_H_ 6.77 d (8.0), and δ_H_ 6.82 d (8.0)], and two methylenes [δ_H_ 2.25 dd (13.1, 9.3), δ_H_ 2.71 dd (13.3, 4.8), δ_H_ 2.25 dd (13.1, 9.4), δ_H_ 2.68 dd (13.3, 5.0)]. In addition, DEPT-135 and HMQC supported the presence of two methyls (δ_c_ 16.6, 16.4), eight methines of which two aliphatic (δ_c_ 39.3, 39.1) and six aromatic (δ_c_ 113.2, 115.4, 115.8, 116.9, 121.2, 122.3), two methylenes (δ_c_ 40.0, 39.2) and six quaternary aromatic (δ_c_ 134.1, 134.3, 143.8, 145.4, 145.7, 48.1) (Table S2 and Fig. S4-S8 in S1 Appendix).

We identified compound **3** as Nor-3’-demethoxyisoguaiacin and **4–6** as analogs of **3** that have a tetrahydronaphthalene ring system [54, 55]. The ^1^H NMR (CDCl_3_) displayed the following signals: two methyls [δ_H_ 0.88 d (6.9), 0.89 d (6.9)], nine methines including three aliphatic [δ_H_ 3.57 d (6.2), 1.89 m, 1.99 m], two aromatic singlets (δ_H_ 6.60 s, δ_H_ 6.29 s) resulting from an A_2_B_2_ tetra-substituted phenyl ring, four signals giving an A_2_B_2_ splitting pattern [δ_H_ 6.86 (2H, d 8.5), δ_H_ 6.69 (2H, d 8.5)] due to a 1,4-disubstituted phenyl, and one methylene [δ_H_ 2.83 dd (16.4, 5.5), δ_H_ 2.41 dd (16.4, 7.2)]. The ^13^C NMR (acetone-d_6_) displayed eighteen signals and HMQC supported the presence of two methyls (δ_c_ 16.1, 16.3), three methines (δ_c_ 50.8, 41.8, 30.1), one methylene (δ_c_ 35.7), one A_2_B_2_ substituted phenyl (δ_c_ 115.9 d, 117.7 d, 128.1 s, 130.7 s, 140.0 s, 144.4 s), and one 1,4-disubstituted phenyl [δ_c_ 115.7 (2C, d), 130.8 (2C, d), 139.3 s, 156.3 s] (Table S3, Fig. S9-S13 in S1 Appendix). **4–6** were easily dereplicated due to varying methoxy and phenol substituents. Specifically, compound **4** (Nor-isoguaicin) has a methoxy in the 3’-position, which was determined by the ABC proton splitting pattern [δ_H_ 6.79 d (8.0), δ_H_ 6.52 d (1.8), δ_H_ 6.50 dd (8.0, 1.8)] from the tri-substituted phenyl ring (Table S4 and Fig. S14-S18 in Appendix). Conversely, compounds **5** (3’-Demethoxyisoguaiacin) has a methoxy group in the 7 position of the tetra-substituted ring (Table S5 and Fig. S19-S22 in S1 Appendix) and **6** (6,3'-Di-*O*-demethylisoguaiacin) which contains a 3’,4’-dihydroxy phenyl moiety were determined by comparison with the reported chemical shifts (Table S6 and Fig. S23-S25 in S1 Appendix) [54, 56]. Finally, **7** was purified as a colorless oil and identified as 3-hydroxy-4-epi-larreatricin with ^1^H and ^13^C NMR matching the known literature structure (Table S7 and Fig. S26-S30 in S1 Appendix) [57].

**Fig 3:**
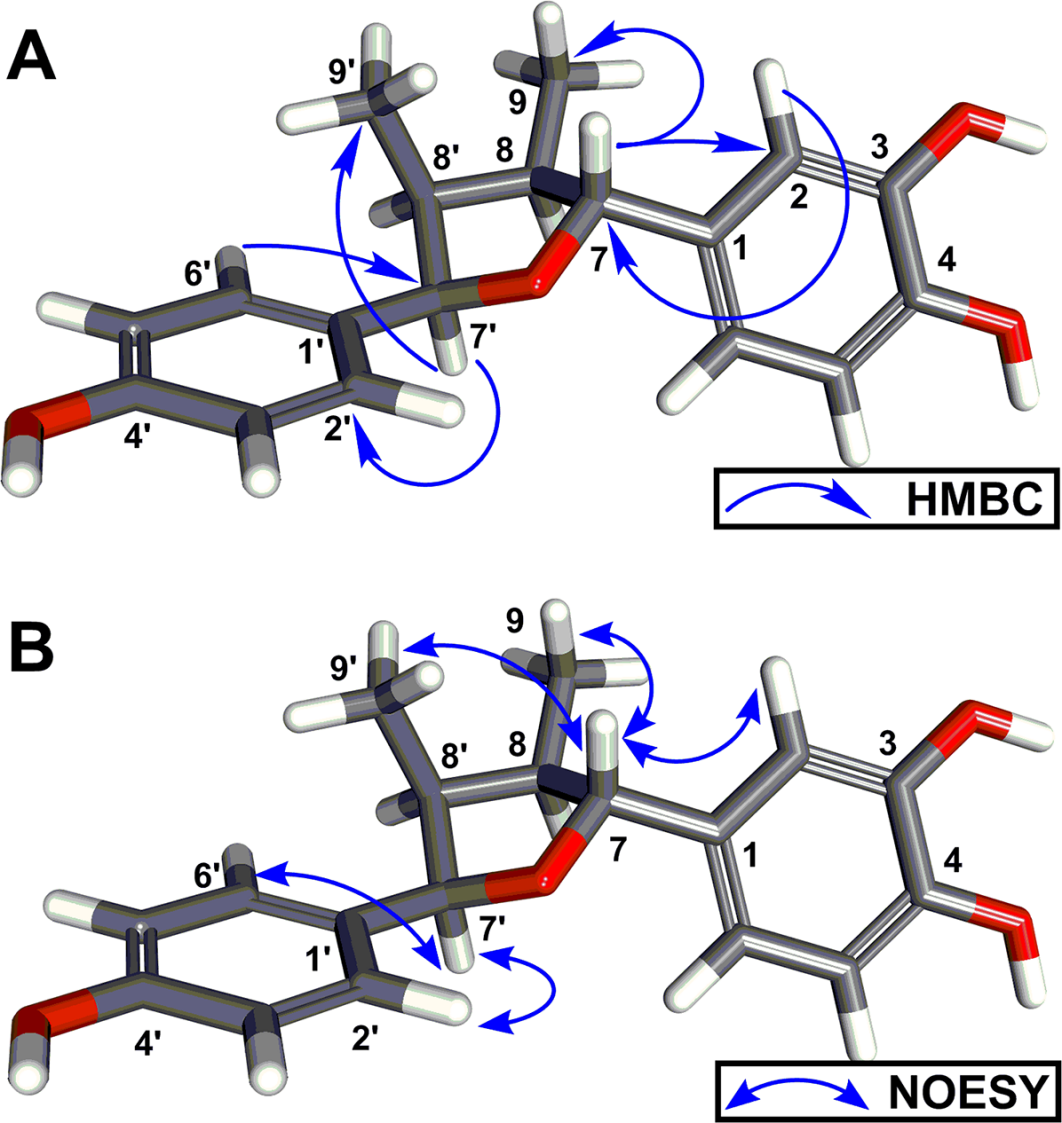
(A) Key HMBC correlations of compound **8.** (B) Key 1D gradient enhanced NOESY correlations of compound **8**. 3D energy minimized conformers were generated using BIOVIA Discovery Studio 2017 software.

During the purification of **1–7** we identified lignan **8** and flavanol **9**, however, these secondary metabolites have never been isolated from the creosote plant (**8**) or were not structurally well characterized (**9**). Therefore, we report herein the isolation and structure elucidation from the creosote plant. Compound **8**, was purified as a colorless oil and the molecular formula was deduced from the HRMS and ^13^C NMR as C_18_H_20_O_4_. The ^1^H NMR (Table 1) displayed signals attributable to two methyl groups [δ_H_ 0.97 d (6.6), δ_H_ 0.57 d (7.1)], and eleven methines, including: two oxygenated aliphatic protons [δ_H_ 5.38 d (4.2), δ_H_ 4.54 d (9.4)], two aliphatic protons [δ_H_ 2.38–2.44, m, 2H], four aromatic protons giving an A_2_B_2_ splitting pattern [δ_H_ 7.17 d (8.1), δ_H_ 6.81 d (7.8)], and three aromatic protons giving an ABC splitting pattern [δ_H_ 6.91 br s, δ_H_ 6.81 br dd (7.2), δ_H_ 6.72 d (7.2)]. The ^13^C NMR revealed the occurrence of eighteen carbons resonances, DEPT-90 in conjunction with HMQC supported the presence of seven aromatic methines, including A_2_B_2_ [δ_c_ 128.0 x 2 and δ_c_ 115.5 x 2] and ABC splitting patterns (δ_c_ 118.5, δ_c_ 115.7 and δ_c_ 114.0). Further, we observed two oxygenated [δ_c_ 86.2 and δ_c_ 85.2] and two non-oxygenated (δ_c_ 48.4 and δ_c_ 44.0) methines as well as two methyl functional groups (δ_c_ 12.2 and δ_c_ 9.7). The remaining five quaternary ^13^C NMR signals were indicative of aromatic chemical shifts (δ_c_ 157.0, 146.0, 145.1, 136.5 and 132.8). These NMR data were identical with the previously reported enzymatically synthesized (±) 3-hydroxy-larreatricin [44]. We observed HMBC correlations from aromatic H-2 (δ_H_ 6.91) of the tri-substituted phenyl ring to C-7 (δ_c_ 86.2) of the furan ring. In addition, HMBC correlations from H-2’/H-6’ (δ_H_ 7.17) of the 1,4-di-substituted phenyl ring to C-7’ (δ_c_ 85.2) of furan ring proved the attachment of two phenyl rings at C-7 and C-7’ of furan ring, respectively (Fig 3A). These assignments were further confirmed by the HMBC correlations of H-7/C-2 and H-7’/ C-2’, C-6’. The position of two methyls of furan ring was elucidated using HMBC cross peaks between methine H-7’ (δ_H_ 5.38) and methyl C-9’ (δ_c_ 9.7) and between methine H-7 (δ_H_ 4.54) and methyl C-9 (δ_c_ 12.2). Finally, the relative stereochemistry of four stereogenic centers in furan ring was assigned by the 1D nuclear Overhauser effect (NOE) experiment (Fig 3B). Irradiation at δ_H_ 4.54 (H-7) gave enhanced signals at δ_H_ 6.92 (H-2), δ_H_ 0.97 (H-9) and δ_H_ 0.57 (H-9’), indicating the spatial proximity of H-2, H-9 and H-9’. In addition, irradiation at δ_H_ 5.38 (H-7’) gave enhanced signal exclusively at δ_H_ 7.17 (H-2’/H-6’), the absence of correlations between H-7’ and H-7 clearly indicated the trans configuration of the 2-substituted phenyl ring. Accordingly, the structure of **8** was established as (7R, 7’R)-7, 7’-bis(4’, 3, 4-trihydroxyphenyl)-(8R, 8’S)-8, 8’-dimethyltetrahydrofuran (Fig. S31-S38 in S1 Appendix), which is a stereoisomer of **7**.

**Fig 4:**
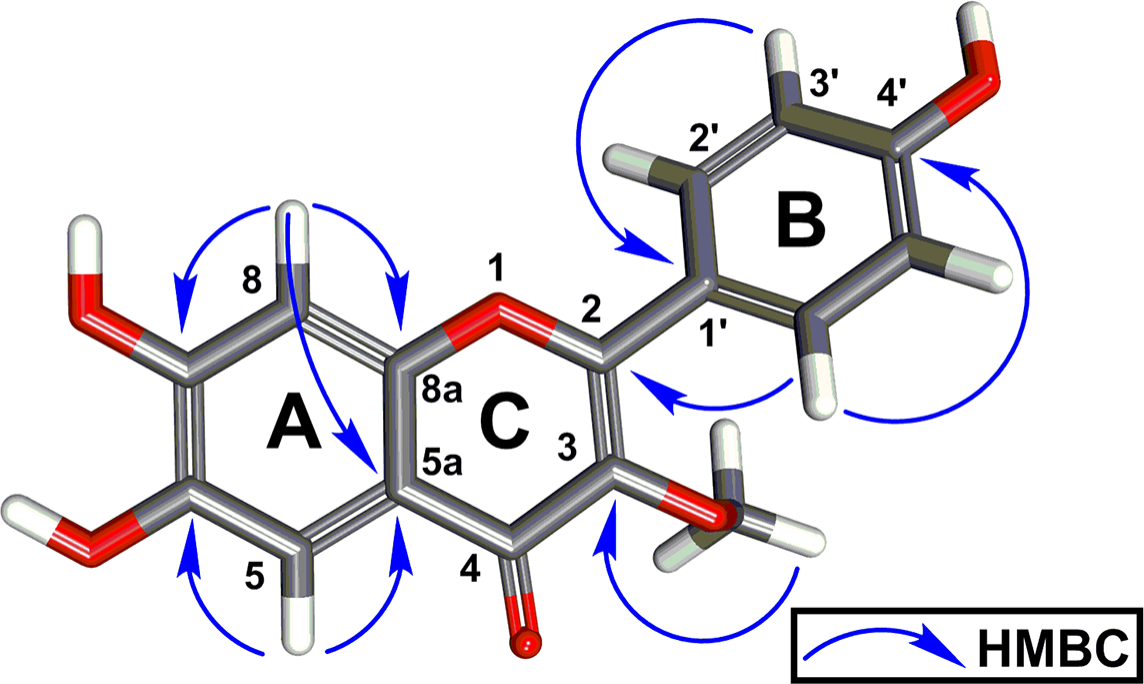
Key HMBC correlations of compound 9. The 3D energy minimized conformer was generated using BIOVIA Discovery Studio 2017 software.

Compound **9** was obtained as yellow solid and its molecular formula, C_16_H_12_O_6_, was deduced by HRMS as well as ^1^H and ^13^C NMR analysis. In the ^1^H NMR (CDCl_3_ + CD_3_OD) spectrum, a methoxy functionality [δ_H_ 3.74 s] was observed as well as six aromatic methines including two singlets [δ_H_ 6.35 s, 6.20, s] and an A_2_B_2_ splitting pattern [δ_H_ 7.93 d (8.6), 2H; 6.88 d (8.6), 2H] resulting from a 1,4-disubstituted phenyl ring. The ^13^C NMR (Table 2) showed sixteen carbon signals and DEPT-90 in conjunction with HMQC supported the presence of one methoxy (δ_c_ 60.1) and six aromatic methines of which four [δ_c_ 130.3 x 2 and 115.6 x 2] correlated to two doublet signals giving an A_2_B_2_ pattern. In addition, we observed two signals that correlated with two aromatic proton singlets of the tetra-substituted phenyl ring (δ_c_ 98.9 and 94.1). The remaining nine quaternary ^13^C NMR signals include a carbonyl (δ_c_ 178.8), six aromatic and two olefinic carbons (δ_c_ 163.9, 161.5, 159.7, 157.0, 156.5, 138.4, 121.7 and 105.2). These NMR data were consistent with a flavonol ring system containing three hydroxyls and one methoxy group. The HMBC cross peaks observed between the aromatic protons in the A-ring with H-8 (δ_H_ 6.35), C-7 (δ_c_ 163.9), C-8a (δ_c_ 157.0), and C-5a (δ_c_ 105.2) (Fig 4). Cross peaks were also observed between proton H-5 (δ_H_ 6.20), C-6 (δ_c_ 161.5), and C-5a (δ_c_ 105.2) suggesting the attachment of two hydroxyl groups at C-7 (δ_c_ 163.9) and C-6 (δ_c_ 161.5). In addition, these cross peaks indicated an oxygen attachment to C-8a (δ_c_ 157.0), signifying the O-1 position of the flavonol C-ring. The flavonol B and C ring connectivity were elucidated using HMBC correlations between protons H-2’/H6’ (δ_H_ 7.93) and carbons C-2 (δ_c_ 156.5), and C-4’ (δ_c_ 159.7). The phenolic substitution on ring B was indicated through H-3’/H-5’ (δ_H_ 6.88) and carbon C-1’ (δ_c_ 121.7) correlations. Finally, the HMBC cross peak between methoxy protons (δ_H_ 3.74) and C-3 (δ_c_ 138.4) indicated that attachment at the C-3 position of the flavonol C-ring (Fig. S23-S33 in S1 Appendix) [58]. Therefore, we have precisely determined compound **9** to be 3-methoxy-6, 7, 4’-trihydroxyflavonol.

### Biological activity

**Fig 5:**
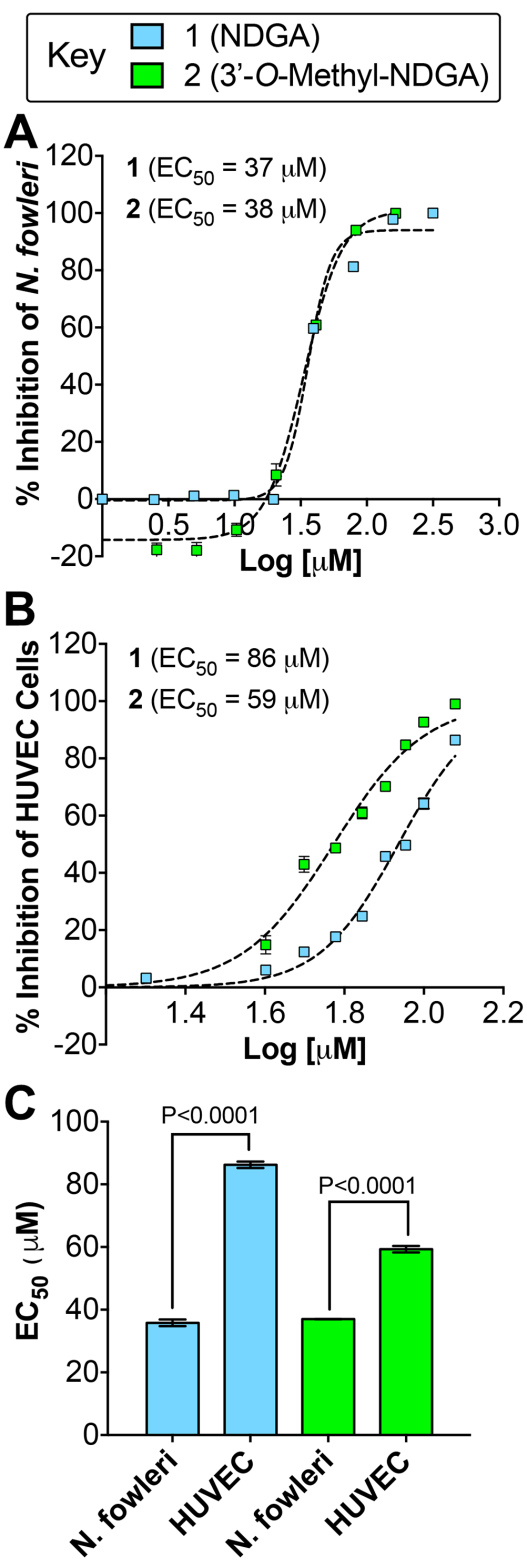
Percent inhibition of *N*. *fowleri* and human HUVEC cell proliferation by 1 and 2. (A) The EC_50_ dose response curves for **1** and **2** against *N*. *fowleri* trophozoites. (B) The EC_50_ dose response curves for **1** and **2** against HUVEC cells. (C) Compound **1** and **2** displayed more potent inhibition of *N*. *fowleri* proliferation compared to HUVEC cells, which was statistically significant by Student’s t test analysis.

We previously developed a high-throughput screening CellTiter-Glo ATP bioluminescence-based assay to assess antiparasitic activity [48], and used this assay to test compounds **1–9** against the trophozoite stage of *E*. *histolytica*, *G*. *lamblia*, and *N*. *fowleri*. Compounds **1–6** displayed dose response antiparasitic activity against all three pathogens by reducing the culture density by 50% (EC_50_) compared to untreated trophozoite cultures (Table 3). Compound **1** proved to be the most potent against both *G*. *lamblia* and *N*. *fowleri* (EC_50_ = 36 μM) (Fig 5). However, **1** and **2** display similar EC_50_ values, and both exhibited only moderate activity against *E*. *histolytica* with EC_50_ values of 103 μM and 171 μM, respectively. Both compounds **1** and **2** were found to be about 1.5-fold more active relative to the current standard drug miltefosine (EC_50_ = 54.5 μM) against *N*. *fowleri*. Compound **3** was more active against *G*. *lamblia* (EC_50_ = 49 μM) than *E*. *histolytica* (EC_50_ = 94 μM) or *N*. *fowleri* (EC_50_ = 73 μM), whereas compound **4** had similar activity against all three pathogens with EC_50_ values from 74 μM to 83 μM. Compounds **5** and **6** had comparatively weak activity against the three pathogens. Similarly, **9** displayed modest antiparasitic activity against *G*. *lamblia* (EC_50_ = 153 μM) and *N*. *fowleri* (EC_50_ = 235 μM) (Table 3). Larreatricin derivatives and stereoisomers **7** and **8** displayed no antiparasitic activity.

To further assess the therapeutic potential of **1** and **2**, which displayed the most potent antiparasitic activity agains *N*. *fowleri*, we conducted a cytotoxicity study with human umbilical vein endothelial cells (HUVEC), using the same CellTiter-Glo assay and time course that we used for assessing trophozoite toxicity (Fig 5B). Compounds **1** and **2** inhibit HUVEC cell viability with EC_50_ values of 86 μM and 59 μM, respectively. Thus, **1** and **2** are correspondingly 2.4 fold and 1.6 fold less toxic to human cells compared to *N*. *fowleri*, which is statistically significant (P<0.0001) (Fig 5C).

### Cysteine protease activity

**Fig 6:**
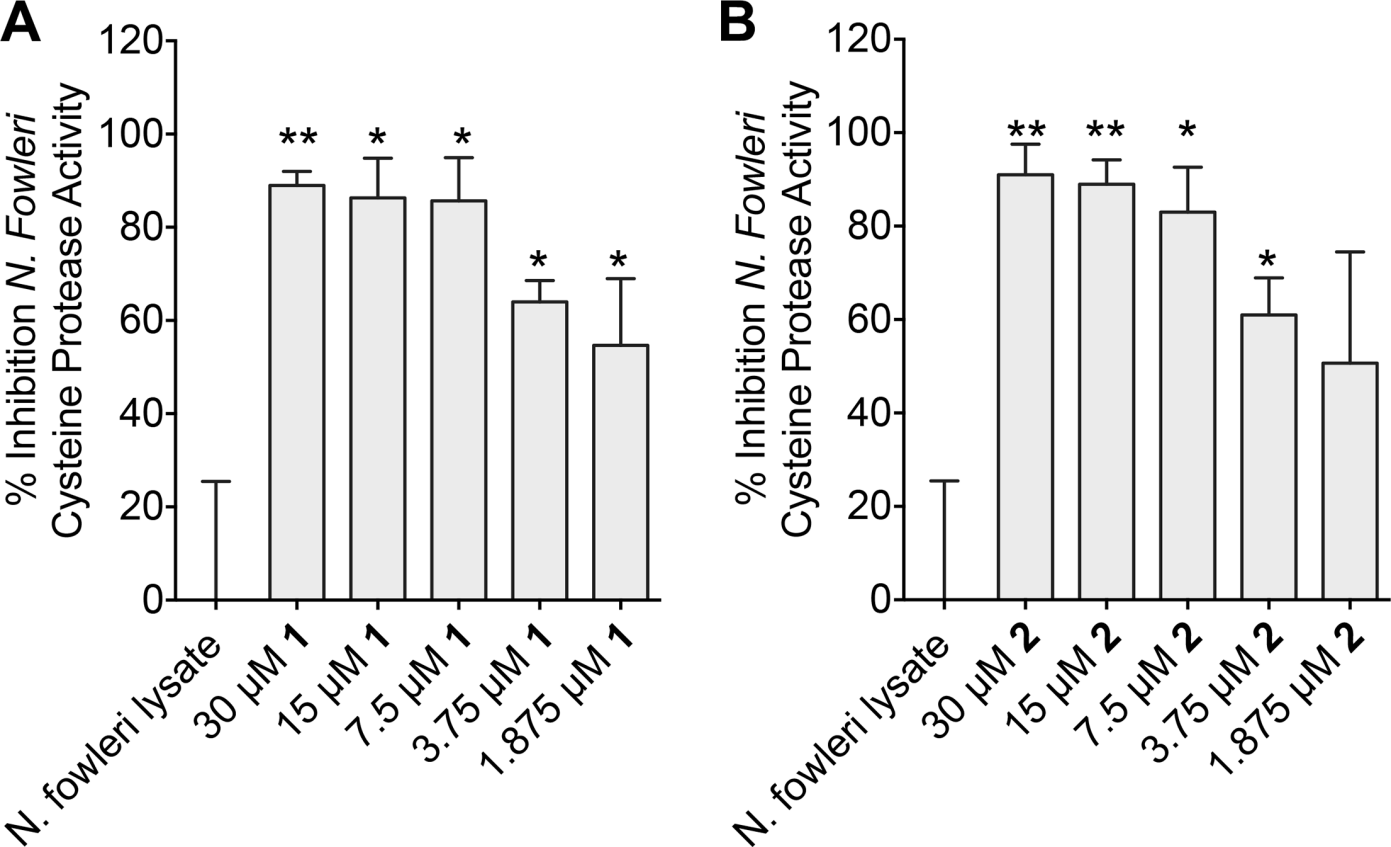
Percent Inhibition of cysteine protease activity present in *N*. *fowleri* crude extract after treating the cells with different concentrations of compound **1** (A) and compound **2** (B). One microgram of lysate protein was used in the cysteine protease assay. Cysteine protease activity was determined as described in Experimental Section and measured as RFU/min/μg protein. The data represent the mean and standard error of mean of three independent experiments. *P < 0.05 by Student’s t test compared to DMSO-treated *N*. *fowleri* lysate.

NDGA was previously shown to inhibit cysteine protease in cancer [59], and recent studies linked the involvement of cysteine protease in the pathogenesis of *N*. *fowleri* [45]. Thus, we investigated the effects of compounds **1** and **2** on cysteine protease activity present in total crude lysate of *N*. *fowleri* over a concentration range from 1.875-to-30 μM. The dose dependent effect varied between **1** and **2**, however, both inhibited the cysteine protease activity by almost 50% at 1.875 μM (Fig 6). This data indicates that the activity of compounds **1** and **2** against whole cell *N*. *fowleri* may be due to the modulation of cysteine protease activity present in the trophozoites.

## Discussion

Because lignans **1–6** are from the same structural class of compounds we could assess notable structure activity relationships (SAR). For example, **1** and **2** displayed overall more potent activity compared to **3–6**, which may be a result of the more flexible straight chain structure that offers more conformational flexibility compared to **3–6**. In addition, introducing a methoxy group in the 3’-position of **2** appears to be negligible with regard to SAR. Conversely, **3** and **4** only differ by one methoxy group in the 3’ position (i.e. compound **4**), which reduced the antiparasitic activity against *G*. *lamblia* by ~2 fold. However, this functional group was dispensable when comparing the activity between *E*. *histolytica* and *N*. *fowleri*. Similarly, introducing a phenol in the 3’ position as in **6** also results in reduced activity compared to **3**. The most striking SAR is observed by introducing a methoxy group in the seven position such as in **5**, which results in a substantial loss of activity compared to **3**: ~3 fold (*E*. *histolytica*), 4-fold (*G*. *lamblia*), and ~ 2 fold (*N*. *fowleri*). Although **1–6** are proposed to be biosynthesized from **7** and **8** [44] and share many of the same structural features, these compounds displayed no antiparasitic activity. To better understand this SAR we compared the calculated LogP values for **1**–**9**. Compounds **7** and **8** are 10 fold more hydrophilic (CLogP = 3.5) compared to **1–6** (CLogP = 4.5). However, the flavonol **9** (CLogP = 1.1) is 1,000 fold more hydrophilic compared to **7** and **8**. Interestingly, flavonoids are known to actively diffuse through organism membranes via membrane transporters such as the ATP-binding cassette (ABC) transporters [60]. Moreover, parasitic protozoa are known to express these ABC transporters and other relevant transporters utilized by flavonoids [61], which may explain the activity of **9** compared to **7** and **8**. Thus, it is plausible that the difference in hydrophilicity may be a physical property of **7** and **8** preventing diffusion into the parasite trophozoites, explaining their inactivity compared to **1**–**6** and **9**.

Compounds **1** and **2** did not display more potent activity against *E*. *histolytica* and *G*. *lamblia* compared to metronidazole, but both compounds where 1.5 fold more potent against *N*. *fowleri* compared to miltefosine, which is used for the treatment of PAM. Therefore, we selected *N*. *fowleri* for follow-up studies with compounds **1** and **2**. Interestingly, although NDGA has been shown to be cytotoxic to tumor cells by inducing apoptosis and possess antiviral activity [62, 63], it has also been shown to be a neuroprotective agent and protective of human monocytes and other human cells and tissues through its powerful antioxidant activity [62–65]. However, at high doses, NDGA has been shown to display nephrotoxicity and hepatotoxicity [62]. Importantly, our data and the collective literature reports described herein indicate that NDGA and derivatives have some therapeutic potential against *N*. *fowleri*.

Next, we investigated a potential molecular target of NDGA by review of the literature. A report by Huang *et al*. showed that the NDGA derivatives significantly inhibited cysteine protease activity [59]. Recent studies have also reported that *N*. *fowleri* lysate contains cysteine proteases such as cathepsin B-like protease that are important virulence factors of *N*. *fowleri*. Cysteine cathepsins are also critical to invasion, evasion, immunomodulation and are implicated in the attachment mechanism to the host tissue [45, 51, 66]. Moreover, cathepsins also potentiate *N*. *fowleri* growth [66]. Based on these studies we hypothesized that **1** and **2** may be inhibitors of cysteine protease activity present in *N*. *fowleri*. Indeed, our results validated this hypothesis and show that NDGA/derivatives inhibit 50% to 80% of *N*. *fowleri* cysteine protease activity between 1.875–7.5 μM (Fig 6), which are potencies that are consistent with our antiproliferative and antiparasitic data against *N*. *fowleri* (Table 3 and Fig 5).

In conclusion, lignans **1**–**8** and flavonol **9** represent two well-known classes of plant secondary metabolites [67, 68]. The well-studied flavonoid class of natural products such as **9** display a broad range of biological activity including antiparasitic activity [68, 69]. Likewise, lignan natural products have received strong interest and have been intensely studied due to their broad clinically relevant biological activity, including: antioxidant, antiviral, antibacterial, immunosuppressive, anti-inflammatory, and anticancer properties [67, 70]. Only one previous study reported in 1978 demonstrated that NDGA isolated from *L*. *tridentata* had inhibitory effect on the growth of non-pathogenic *Entamoeba* invadens [71]. Our report, for the first time, demonstrates that lignans isolated from *L*. *tridentata* are active against pathogenic *E*. *histolytica* and *G*. *lamblia*, which directly cause human amebiasis and giardiasis, respectively. Moreover, literature reports of natural products effective against *N*. *fowleri* growth have been limited [28, 72] and our study has identified relatively potent compounds from *L*. *tridentata* that have amebicidal activity against *N*. *fowleri*, which we show may be due to inhibiting cysteine protease activity present in the lysate of *N*. *fowleri*. Therefore, lignan secondary metabolites from the creosote bush represent a class of natural products pharmacophore that can be optimized through medicinal chemistry to translate more effective therapeutic options for amebiasis, giardiasis, and PAM.

## Supporting information

S1 Appendix (PDF)
