# Supplementary material for: Larrea tridentata: A novel source for anti-parasitic agents active against Entamoeba histolytica, Giardia lamblia and Naegleria fowleri
Source: PLoS Negl Trop Dis. 2017 Aug 9;11(8):e0005832. doi: 10.1371/journal.pntd.0005832 (PMC5565192; doi:10.1371/journal.pntd.0005832)

Appendix

Table of Contents

Table S1. ^{1}H NMR (400 MHz) and ^{13}C NMR (100 MHz) data for compound **1** in acetone-d_6 compared to the reported NMR data[1] (δ in ppm, *J* in Hz) 3

Table S2. ^{1}H NMR (400 MHz) and ^{13}C NMR (100 MHz) data for compound **2** in acetone-d_6 and $CDCl_3$ compared to the reported NMR data[2] in $CDCl_3$ (δ in ppm, *J* in Hz) 4

Table S3. ^{1}H NMR (400 MHz) and ^{13}C NMR (100 MHz) data for compound **3** in acetone-d_6 and $CDCl_3$ compared to the reported NMR data[2] in $CDCl_3$ (δ in ppm, *J* in Hz) 5

Table S4. ^{1}H NMR (400 MHz) and ^{13}C NMR (100 MHz) data for compound **4** in acetone-d_6 and $CDCl_3$ compared to the reported NMR data[3] in $CDCl_3$ (δ in ppm, *J* in Hz) 6

Table S5. ^{1}H NMR (400 MHz) and ^{13}C NMR (100 MHz) data for compound **6** in acetone-d_6 and $CDCl_3$ compared to the reported NMR data[2] in $CDCl_3$ (δ in ppm, *J* in Hz) 7

Table S6. ^{1}H NMR (400 MHz) and ^{13}C NMR (100 MHz) data for compound **6** in CD_3OD compared to the reported NMR data[4] in acetone-d_6 (δ in ppm, *J* in Hz) 8

Table S7. ^{1}H NMR (400 MHz) and ^{13}C NMR (100 MHz) data for compound **7** in acetone-d_6 compared to the reported NMR data[5] (δ in ppm, *J* in Hz) 9

References 9

Figure S1. ^{1}H NMR spectrum of nordihydroguaiaretic acid (**1**) in $(CD_3)_2CO$ 10

Figure S2. ^{13}C NMR spectrum of nordihydroguaiaretic acid (**1**) in $(CD_3)_2CO$ 11

Figure S3. HMQC NMR spectrum of nordihydroguaiaretic acid (**1**) in $(CD_3)_2CO$ 12

Figure S4. ^{1}H NMR spectrum of 3'-*O*-methylnordihydroguairetic acid (**2**) in $(CD_3)_2CO$ 13

Figure S5. ^{1}H NMR spectrum of 3'-*O*-methylnordihydroguairetic acid (**2**) in $CDCl_3$ 14

Figure S6. ^{13}C NMR spectrum of 3'-*O*-methylnordihydroguairetic acid (**2**) in $(CD_3)_2CO$ 15

Figure S7. ^{13}C DEPT-135 NMR spectrum of 3'-*O*-methylnordihydroguairetic acid (**2**) in $(CD_3)_2CO$ 16

Figure S8. HMQC NMR spectrum of 3'-*O*-methylnordihydroguairetic acid (**2**) in $(CD_3)_2CO$ 17

Figure S9. ^{1}H NMR spectrum of 3'-demethoxy-6-*O*-demethylisoguaiacin (**3**) in $(CD_3)_2CO$ 18

Figure S10. ^{1}H NMR spectrum of 3'-demethoxy-6-*O*-demethylisoguaiacin (**3**) in $CDCl_3$ 19

Figure S11. ^{13}C NMR spectrum of 3'-demethoxy-6-*O*-demethylisoguaiacin (**3**) in $(CD_3)_2CO$ 20

Figure S12. HMQC spectrum of 3'-demethoxy-6-*O*-demethylisoguaiacin (**3**) in $(CD_3)_2CO$ 21

Figure S13. HMBC spectrum of 3'-demethoxy-6-*O*-demethylisoguaiacin (**3**) in $(CD_3)_2CO$ 22

Figure S14. ^{1}H NMR spectrum of nor-isoguaicin (**4**) in $(CD_3)_2CO$ 23

Figure S15. ^{1}H NMR spectrum of nor-isoguaicin (**4**) in $CDCl_3$ 24

Figure S16. ^{13}C NMR spectrum of nor-isoguaicin (**4**) in $(CD_3)_2CO$ 25

Figure S17. ^{13}C DEPT 135 NMR spectrum of nor-isoguaicin (**4**) in $(CD_3)_2CO$ 26

Figure S18. HMQC NMR spectrum of nor-isoguaicin (**4**) in $(CD_3)_2CO$ 27

Figure S19. ^{1}H NMR spectrum of 3'-demethoxyisoguaiacin (**5**) in $(CD_3)_2CO$ 28

Figure S20. ^{13}C NMR spectrum of 3'-demethoxyisoguaiacin (**5**) in $(CD_3)_2CO$ 29

Figure S21. ^{13}C DEPT-135 NMR spectrum of 3'-demethoxyisoguaiacin (**5**) in $(CD_3)_2CO$....................30

Figure S22. HMQC NMR spectrum of 3'-demethoxyisoguaiacin (**5**) in $(CD_3)_2CO$ 31

Figure S23. ^{1}H NMR spectrum of 6, 3'-di-*O*-demethoxy-isoguaiacin (**6**) in CD_3OD....................32

Figure S24. ^{13}C NMR spectrum of 6, 3'-di-*O*-demethoxy-isoguaiacin (**6**) in CD_3OD....................33

Figure S25. ^{13}C DEPT-135 NMR spectrum of 6, 3'-di-*O*-demethoxy-isoguaiacin (**6**) in CD_3OD 34

Figure S26. ^{1}H NMR spectrum of 3'-hydroxy-4-epi-larreatricin (**7**) in $(CD_3)_2CO$ 35

Figure S27. ^{13}C NMR spectrum of 3'-hydroxy-4-epi-larreatricin (**7**) in $(CD_3)_2CO$ 36

Figure S28. ^{13}C DEPT-135 NMR spectrum of 3'-hydroxy-4-epi-larreatricin (**7**) in $(CD_3)_2CO$....................37

Figure S29. ^{13}C DEPT-90 NMR spectrum of 3'-hydroxy-4-epi-larreatricin (**7**) in $(CD_3)_2CO$....................38

Figure S30. HMQC NMR spectrum of 3'-hydroxy-4-epi-larreatricin (**7**) in $(CD_3)_2CO$ 39

Figure S31. ^{1}H NMR spectrum of (7R, 7'R)-7, 7'-bis(4', 3, 4-trihydroxyphenyl)-(8R, 8'S)-8, 8'-dimethyltetrahydrofuran (**8**) in $(CD_3)_2CO$ 40

Figure S32. ^{13}C NMR spectrum of of (7R, 7'R)-7, 7'-bis(4', 3, 4-trihydroxyphenyl)-(8R, 8'S)-8, 8'-dimethyltetrahydrofuran (**8**) in $(CD_3)_2CO$ 41

Figure S33. ^{13}C DEPT-135 NMR spectrum of of (7R, 7'R)-7, 7'-bis(4', 3, 4-trihydroxyphenyl)-(8R, 8'S)-8, 8'-dimethyltetrahydrofuran (**8**) in $(CD_3)_2CO$ 42

Figure S34. ^{13}C DEPT-90 NMR spectrum of of (7R, 7'R)-7, 7'-bis(4', 3, 4-trihydroxyphenyl)-(8R, 8'S)-8, 8'-dimethyltetrahydrofuran (**8**) in $(CD_3)_2CO$....................43

Figure S35. HMQC NMR spectrum of (7R, 7'R)-7, 7'-bis(4', 3, 4-trihydroxyphenyl)-(8R, 8'S)-8, 8'-dimethyltetrahydrofuran (**8**) in $(CD_3)_2CO$ 44

Figure S36. HMBC NMR spectrum of of (7R, 7'R)-7, 7'-bis(4', 3, 4-trihydroxyphenyl)-(8R, 8'S)-8, 8'-dimethyltetrahydrofuran (**8**) in $(CD_3)_2CO$ 45

Figure S37. NOESY NMR spectrum of of (7R, 7'R)-7, 7'-bis(4', 3, 4-trihydroxyphenyl)-(8R, 8'S)-8, 8'-dimethyltetrahydrofuran (**8**) in $(CD_3)_2CO$ 46

Figure S38. NOESY NMR spectrum of of (7R, 7'R)-7, 7'-bis(4', 3, 4-trihydroxyphenyl)-(8R, 8'S)-8, 8'-dimethyltetrahydrofuran (**8**) in $(CD_3)_2CO$ 47

Figure S39. ^{1}H NMR spectrum of 3-methoxy-6, 7, 4'-trihydroxyflavone (**9**) in $CDCl_3$ and CD_3OD........48

Figure S40. ^{13}C NMR spectrum of 3-methoxy-6, 7, 4'-trihydroxyflavone (**9**) in $CDCl_3$ and CD_3OD.......49

Figure S41. ^{13}C DEPT-135 NMR spectrum of 3-methoxy-6, 7, 4'-trihydroxyflavone (**9**) in $CDCl_3$ and CD_3OD....................50

Figure S42. ^{13}C DEPT-90 NMR spectrum of 3-methoxy-6, 7, 4'-trihydroxyflavone (**9**) in $CDCl_3$ and CD_3OD....................51

Figure S43. HMQC NMR spectrum of 3-methoxy-6, 7, 4'-trihydroxyflavone (**9**) in $CDCl_3$ and CD_3OD 52

Figure S44. HMBC NMR spectrum of 3-methoxy-6, 7, 4'-trihydroxyflavone (**9**) in $CDCl_3$ and CD_3OD 53

Table S1. ^{1}H NMR (400 MHz) and ^{13}C NMR (100 MHz) data for compound **1** in acetone-d_6 compared to the reported NMR data[1] (δ in ppm, *J* in Hz)

Position	**1**		Reported	
	δ_H	δ_C	δ_H	δ_C
1a/4a	2.21 dd (13.3, 9.2)	39.2 CH_2	2.20 dd (9.0, 4.2)	39.2 CH_2
1b/4b	2.70 dd (13.3, 5.0)	-	2.68 dd (8.4, 4.8)	-
2/3	1.74 m	40.2 CH	1.72 m	40.1 CH
5/6	0.83 d (6.64)	16.6 CH_3	0.82 d (6.64)	16.5 CH_3
1'/1"	-	134.4 C	-	134.4 C
2'/2"	6.69 d (1.8)	115.8 CH	6.68 d (1.8)	115.8 CH
3'/3"	-	143.7 C	-	143.7 C
4'/4"	-	145.6 C	-	145.6 C
5'/5"	6.73 d (7.9)	116.9 CH	6.73 d (7.8)	116.9 CH
6'/6"	6.52 dd (7.9, 1.8)	121.3 CH	6.52 dd (6.0, 1.8)	121.3 CH

Table S2. ^{1}H NMR (400 MHz) and ^{13}C NMR (100 MHz) data for compound **2** in acetone-d_6 and $CDCl_3$ compared to the reported NMR data[2] in $CDCl_3$ (δ in ppm, *J* in Hz)

Position	**2**[a]		**2**[b]	Reported[b]
	δ_H	δ_C	δ_H	δ_C
1	2.25 dd[c]	39.1 CH_2	2.25 dd (13.1, 9.3)	2.25 dd (13.4, 9.3)
	2.75 dd (13.4, 4.8)		2.71 dd (13.3, 4.8)	2.71 dd (13.4, 4.7)
2,3	1.75 m	39.9, 40.0 CH	1.73 m	1.72 m
4	2.24 dd[c]	39.3 CH_2	2.25 dd (13.3, 9.4)	2.25 dd (13.5, 9.3)
	2.69 dd (13.4, 5.40		2.68 dd (13.3, 5.0)	2.68 dd (13.5, 5.2)
5,6	0.82 d, 0.84 d (6.8)	16.4, 16.6 CH_3	0.82 d, 0.83 d (6.6)	0.82 d, 0.83 d (6.7)
1’	-	134.1 C	-	-
2’	6.71 d (1.9)	116.9 CH	6.61 brd (1.9)	6.61 d (1.9)
3’	-	148.1 C	-	-
4’	-	145.4 C	-	-
5’	6.75 d (8.2)	115.4 CH	6.82 d (8.0)	6.82 d (8.0)
6’	6.63 dd (8.0, 1.9)	122.3 CH	6.64 dd (8.0, 1.9)	6.64 dd (8.0, 1.9)
1”	-	134.3 C	-	-
2”	6.75 d (2.1)	113.2 CH	6.67 brd (2.0)	6.67 d (2.0)
3”	-	143.8 C	-	-
4”	-	145.7 C	-	-
5”	6.73 d (8.0)	115.8 CH	6.77 d (8.0)	6.77 d (8.0)
6”	6.54 dd (7.9, 1.6)	121.2 CH	6.58 brdd (8.0, 2.0)	6.58 dd (8.0, 2.0)
OMe	3.81 s	56.1 OCH_3	3.86 s	3.86 s
OH	7.58 brs		5.03 brs, 5.45 s	5.00, 5.15, 5.45 s

[a] In acetone-d_6

[b] In $CDCl_3$

[c] Overlapped signals

Table S3. ^1H NMR (400 MHz) and ^{13}C NMR (100 MHz) data for compound **3** in acetone-d_6 and $CDCl_3$ compared to the reported NMR data[2] in $CDCl_3$ (δ in ppm, J in Hz)

Position	**3**[a]		**3**[b]	Reported[b]
	δ_H	δ_C	δ_H	δ_H
1	3.59 d (6.3)	50.8 CH	3.57 d (6.2)	3.59 d (6.2)
2	1.89 m	41.8 CH	1.89 m	1.90 m
3	2.03 m	30.1 CH	1.99 m	2.01 m
4a	2.81 dd (16.2, 5.2)	35.7 CH_2	2.83 dd (16.4, 5.5)	2.84 dd (16.3, 5.3)
4b	2.39 dd (16.2, 7.2)		2.41 dd (16.4, 7.2)	2.42 dd (16.3, 7.2)
5	6.56 s	115.9 CH	6.60 s	6.62 s
6	-	144.0 C	-	-
7	-	144.4 C	-	-
8	6.23 s	117.7 CH	6.29 s	6.31 s
9	-	128.1 C	-	-
10	-	130.7 C	-	-
11	0.88 d (6.9)	16.1 CH_3	0.87 d (6.9)	0.87 d (6.9)
12	0.89 d (6.9)	16.3 CH_3	0.88 d (6.9)	0.88 d (6.9)
1’	-	139.3 C	-	-
2’	6.85 d (8.4)	130.8 CH	6.86 d (8.5)	6.89 d (8.5)
3’	6.71 d (8.4)	115.7 CH	6.69 d (8.5)	6.72 d (8.5)
4’	-	156.3 C	-	-
5’	6.71 d (8.4)	115.7 CH	6.69 d (8.5)	6.72 d (8.5)
6’	6.85 d (8.4)	130.8 CH	6.86 d (8.5)	6.89 d (8.5)
OH	8.06, 7.46, 7.43		5.25 s, 5.35 s	very broad

[a] In acetone-d_6

[b] In $CDCl_3$

Table S4. ^{1}H NMR (400 MHz) and ^{13}C NMR (100 MHz) data for compound **4** in acetone-d_6 and $CDCl_3$ compared to the reported NMR data[3] in $CDCl_3$ (δ in ppm, J in Hz)

Position	**4**[a]		**4**[b]		Reported[b]	
	δ_H	δ_C	δ_H	δ_C	δ_H	δ_C
1	3.55 d (6.8)	51.0 CH	3.56 d (6.8)	50.5 CH	3.56 d (6.5)	50.3 CH
2	2.01 m	30.4 CH	2.0 m	29.4 CH	2.0 dd (6.6, 3.0)	29.1 CH
3	1.94 dt (6.9, 3.0)	41.5 CH	1.91 dt (6.8, 3.0)	40.7 CH	1.91 dt (6.9, 6.5)	40.5 CH
4a	2.39 dd (16.3, 6.9	35.7 CH_2	2.41 dd (16.4, 7.0)	35.1 CH_2	2.41 dd (16.5, 6.9)	34.8 CH_2
4b	2.84 dd (16.3, 5.3		2.83 dd (16.4, 5.3)		2.83 dd (16.5, 6.9)	
5	6.56 s	115.8 CH	6.32 s	115.0 CH	6.29 s	114.7 CH
6	-	143.8 C	-	141.4 C	-	142.1 C
7	-	144.3 C	-	142.1 C	-	142.5 C
8	6.25 s	117.5 CH	6.61 s	116.8 CH	6.58 s	116.6 CH
9	-	128.0 C	-	129.3 C	-	129.7 C
10	-	130.4 C	-	131.0 C	-	128.3 C
11	0.874 d (6.9)	16.2 CH	0.87 d (6.8)	15.9 CH_3	0.87 d (6.9)	15.6 CH_3
12	0.867 d (6.9)	15.9 CH	0.88 d (6.8)	16.0 CH_3	0.88 d (6.9)	15.7 CH_3
1'	-	139.7 C	-	139.2 C	-	139.3 C
2'	6.70 d (2.0)	113.3 CH	6.52 d (1.8)	111.3 CH	6.54 s	111.8 CH
3'	-	148.0 C	-	146.4 C	-	146.4 C
4'	-	145.5 C	-	143.8 C	-	143.4 C
5'	6.71 8 (8.1)	115.1 CH	6.79 d (8.0)	113.6 CH	6.74 d (7.8)	113.8 CH
6'	6.46 dd (8.1, 2.0)	122.5 CH	6.50 dd (8.0, 1.8)	121.9 CH	6.4 dd (8.1, 1.9)	121.8 CH
OMe	3.76	56.2 OCH_3	3.80 s	55.8 OCH_3	3.79 s	55.7 OCH_3

[a] In acetone-d_6

[b] In $CDCl_3$

Table S5. ^{1}H NMR (400 MHz) and ^{13}C NMR (100 MHz) data for compound **6** in acetone-d_6 and $CDCl_3$ compared to the reported NMR data[2] in $CDCl_3$ (δ in ppm, J in Hz)

Position	**5**[a]		**5**[b]	Reported[b]
	δ_H	δ_C	δ_H	δ_H
1	3.59 d (6.4)	50.8 CH	3.61 d (6.1)	3.61 d (6.2)
2	1.90 m	41.7 CH	1.90 m	1.90 m
3	2.02 m	30.0 CH	2.0 m	2.02 m
4a	2.88 dd (16.3, 5.4)	35.8 CH_2	2.87 dd (6.2, 5.0)	2.87 dd (16.2, 7.2)
4b	2.45 dd (16.3, 7.3)		2.45 dd (16.2, 7.2)	2.45 dd (16.2, 7.2)
5	6.66 s	112.3 CH	6.57 s	6.57 s
6	-	145.4 C	-	-
7	-	156.3 C	-	-
8	6.26 s	117.4 CH	6.39 s	6.39 s
9	-	127.6 C	-	-
10	-	131.5 C	-	-
11	0.87 d (6.9)	16.0 CH_3	0.88 d (6.9)	0.88 d (6.9)
12	0.88 d (6.9)	16.2 CH_3	0.89 d (6.9)	0.89 d (8.9)
1'	-	139.0 C	-	-
2'	6.86 d (8.5)	130.7 CH	6.87 d (8.5)	6.87 d (8.5)
3'	6.72 d (8.5)	115.6 CH	6.70 d (8.5)	6.70 d (8.5)
4'	-	146.8 C	-	-
5'	6.72 d (8.5)	115.6 CH	6.70 d (8.5)	6.70 d (8.5)
6'	6.86 d (8.5)	130.7 CH	6.87 d (8.5)	6.87 d (8.5)
OMe	3.80 s	56.1 OCH_3	3.86 s	3.86
OH	8.0, s, 7.1, s		4.85 s, 5.32 s	4.78 s, 5.35 s

[a] In acetone-d_6

[b] In $CDCl_3$

Table S6. ^{1}H NMR (400 MHz) and ^{13}C NMR (100 MHz) data for compound **6** in CD_3OD compared to the reported NMR data[4] in acetone-d_6 (δ in ppm, *J* in Hz)

Position	**6**^{a}		Reported^b	
	δ_H	δ_C	δ_H	δ_C
1	3.48 d (6.4)	51.5 CH	3.51 d (6.3)	50.8 CH
2	1.88 dt (6.8, 2.8)	42.2 CH	1.88 m	41.5 CH
3	2.01 m	30.6 CH	2.02 m	29.8 CH
4a	2.38 dd (16.4, 7.2)	36.0 CH_2	2.37 dd (16.5, 7.7)	35.4 CH_2
4b	2.80 dd (16.4, 5.6)		2.80 dd (16.5, 5.4)	
5	6.50 s	116.0 or 115.8 CH	6.56 s	115.5 or 115.7 CH
6, 7	-	144.2, 144.1 C	-	143.7 C
8	6.25 s	118.1 CH	6.26 s	117.6 CH
9	-	128.5 C	-	128.0 C
10	-	140.6 C	-	140.0 C
11,12	0.88 dd (7.2, 3.2)	16.2, 16.1 CH_3	0.87 d (6.8)	16.0, 16.2 CH_3
1'	-	130.9 C	-	130.3 C
2'	6.43 d (2.0)	117.3 CH	6.44 brs	116.8 CH
3'	-	145.8 C	-	145.2 C
4'	-	144.5 C	-	144.0 C
5'	6.66 d (8.0)	116.0 or 115.8 CH	6.71 d (8.7)	115.5 or 115.7 CH
6'	6.39 dd (8.0, 2.0)	121.6 CH	6.43 dd (8.7, 2.1)	121.2 CH

^{a} In CD_3OD

^{b} In acetone-d_6

Table S7. ^{1}H NMR (400 MHz) and ^{13}C NMR (100 MHz) data for compound **7** in acetone-d_6 compared to the reported NMR data[5] (δ in ppm, J in Hz)

Position	**7**		Reported	
	δ_H	δ_C	δ_H	δ_C
1	-	135.9	-	135.8
2	6.91 d (1.9)	114.2	6.92 d (92.1)	114.2
3	-	145.8	-	145.8
4	-	145.2	-	145.1
5	6.79 d (7.5)	115.7	6.79 d (7.7)	115.7
6	6.72 dd (7.9, 1.9)	118.7	6.74 dd (7.7, 2.1)	118.7
7	4.56 d (8.9)	88.8	4.56 d (8.4)	88.8
8	1.72 m	52.0	1.72 m	52.0
9	1.01 d (6.0)	14.0	1.10 d (5.4)	14.0
1’	-	134.8	-	134.8
2’, 6’	7.23 d (7.5)	128.4	7.24	128.4
3’, 5’	6.81 d (8.6)	115.8	6.82 d (8.6)	115.8
4’	-	157.6	-	157.6
7’	4.54 d (8.9)	88.8	4.54 d (8.4)	88.8
8’	1.72 m	51.9	1.72 m	51.9
9’	0.99 d (6.0)	14.1	0.99 d (5.4)	14.1

References

(1) Gezginci, M. H.; Timmermann, B. N., *Tetrahedron Lett.* **2001,** *42*, 6083-6085.

(2) Gnabre, J.; Huang, R. C. C.; Bates, R. B.; Burns, J. J.; Caldera, S.; Malcomson, M. E.; Mcclure, K. J., *Tetrahedron* **1995,** *51*, 12203-12210.

(3) Torres, R.; Urbina, F.; Morales, C.; Modak, B.; Delle Monache, F., *J Chil Chem Soc* **2003,** *48*, 61-63.

(4) Konno, C.; Xue, H.-Z.; Lu, Z.-Z.; Ma, B.-X.; Erdelmeier, C. A. J.; Che, C.-T.; Cordell, G. A.; Soejarto, D. D.; Waller, D. P.; Fong, H. H. S., *J. Nat. Prod.* **1989,** *52*, 1113-1117.

(5) Konno, C.; Lu, Z. Z.; Xue, H. Z.; Erdelmeier, C. A. J.; Meksuriyen, D.; Che, C. T.; Cordell, G. A.; Soejarto, D. D.; Waller, D. P.; Fong, H. H. S., *J. Nat. Prod.* **1990,** *53*, 396-406.

Figure S1. ^{1}H NMR spectrum of nordihydroguaiaretic acid (**1**) in $(CD_3)_2CO$

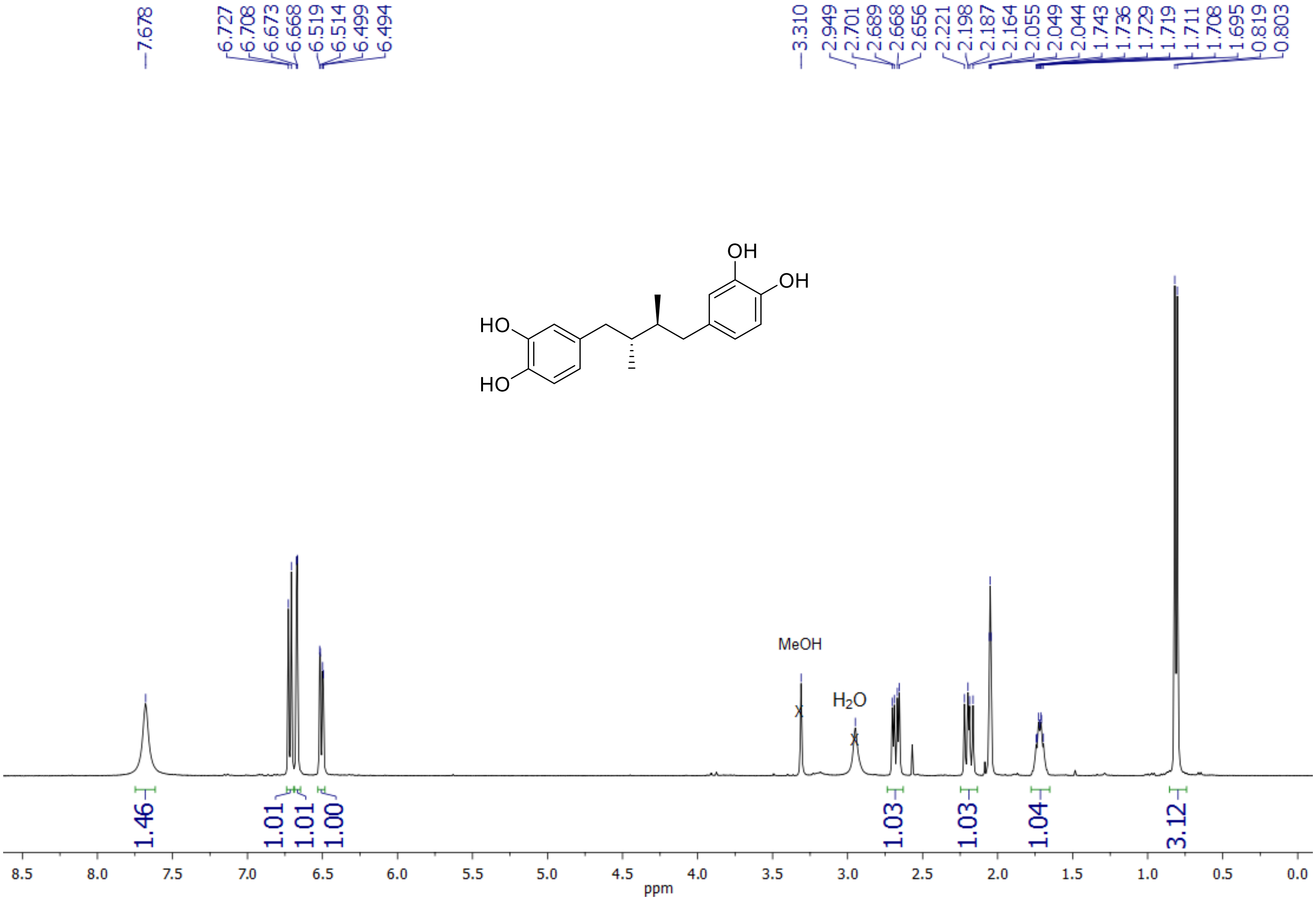

Figure S2. ^{13}C NMR spectrum of nordihydroguaiaretic acid (**1**) in $(CD_3)_2CO$

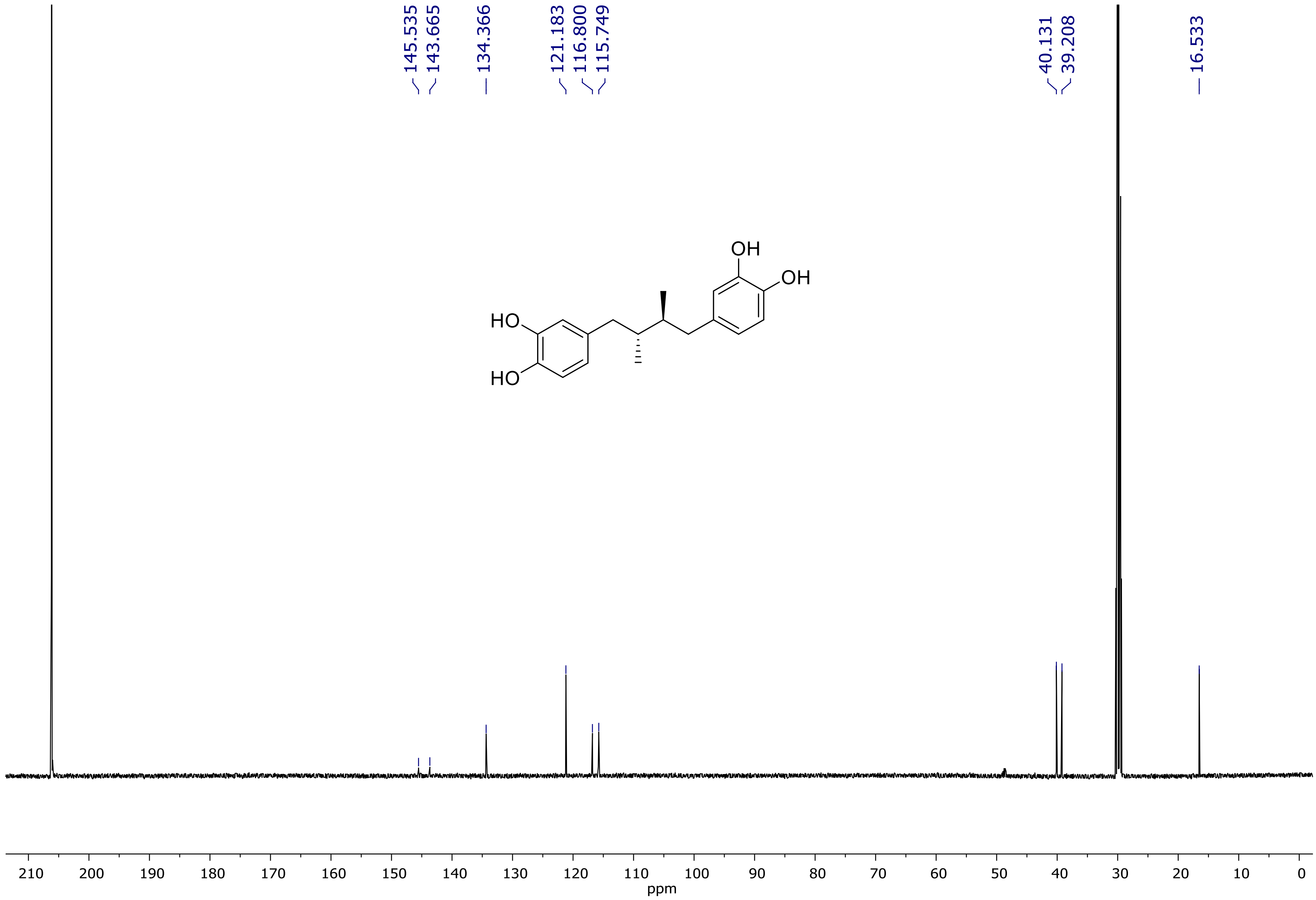

Figure S3. HMQC NMR spectrum of nordihydroguaiaretic acid (**1**) in $(CD_3)_2CO$

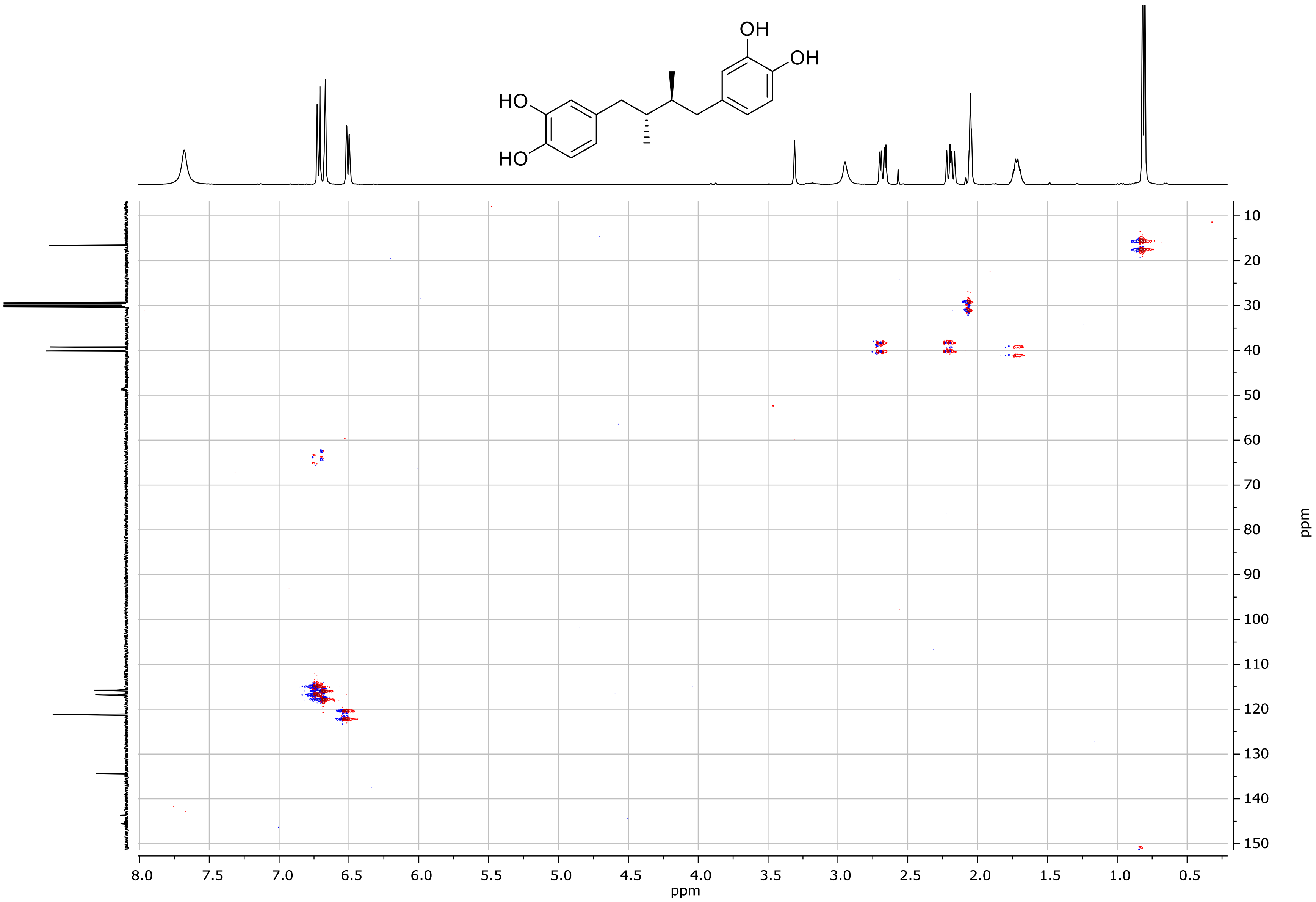

Figure S4. ^{1}H NMR spectrum of 3'-*O*-methylnordihydroguairetic acid (**2**) in $(CD_3)_2CO$

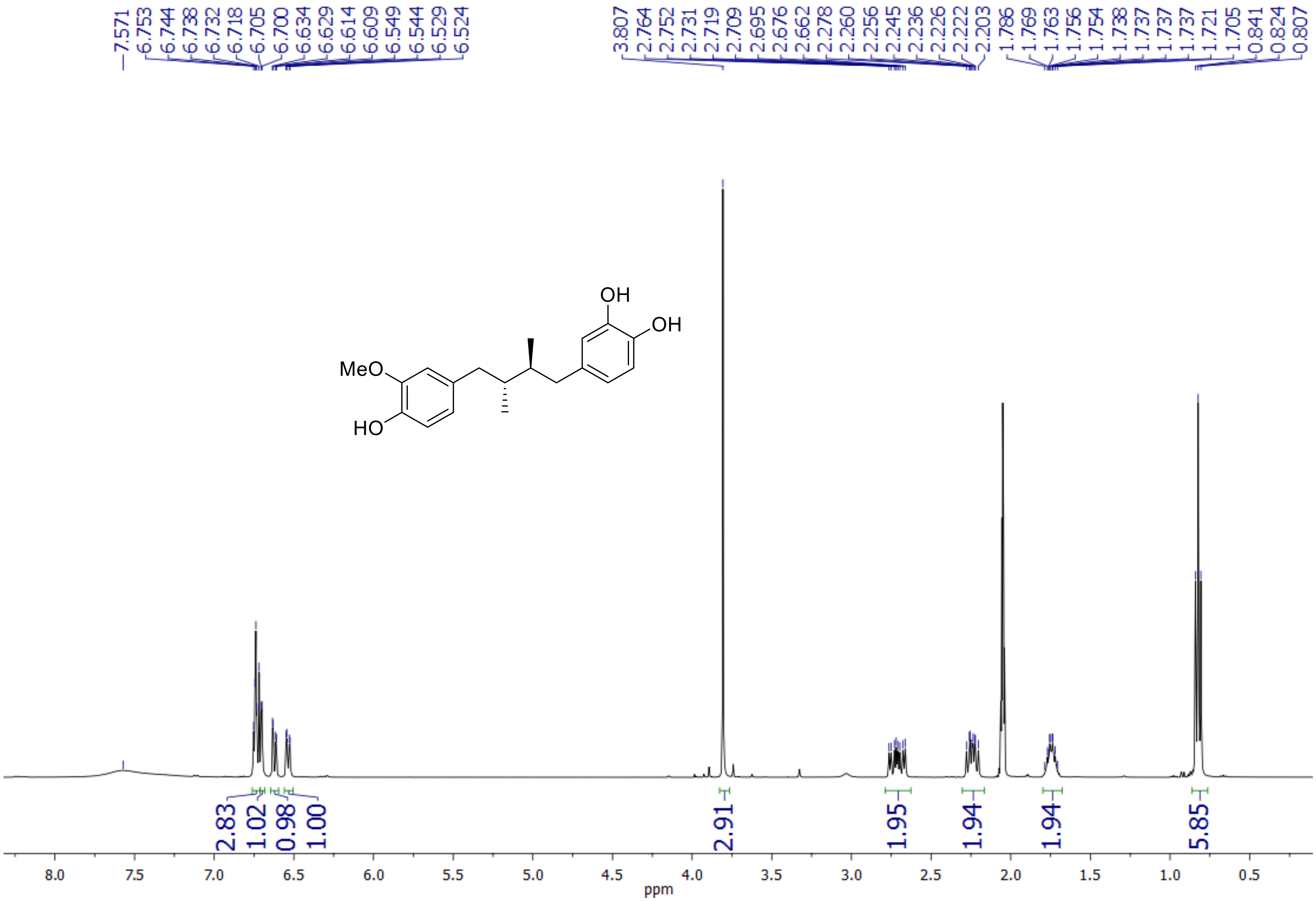

Figure S5. ^{1}H NMR spectrum of 3'-*O*-methylnordihydroguairetic acid (**2**) in $CDCl_3$

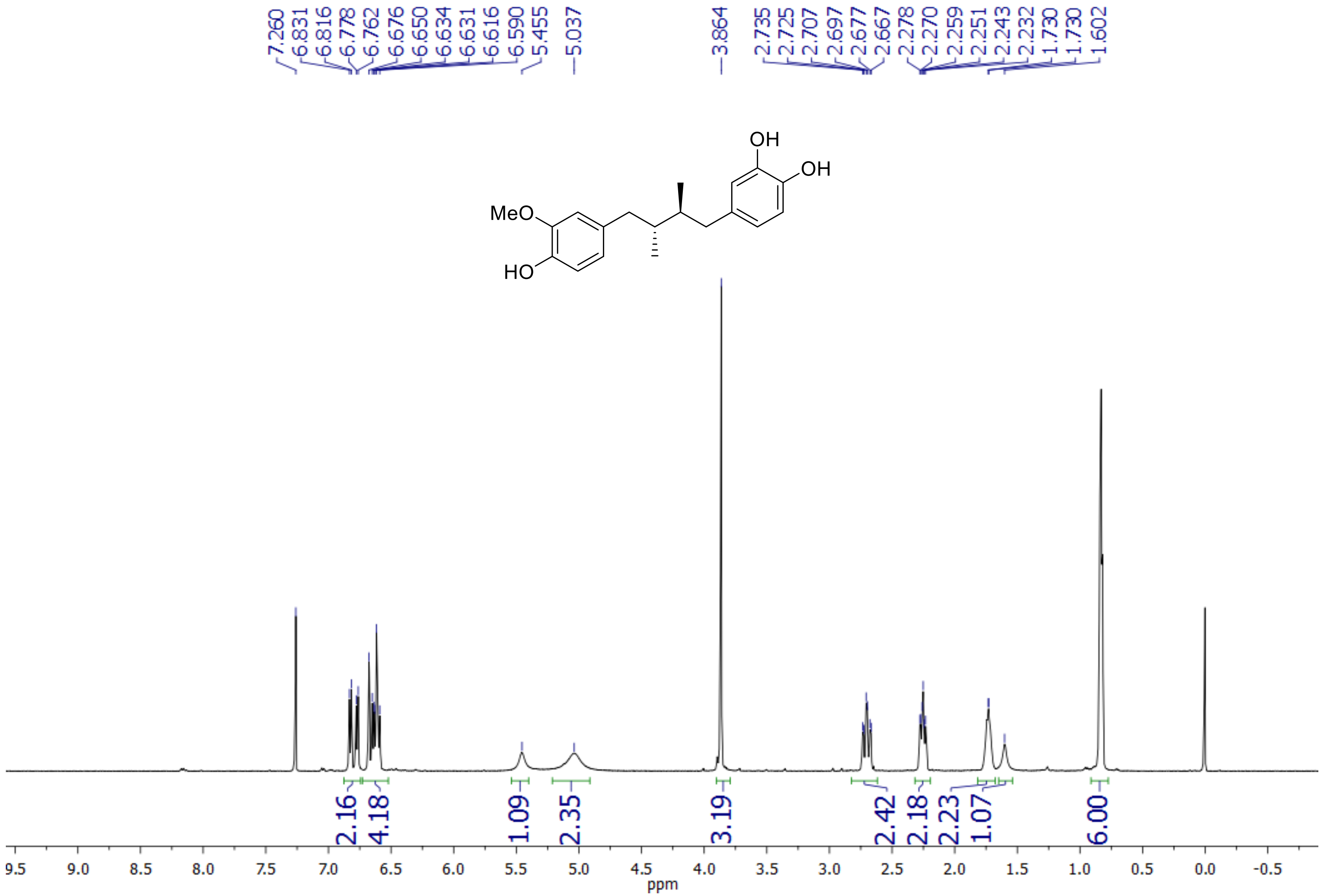

Figure S6. ^{13}C NMR spectrum of 3'-*O*-methylnordihydroguairetic acid (**2**) in $(CD_3)_2CO$

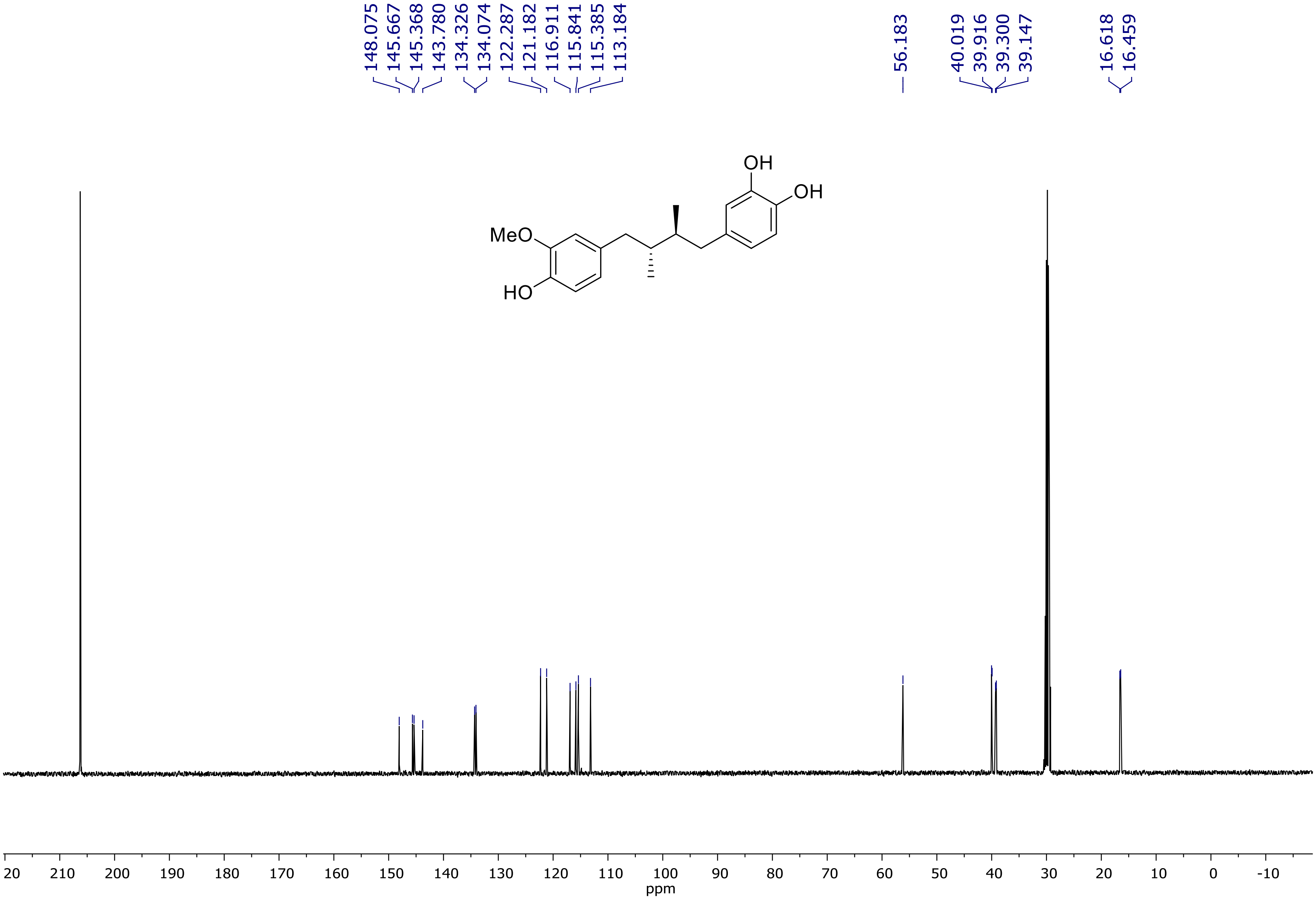

Figure S7. ^{13}C DEPT-135 NMR spectrum of 3'-*O*-methylnordihydroguairetic acid (**2**) in $(CD_3)_2CO$

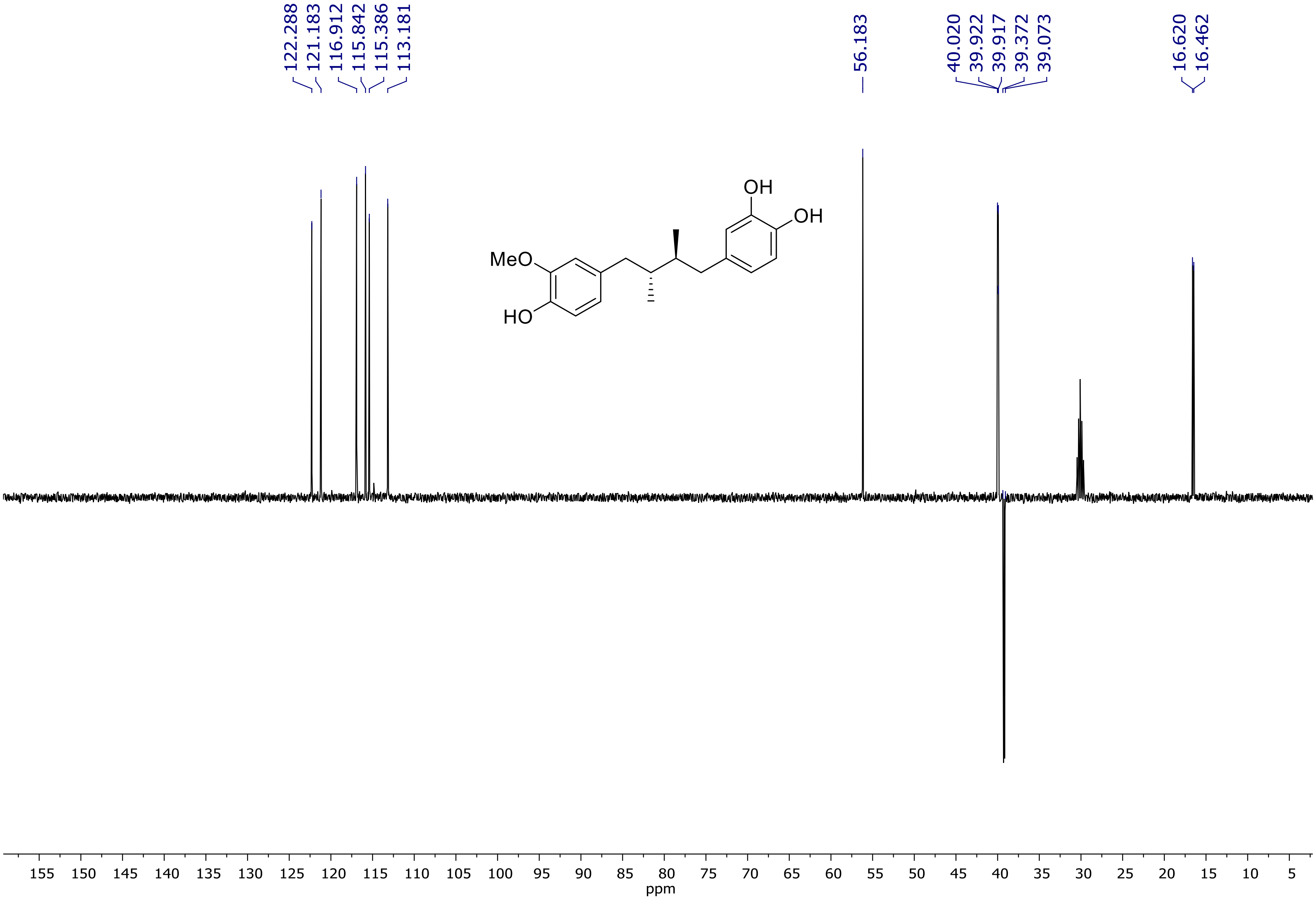

Figure S8. HMQC NMR spectrum of 3'-*O*-methylnordihydroguairetic acid (**2**) in $(CD_3)_2CO$

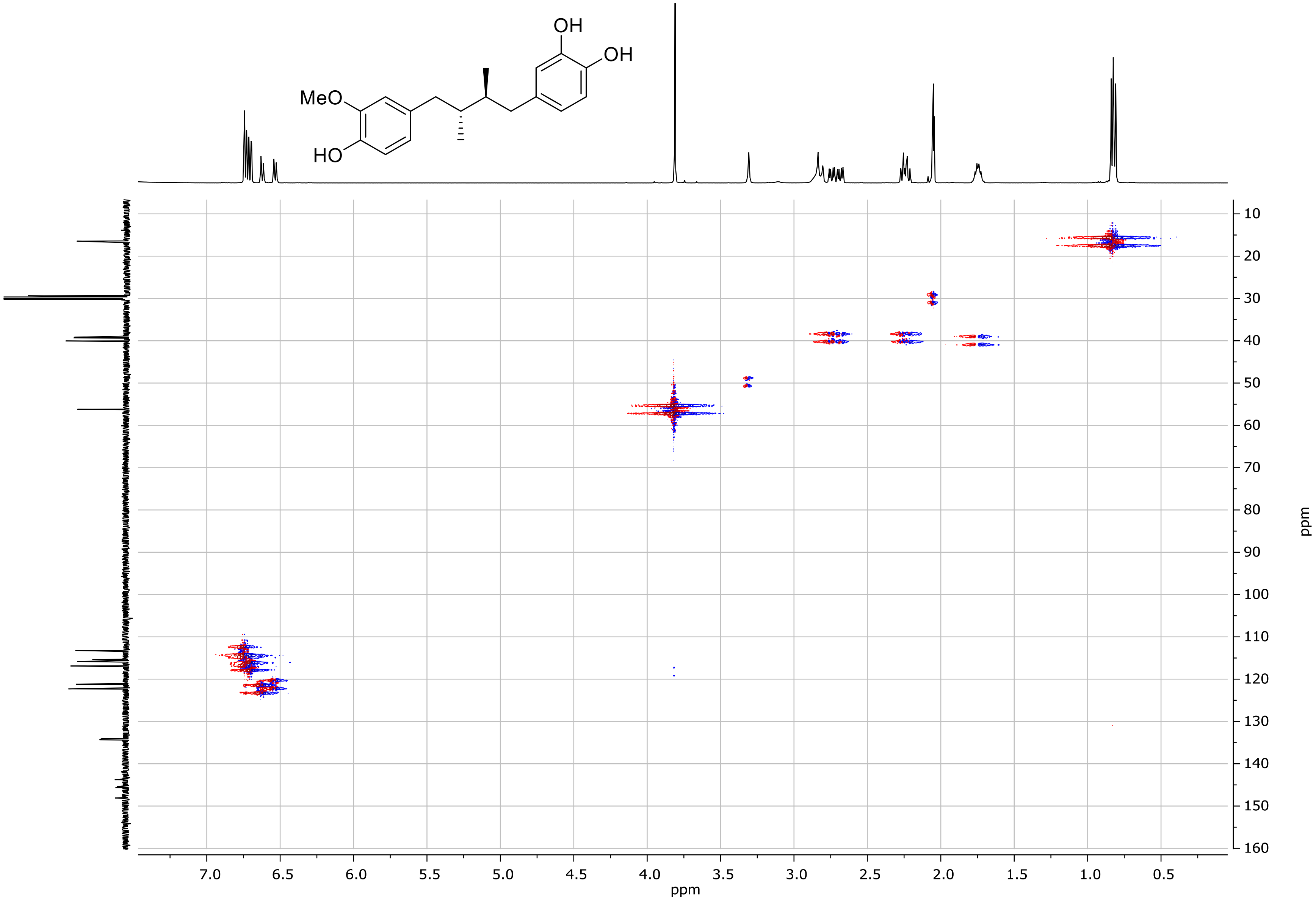

Figure S9. ^{1}H NMR spectrum of 3'-demethoxy-6-*O*-demethylisoguaiacin (**3**) in $(CD_3)_2CO$

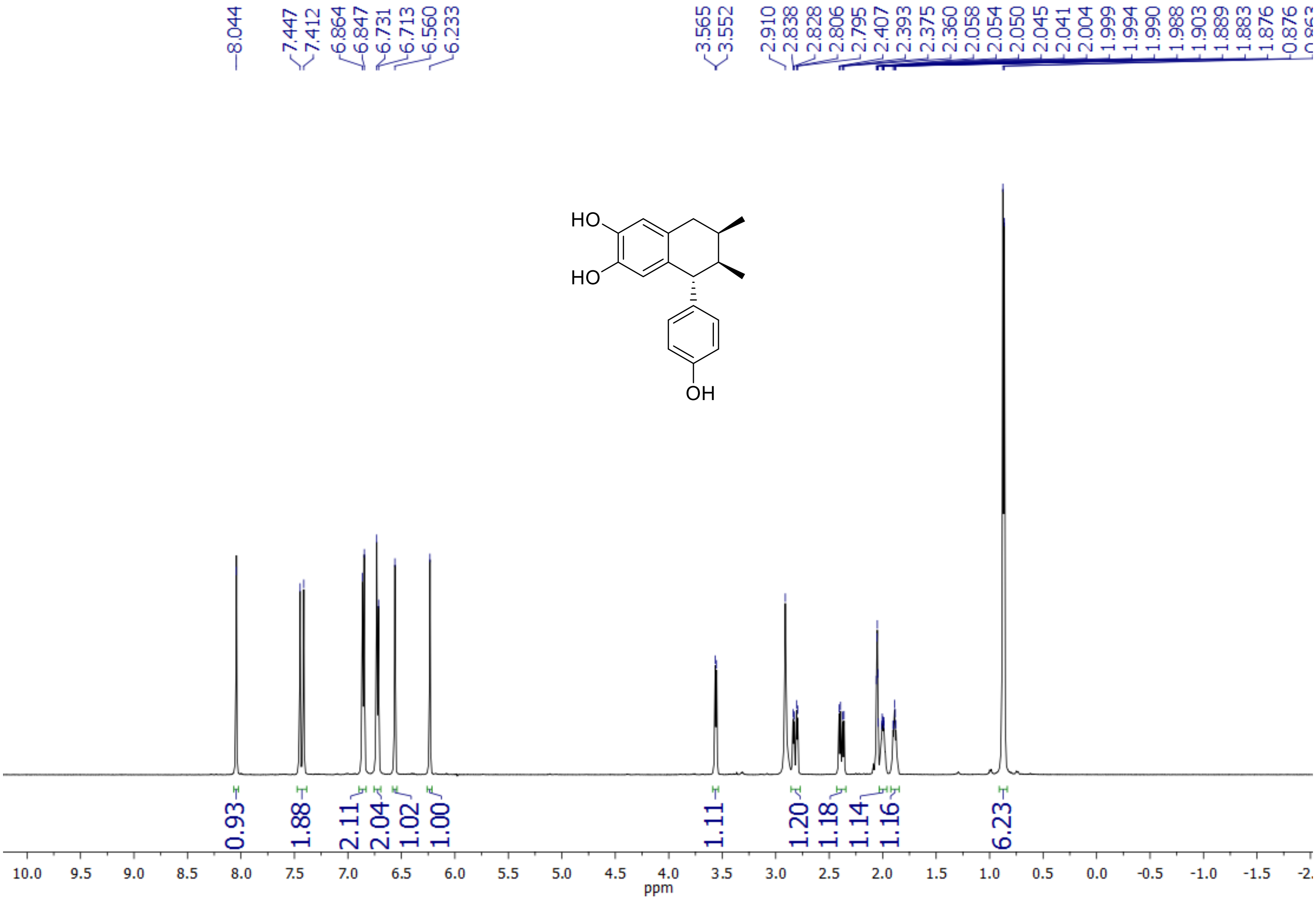

Figure S10. ^{1}H NMR spectrum of 3'-demethoxy-6-*O*-demethylisoguaiacin (**3**) in $CDCl_3$

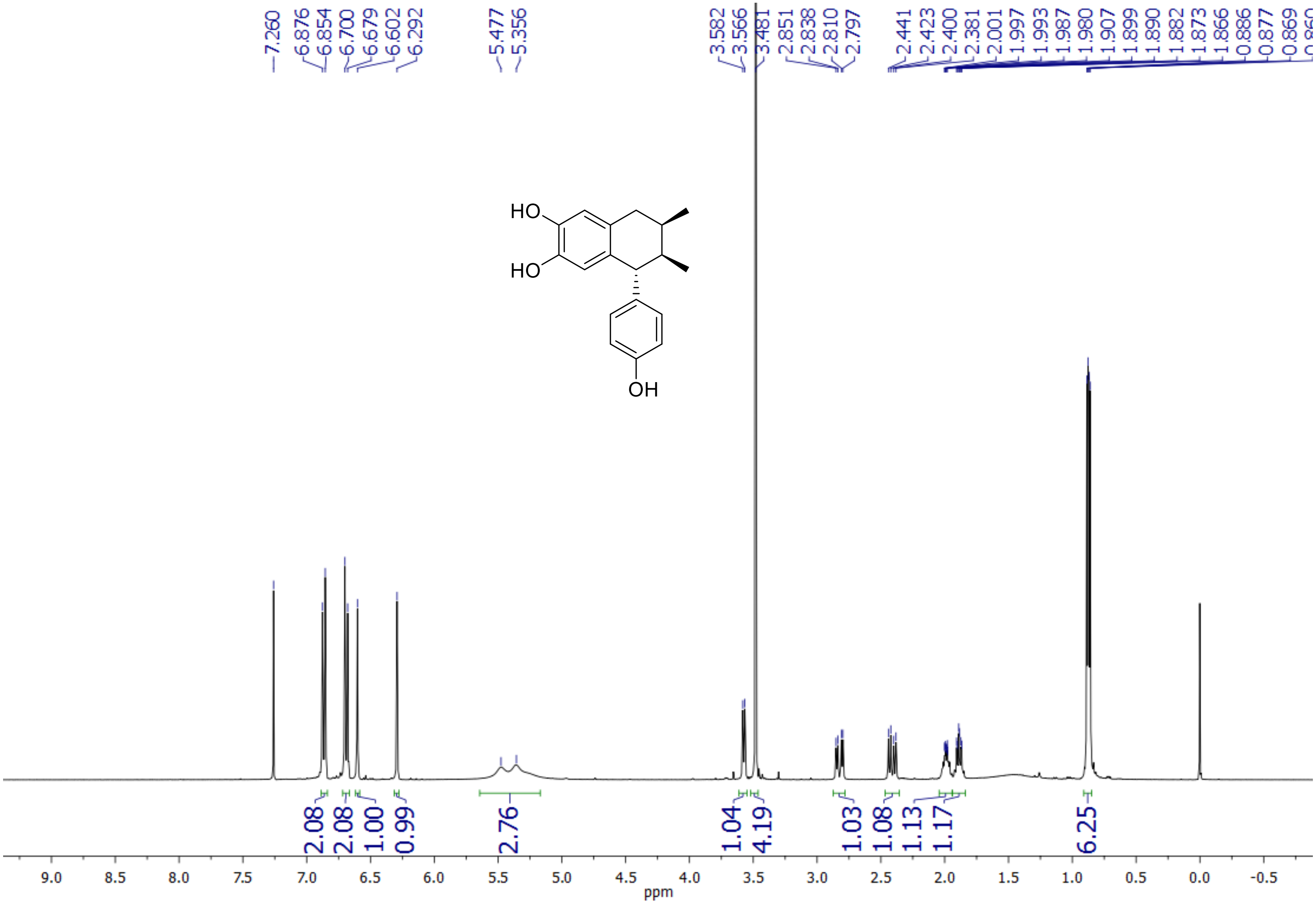

Figure S11. ^{13}C NMR spectrum of 3’-demethoxy-6-*O*-demethylisoguaiacin (**3**) in $(CD_3)_2CO$

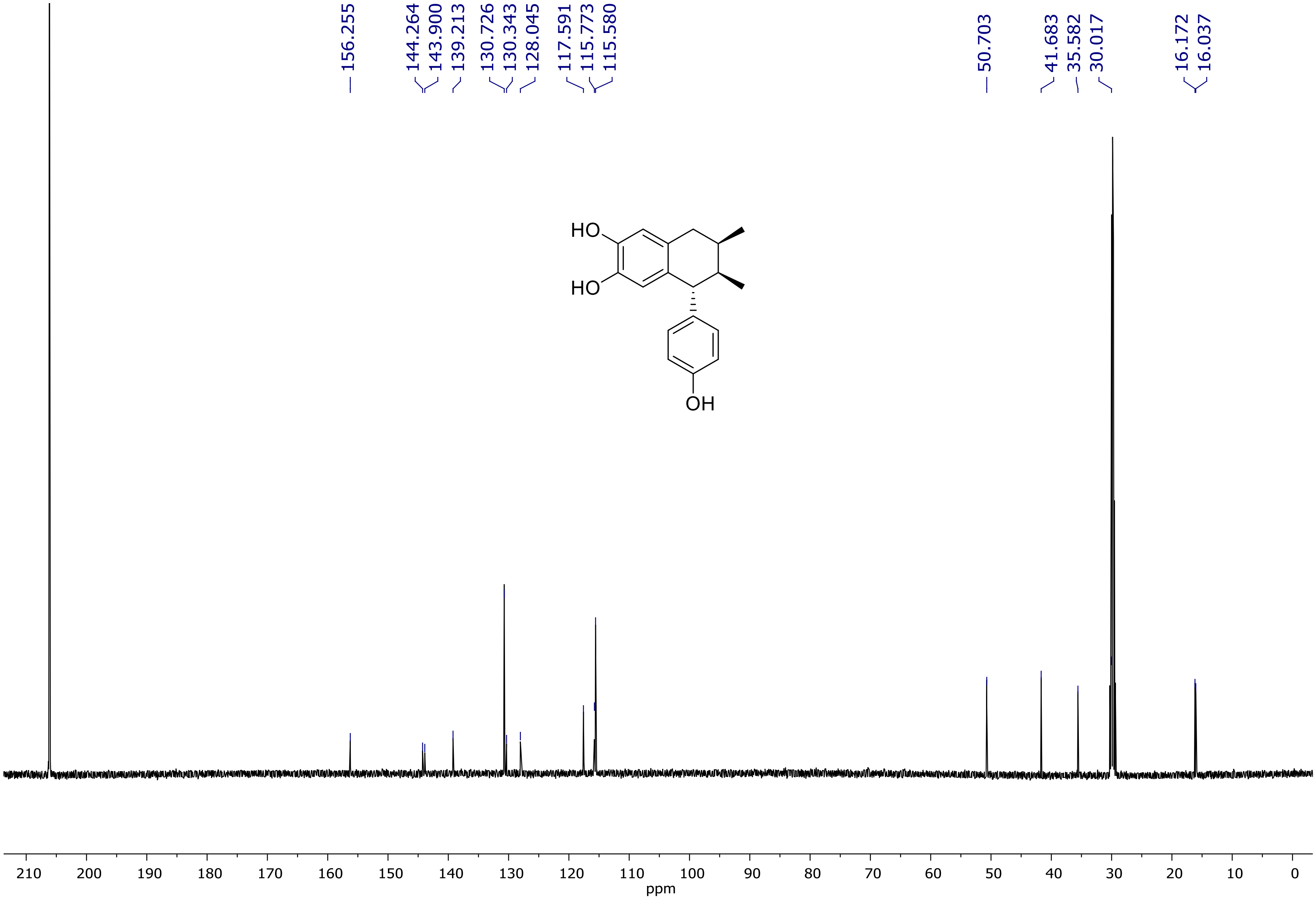

Figure S12. HMQC spectrum of 3'-demethoxy-6-*O*-demethylisoguaiacin (**3**) in $(CD_3)_2CO$

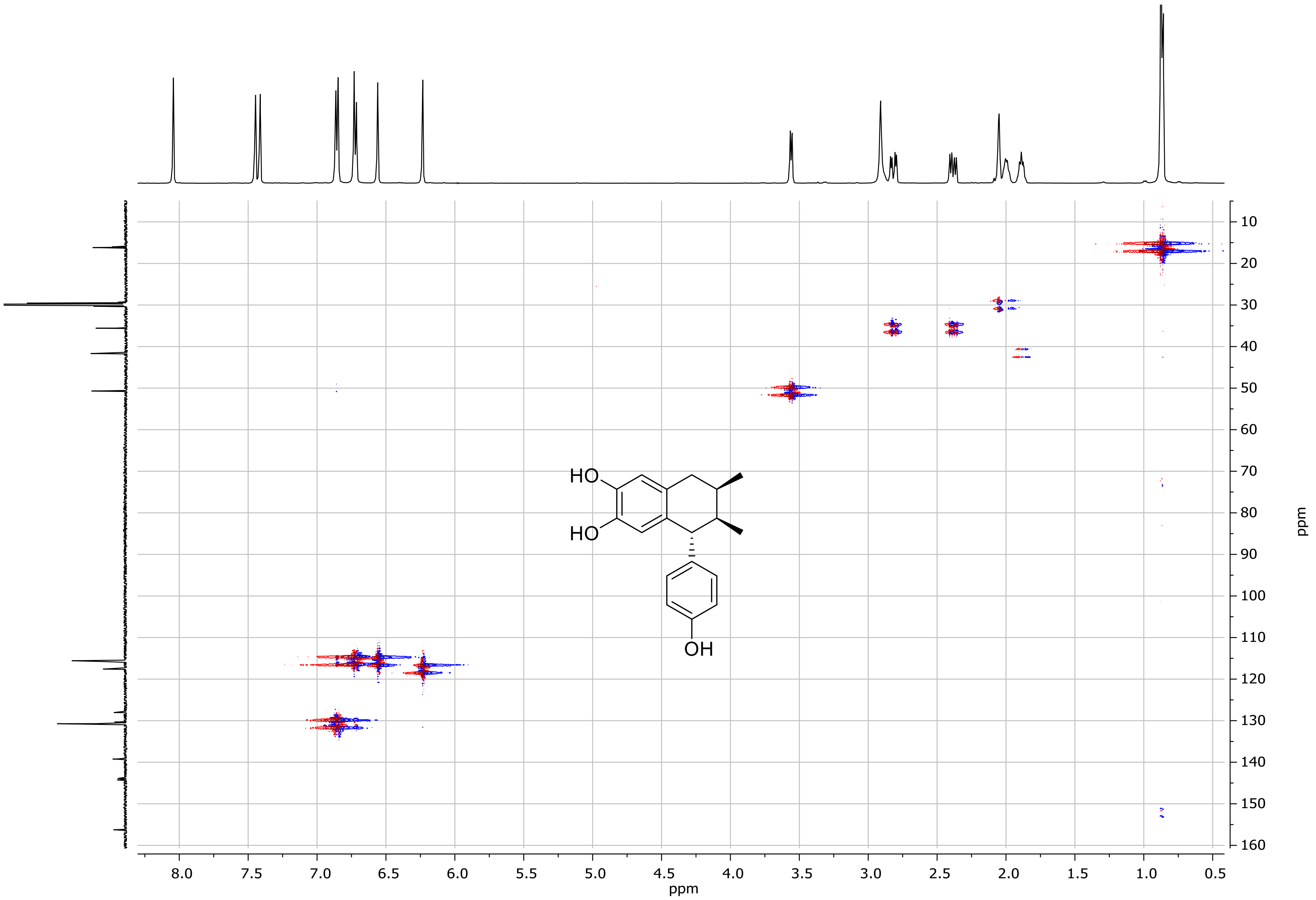

Figure S13. HMBC spectrum of 3'-demethoxy-6-*O*-demethylisoguaiacin (**3**) in $(CD_3)_2CO$

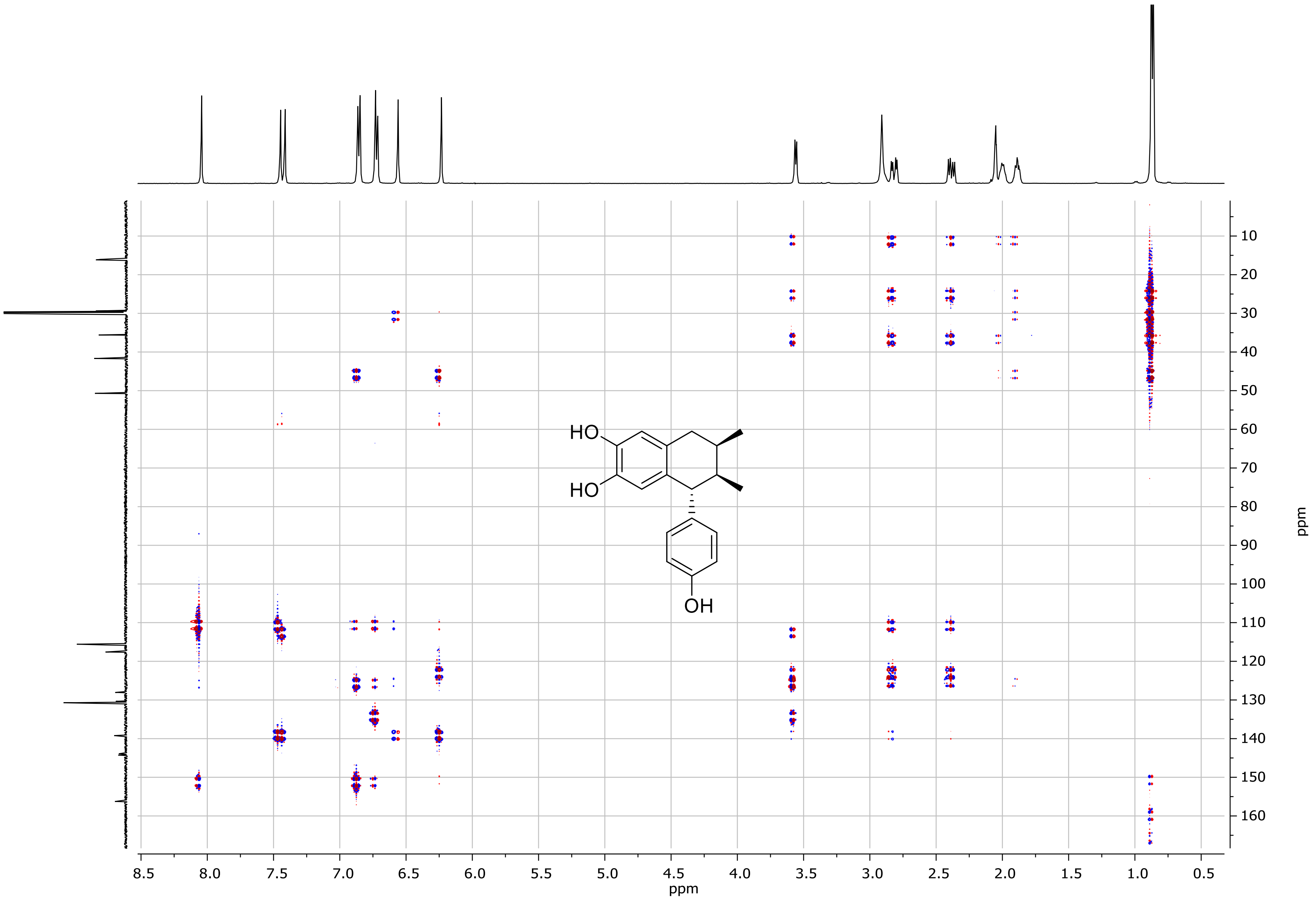

Figure S14. ^{1}H NMR spectrum of nor-isoguaicin (**4**) in $(CD_3)_2CO$

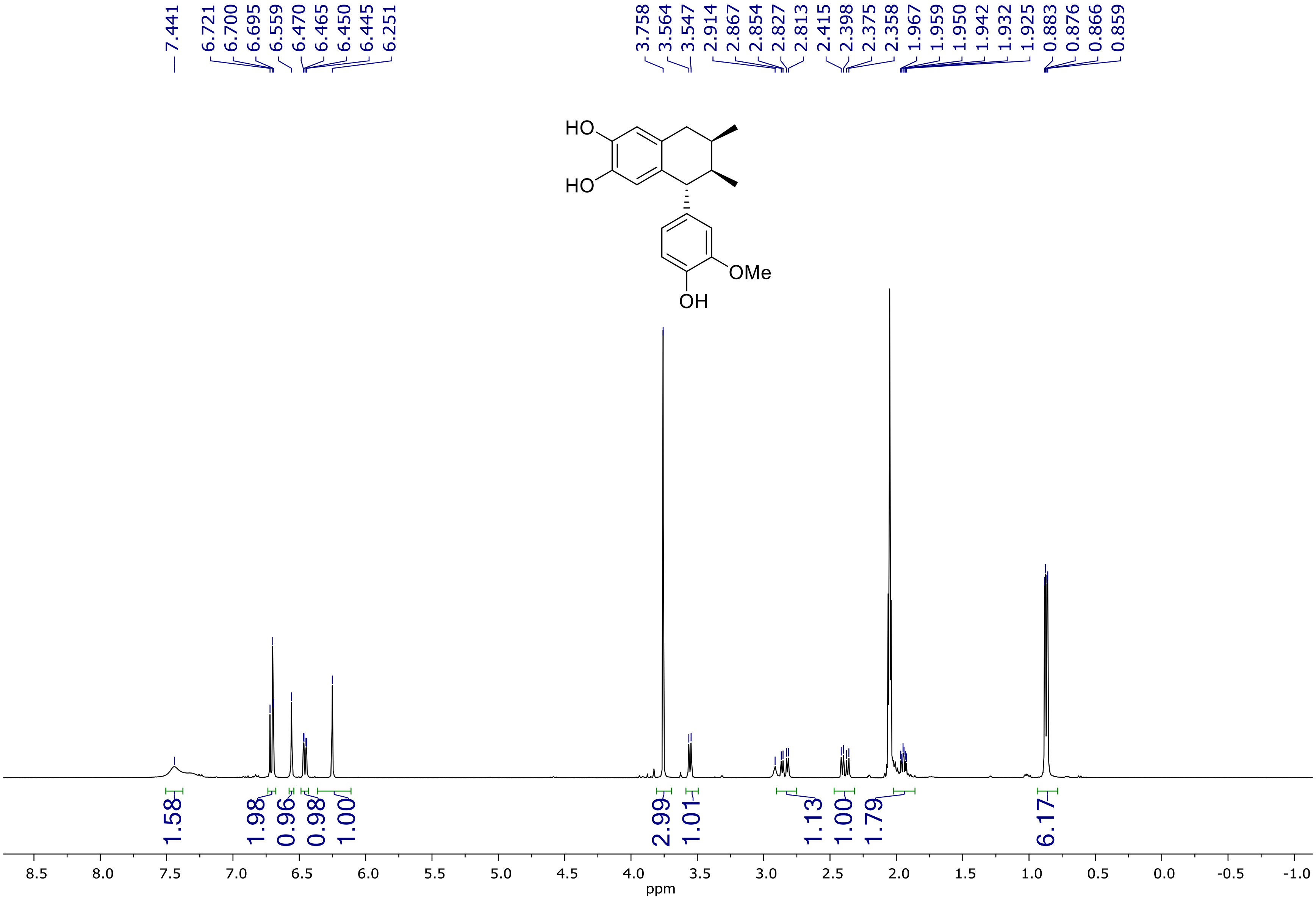

Figure S15. ^{1}H NMR spectrum of nor-isoguaicin (**4**) in $CDCl_3$

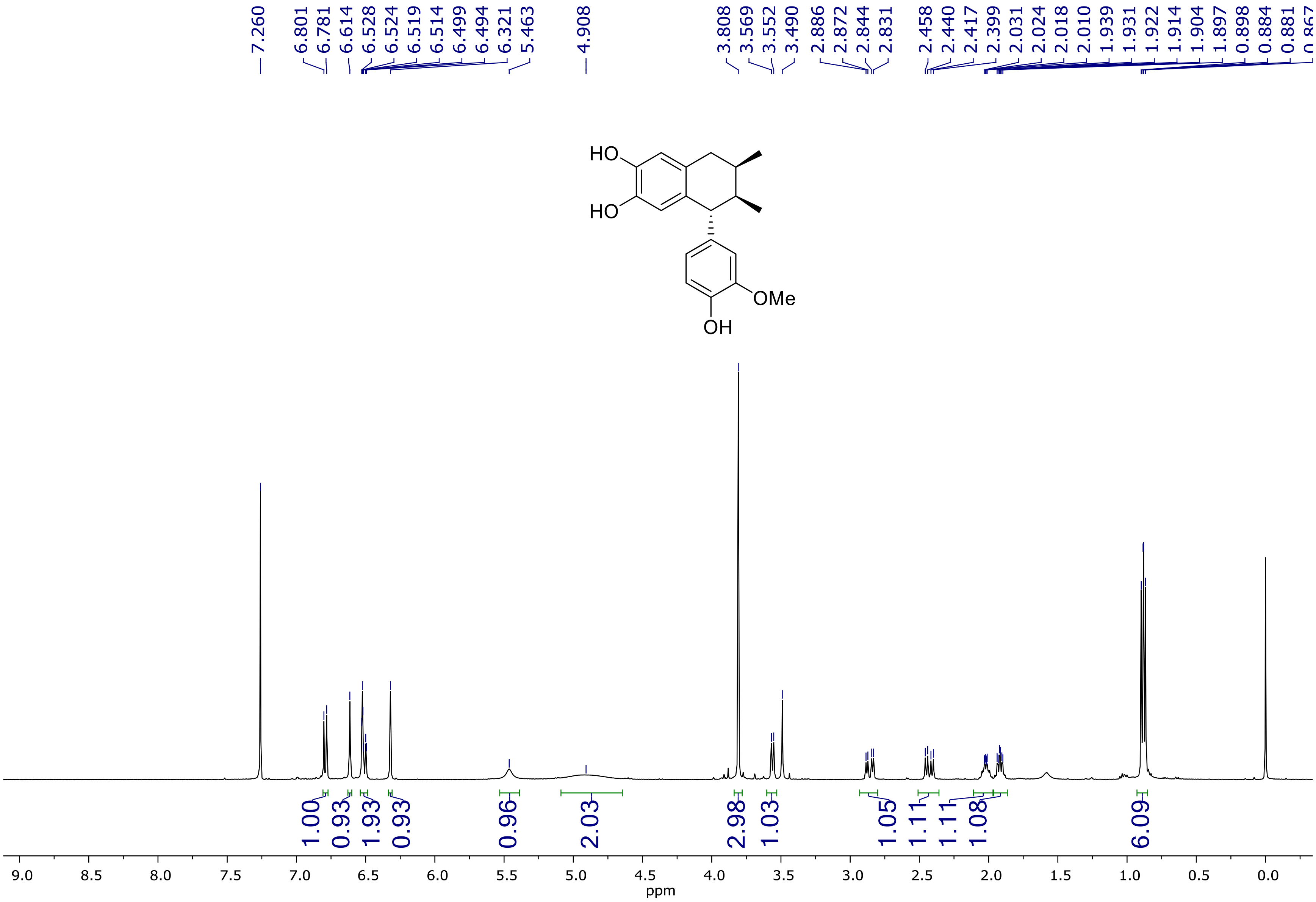

Figure S16. ^{13}C NMR spectrum of nor-isoguaicin (**4**) in $(CD_3)_2CO$

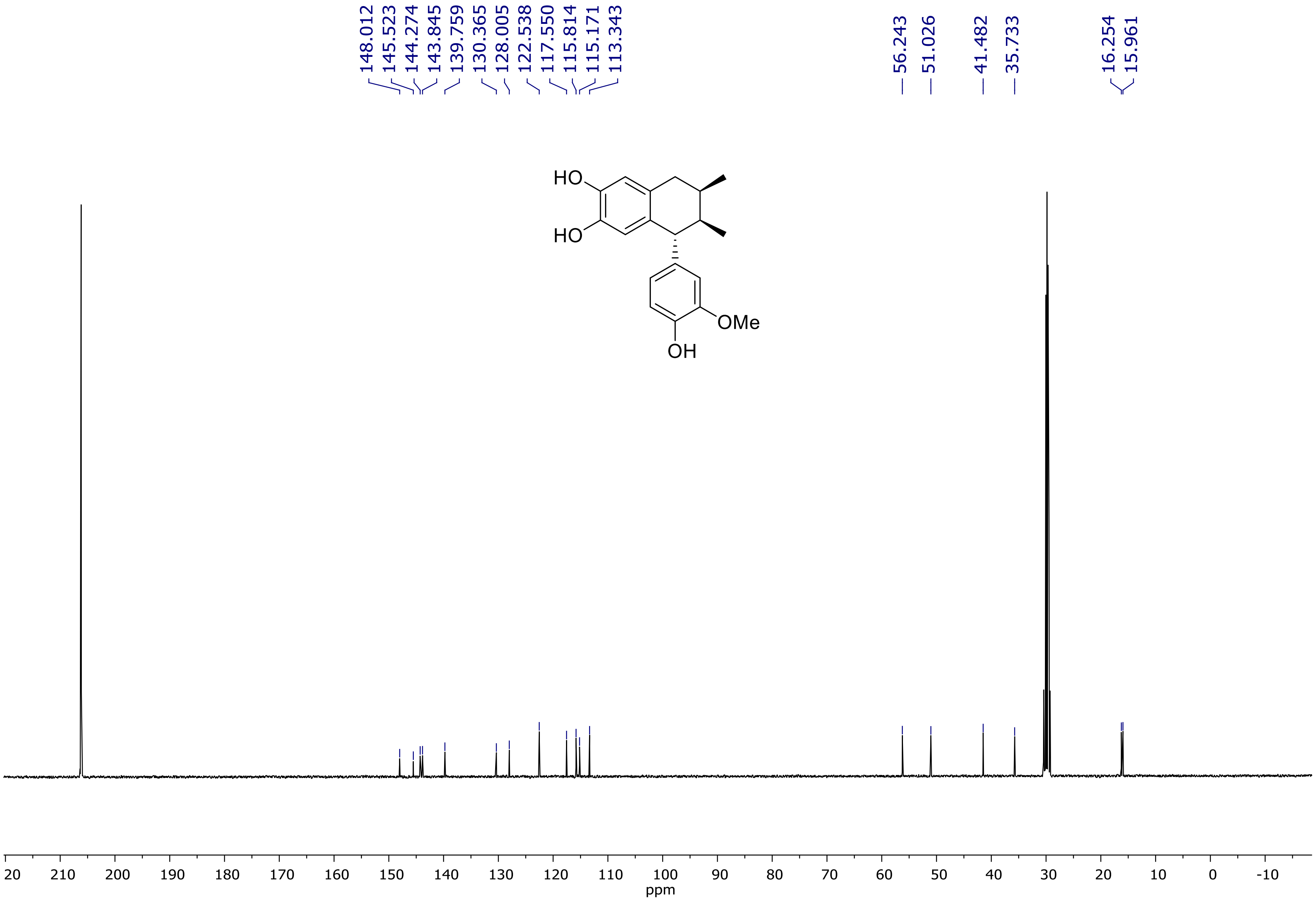

Figure S17. ^{13}C DEPT 135 NMR spectrum of nor-isoguaicin (**4**) in $(CD_3)_2CO$

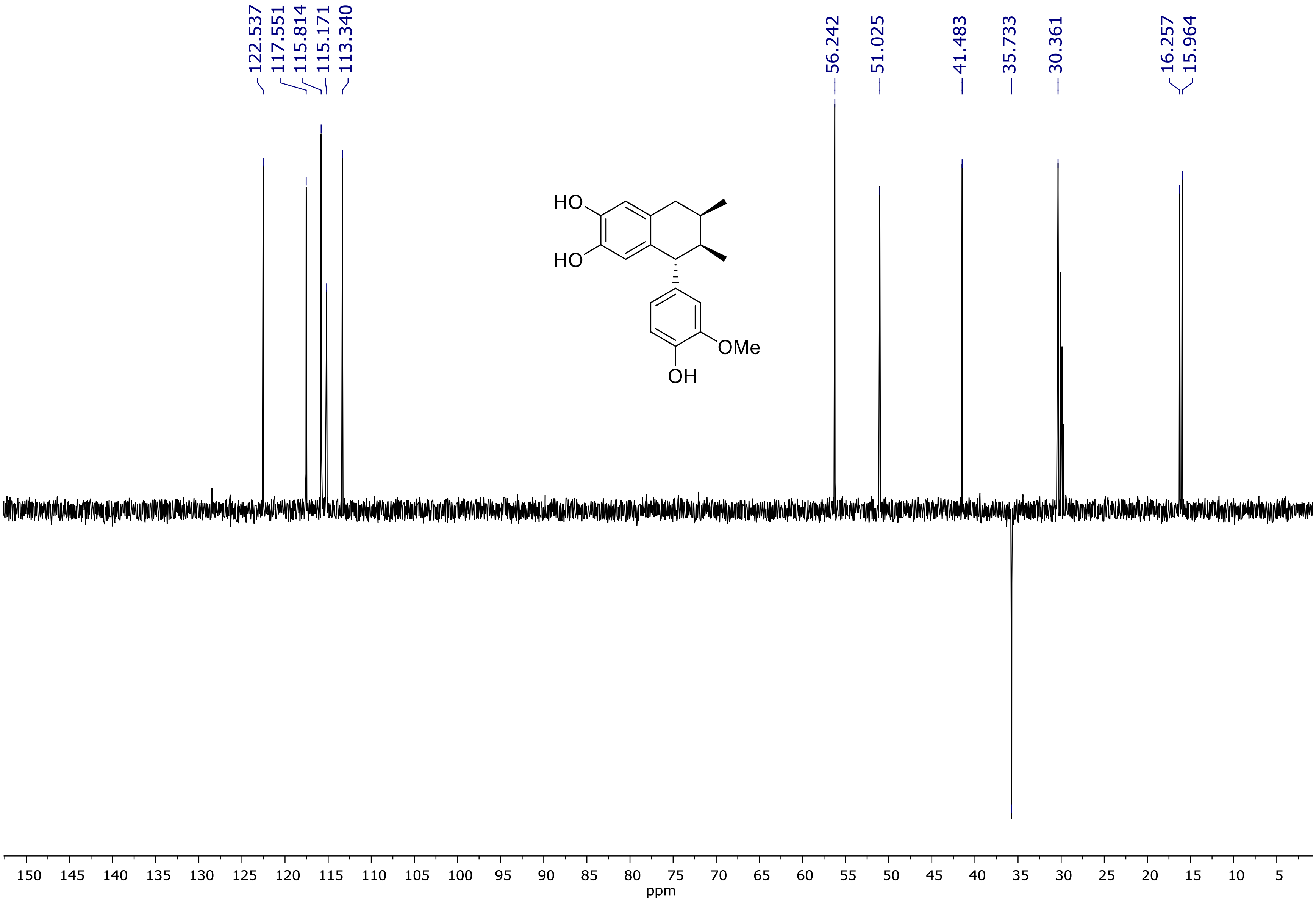

Figure S18. HMQC NMR spectrum of nor-isoguaicin (**4**) in $(CD_3)_2CO$

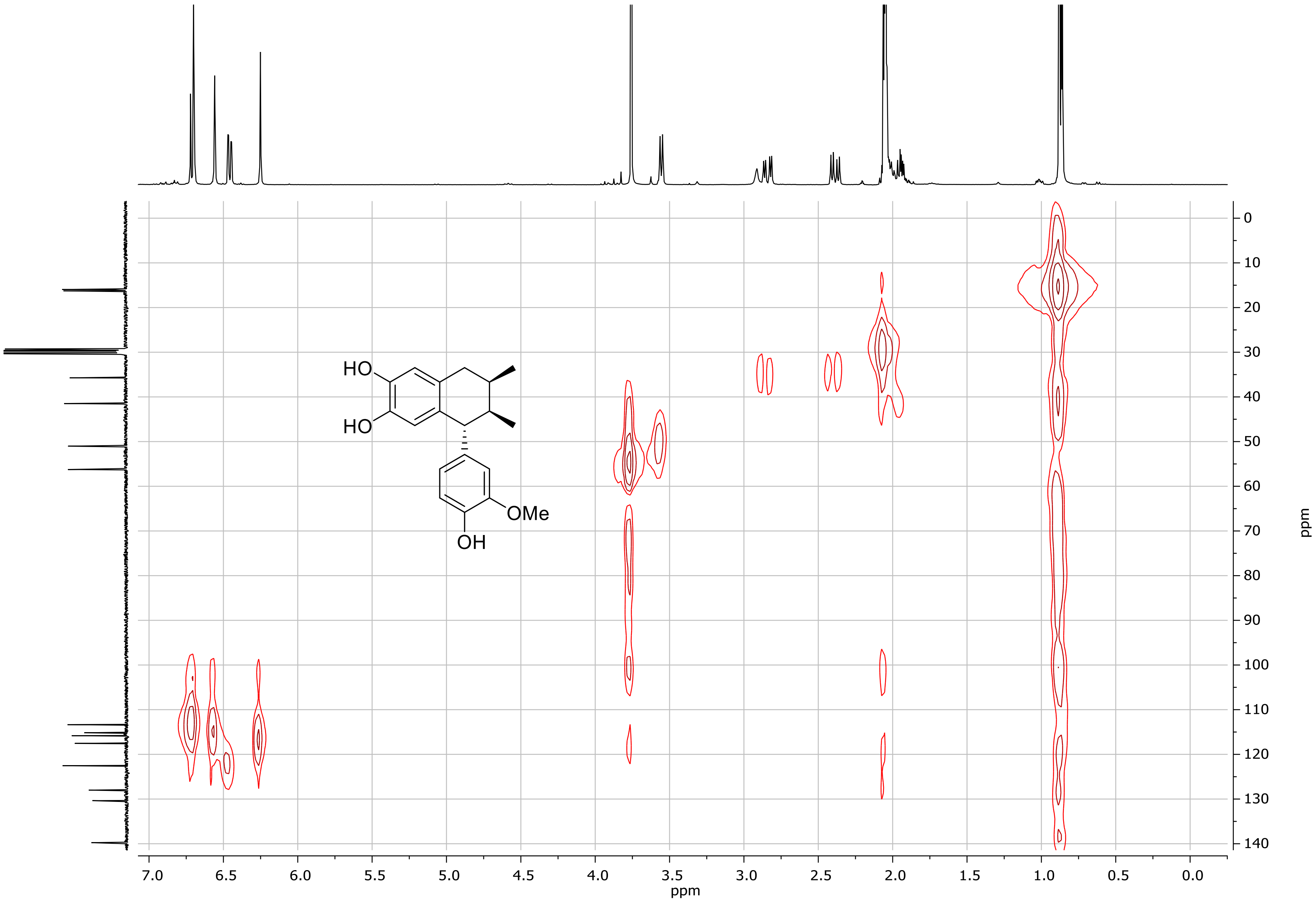

Figure S19. ^{1}H NMR spectrum of 3'-demethoxyisoguaiacin (**5**) in $(CD_3)_2CO$

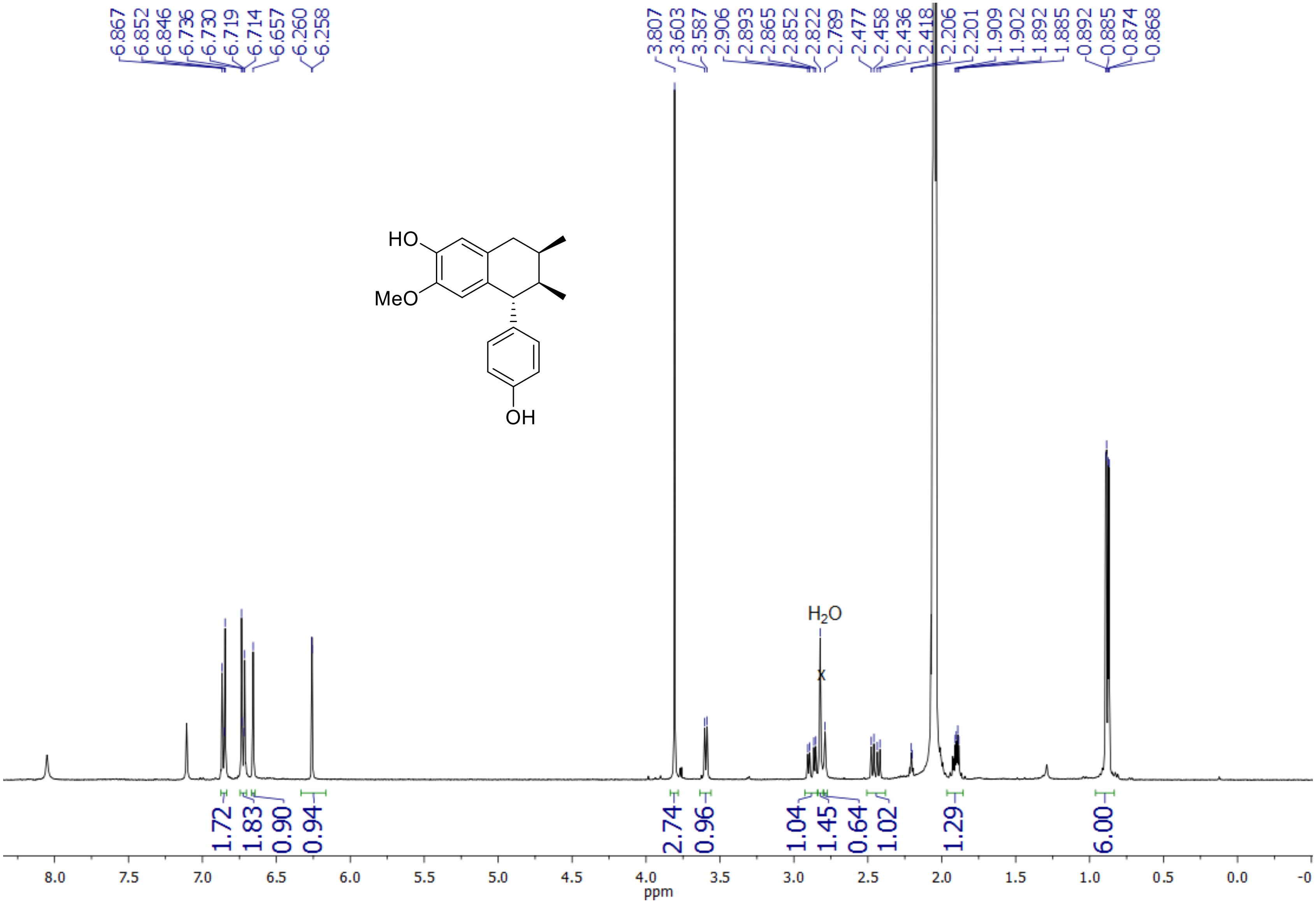

Figure S20. ^{13}C NMR spectrum of 3'-demethoxyisoguaiacin (**5**) in $(CD_3)_2CO$

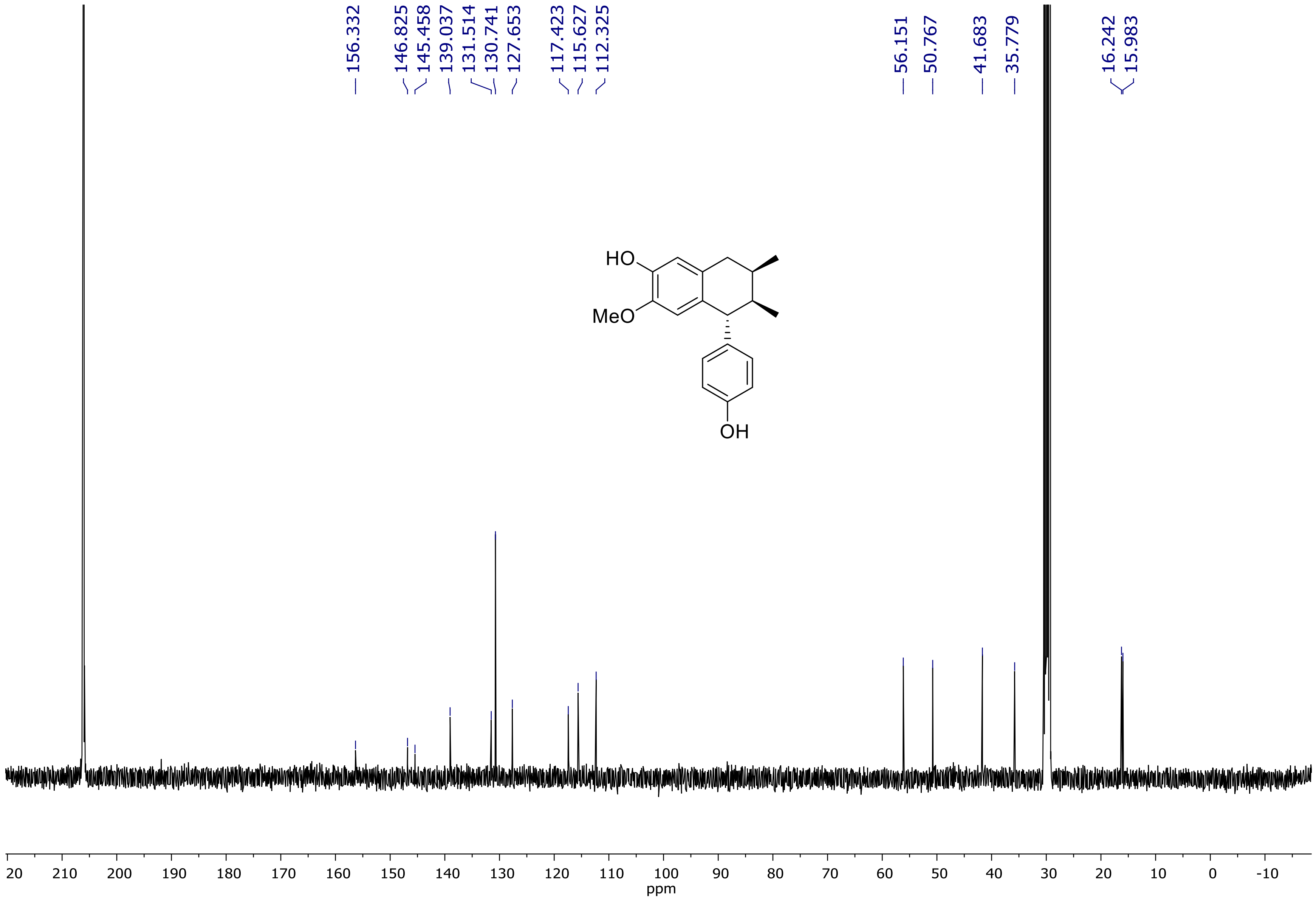

Figure S21. ^{13}C DEPT-135 NMR spectrum of 3'-demethoxyisoguaiacin (**5**) in $(CD_3)_2CO$

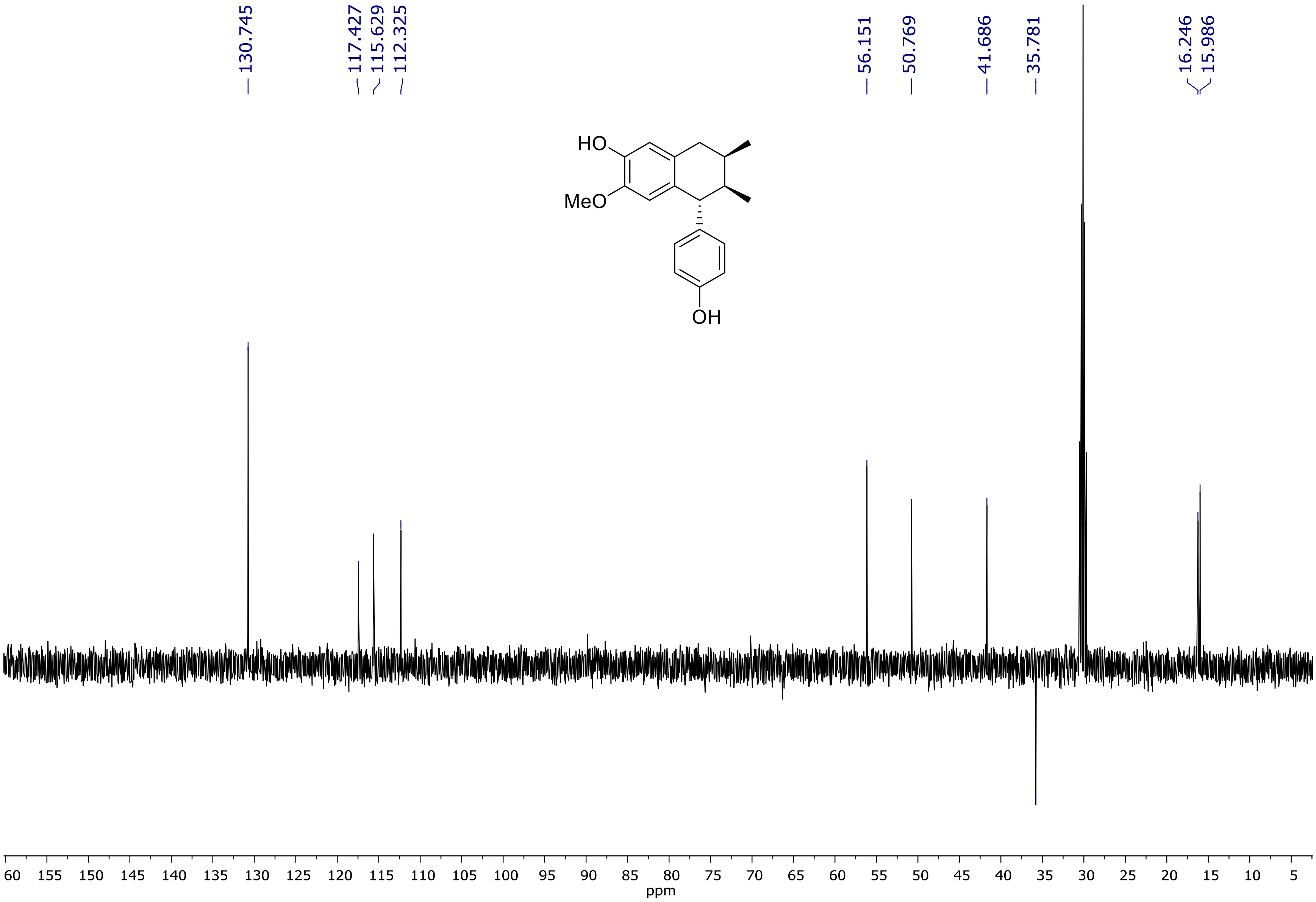

Figure S22. HMQC NMR spectrum of 3'-demethoxyisoguaiacin (**5**) in $(CD_3)_2CO$

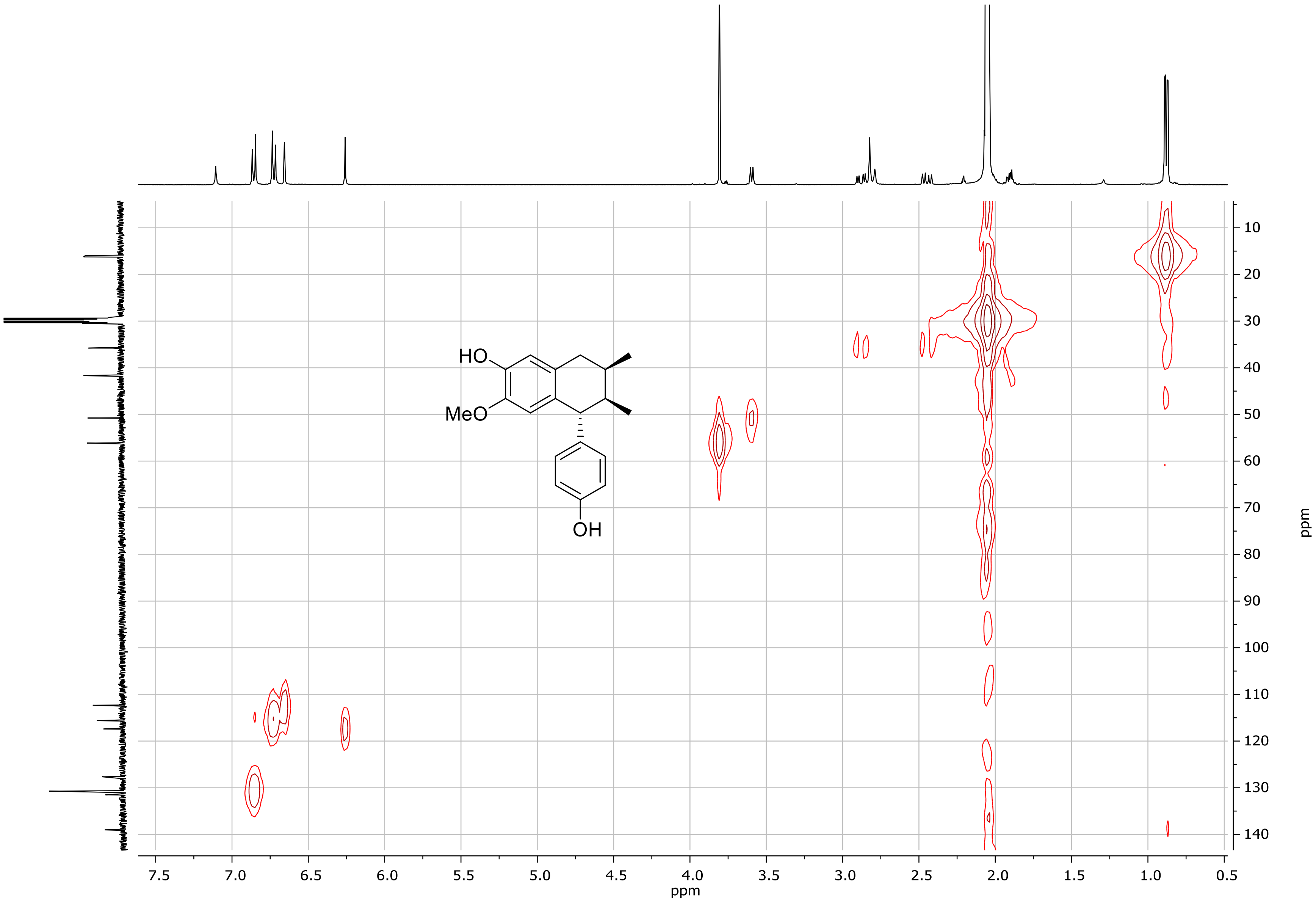

Figure S23. ^{1}H NMR spectrum of 6, 3'-di-*O*-demethoxy-isoguaiacin (**6**) in CD_3OD

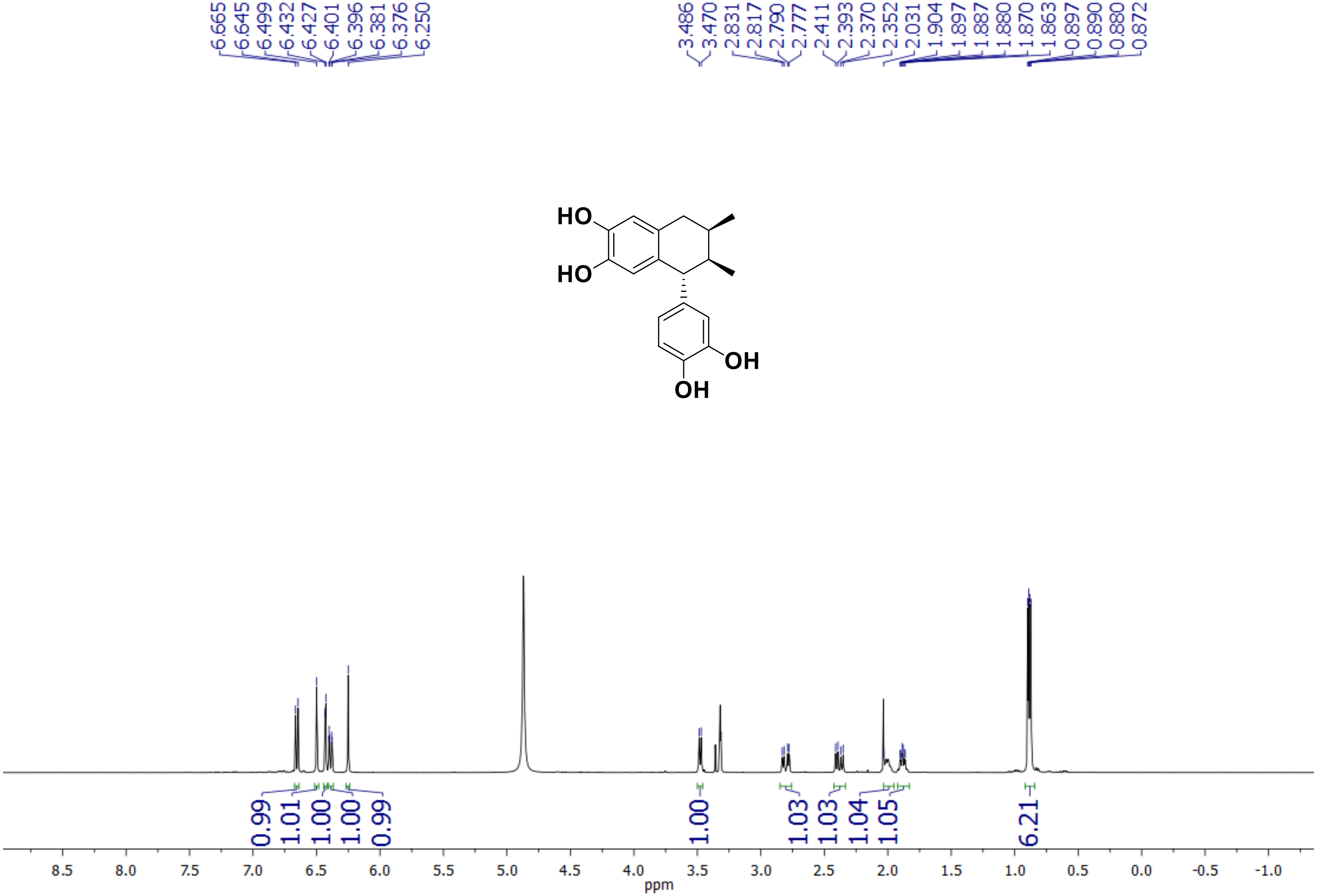

Figure S24. ^{13}C NMR spectrum of 6, 3'-di-*O*-demethoxy-isoguaiacin (**6**) in CD_3OD

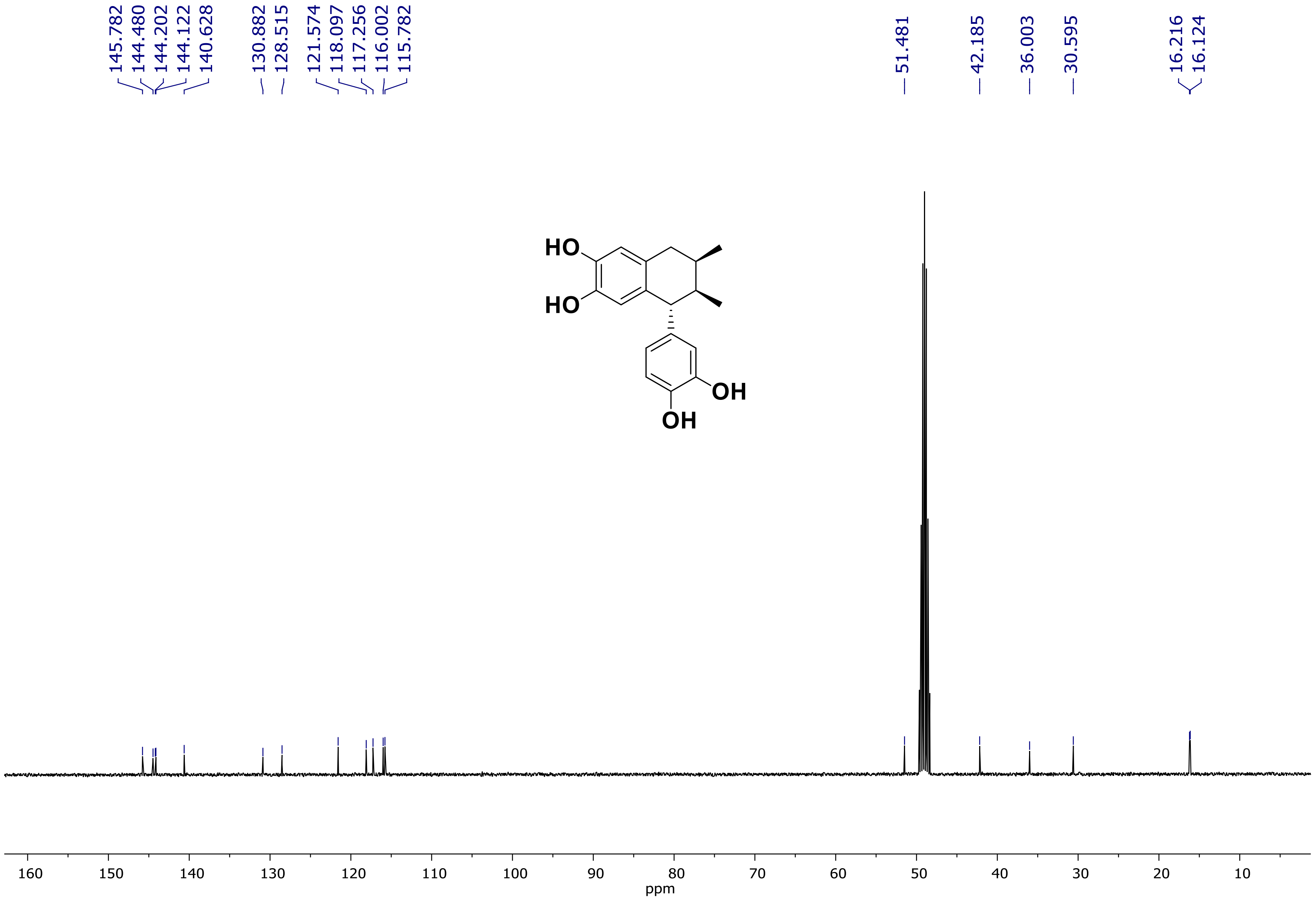

Figure S25. ^{13}C DEPT-135 NMR spectrum of 6, 3'-di-*O*-demethoxy-isoguaiacin (**6**) in CD_3OD

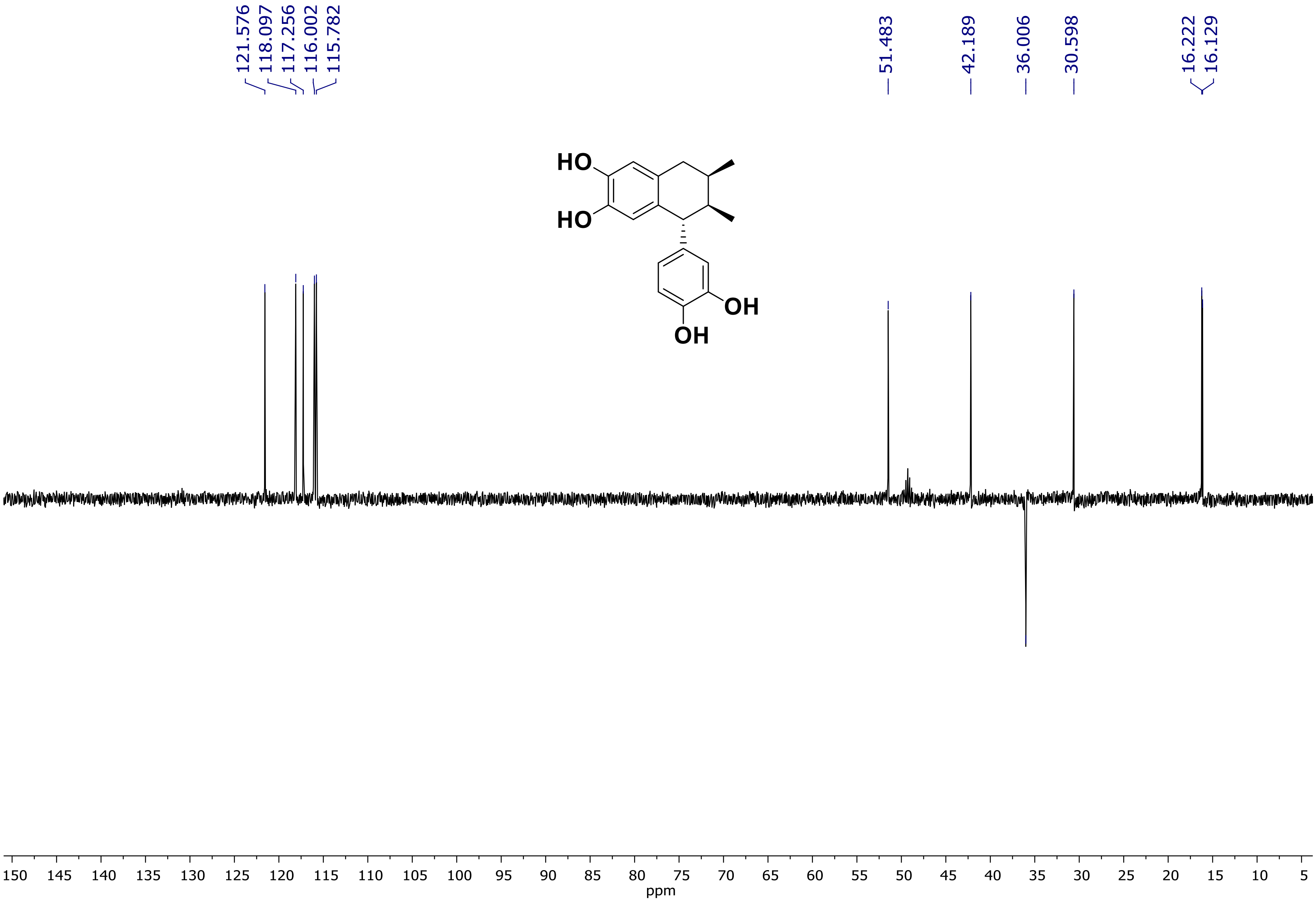

Figure S26. ^{1}H NMR spectrum of 3'-hydroxy-4-epi-larreatricin (**7**) in $(CD_3)_2CO$

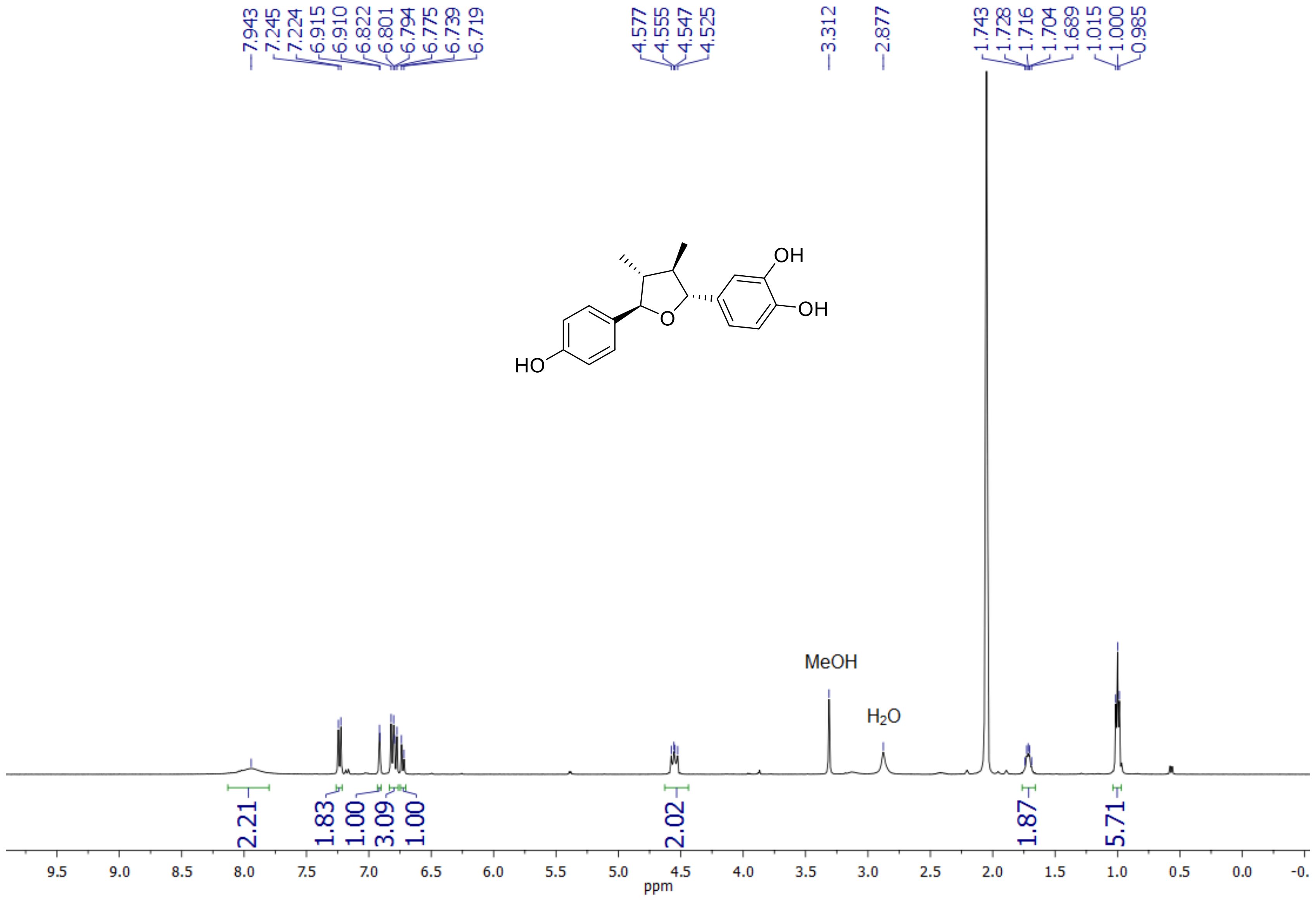

Figure S27. ^{13}C NMR spectrum of 3'-hydroxy-4-epi-larreatricin (**7**) in $(CD_3)_2CO$

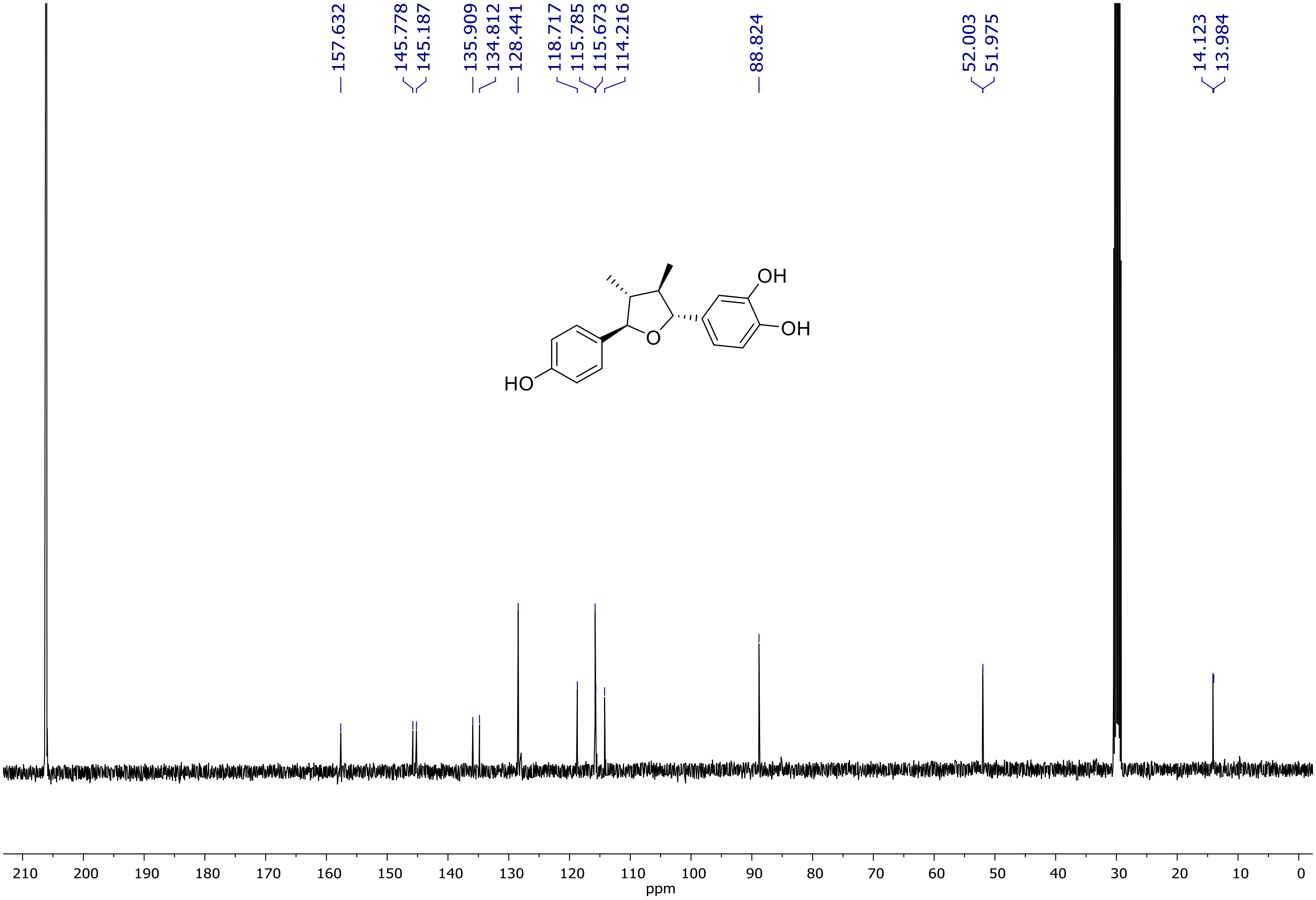

Figure S28. ^{13}C DEPT-135 NMR spectrum of 3'-hydroxy-4-epi-larreatricin (**7**) in $(CD_3)_2CO$

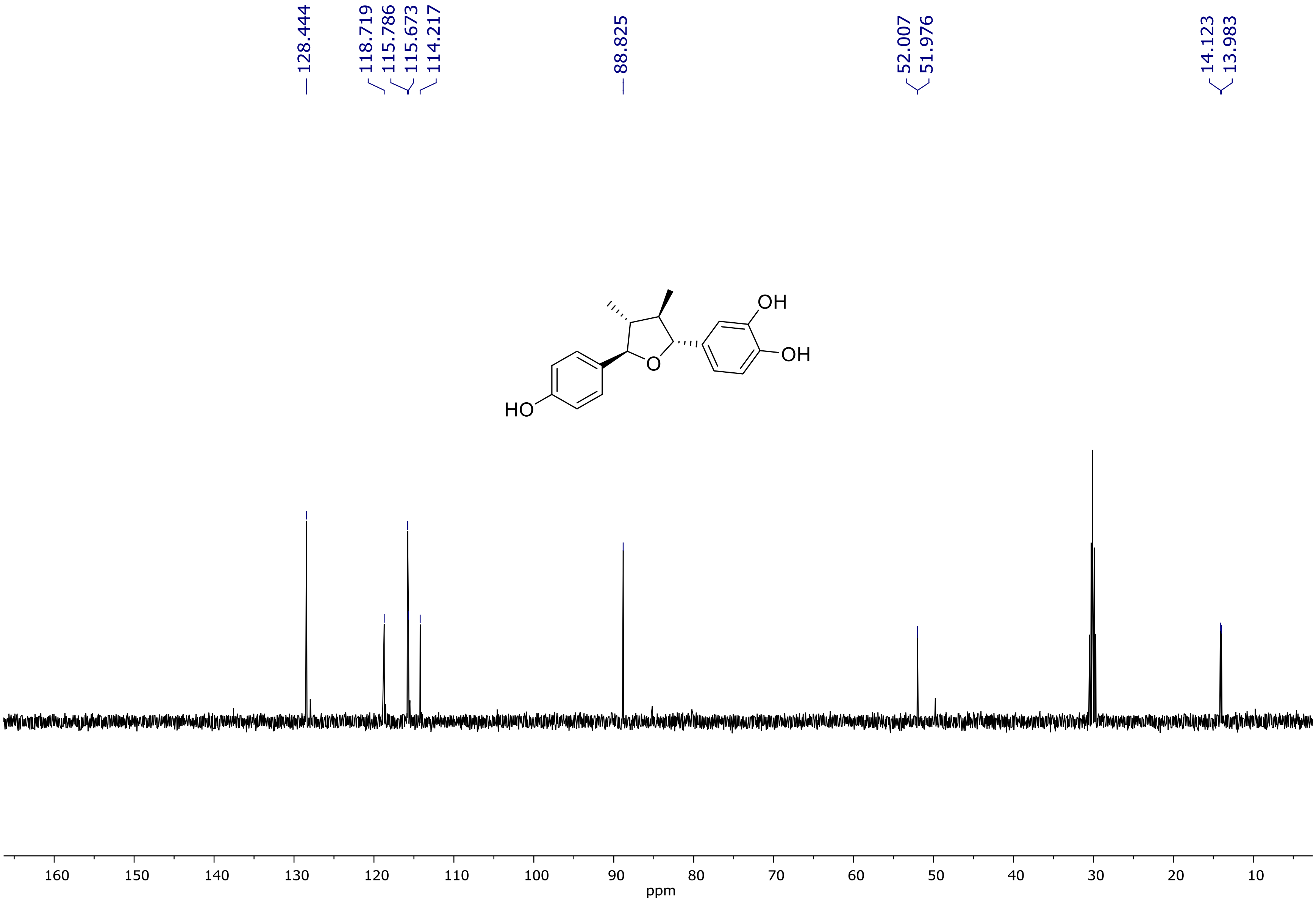

Figure S29. ^{13}C DEPT-90 NMR spectrum of 3’-hydroxy-4-epi-larreatricin (**7**) in $(CD_3)_2CO$

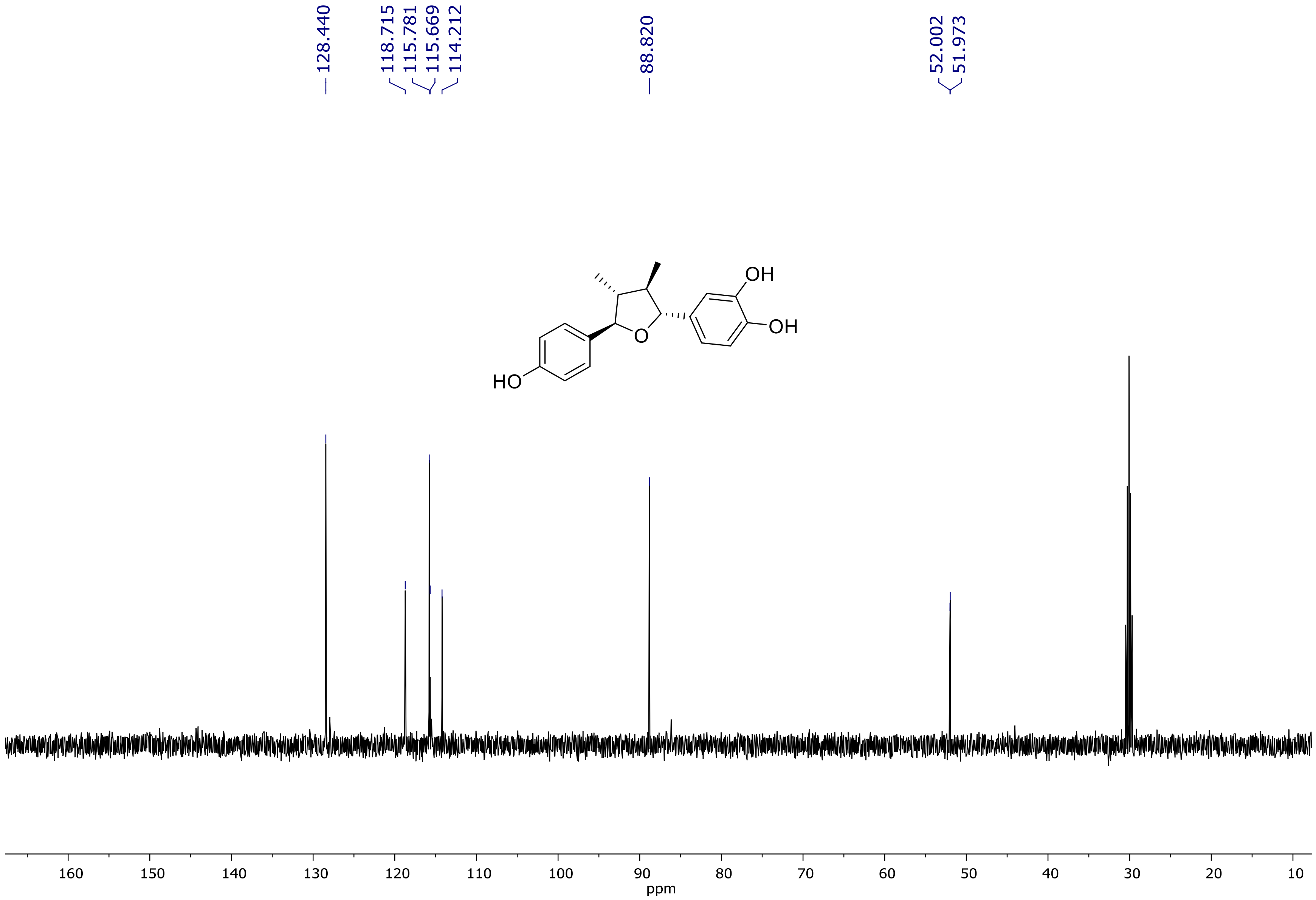

Figure S30. HMQC NMR spectrum of 3'-hydroxy-4-epi-larreatricin (**7**) in $(CD_3)_2CO$

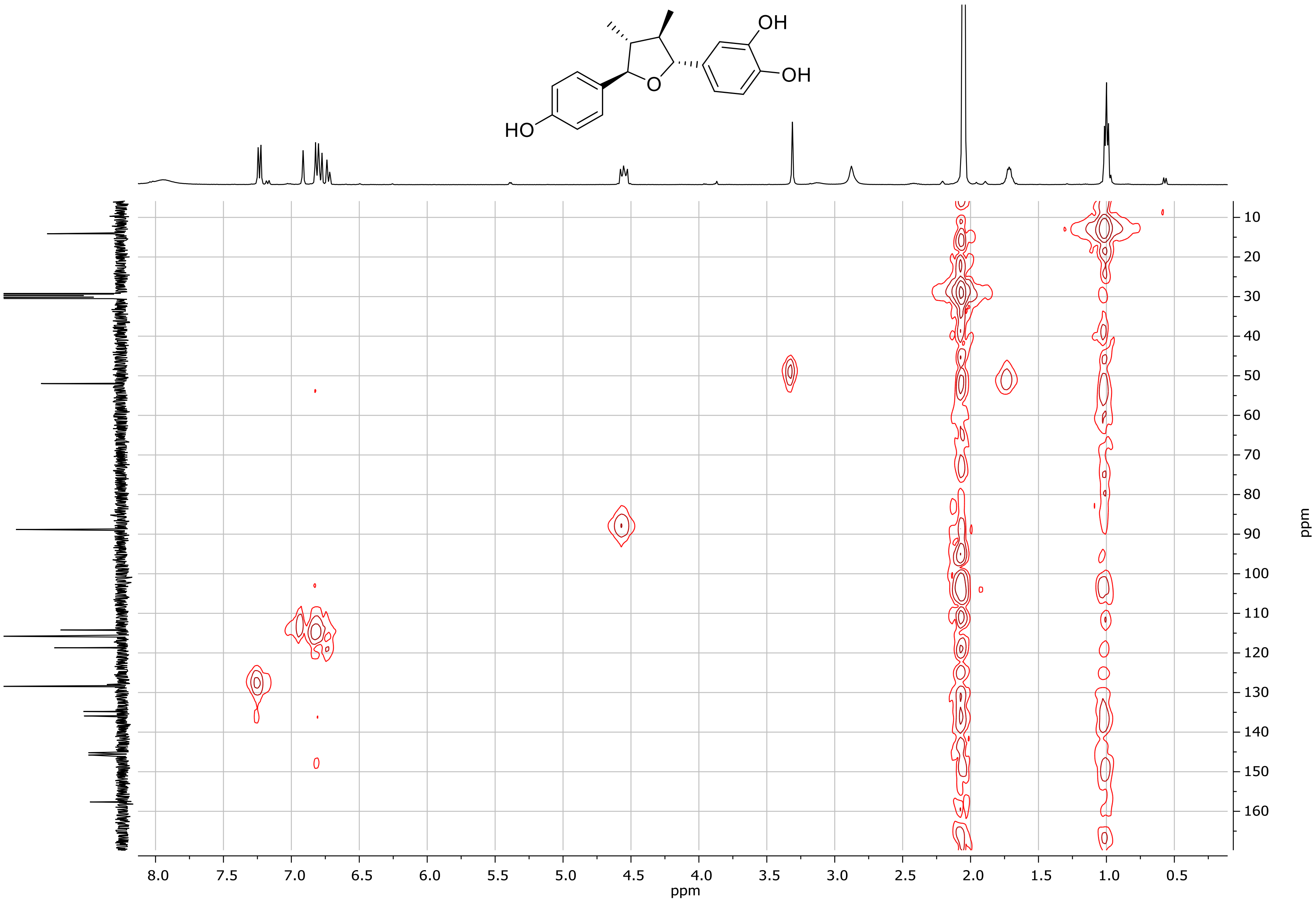

Figure S31. ^{1}H NMR spectrum of (7R, 7'R)-7, 7'-bis(4', 3, 4-trihydroxyphenyl)-(8R, 8'S)-8, 8'-dimethyltetrahydrofuran (**8**) in $(CD_3)_2CO$

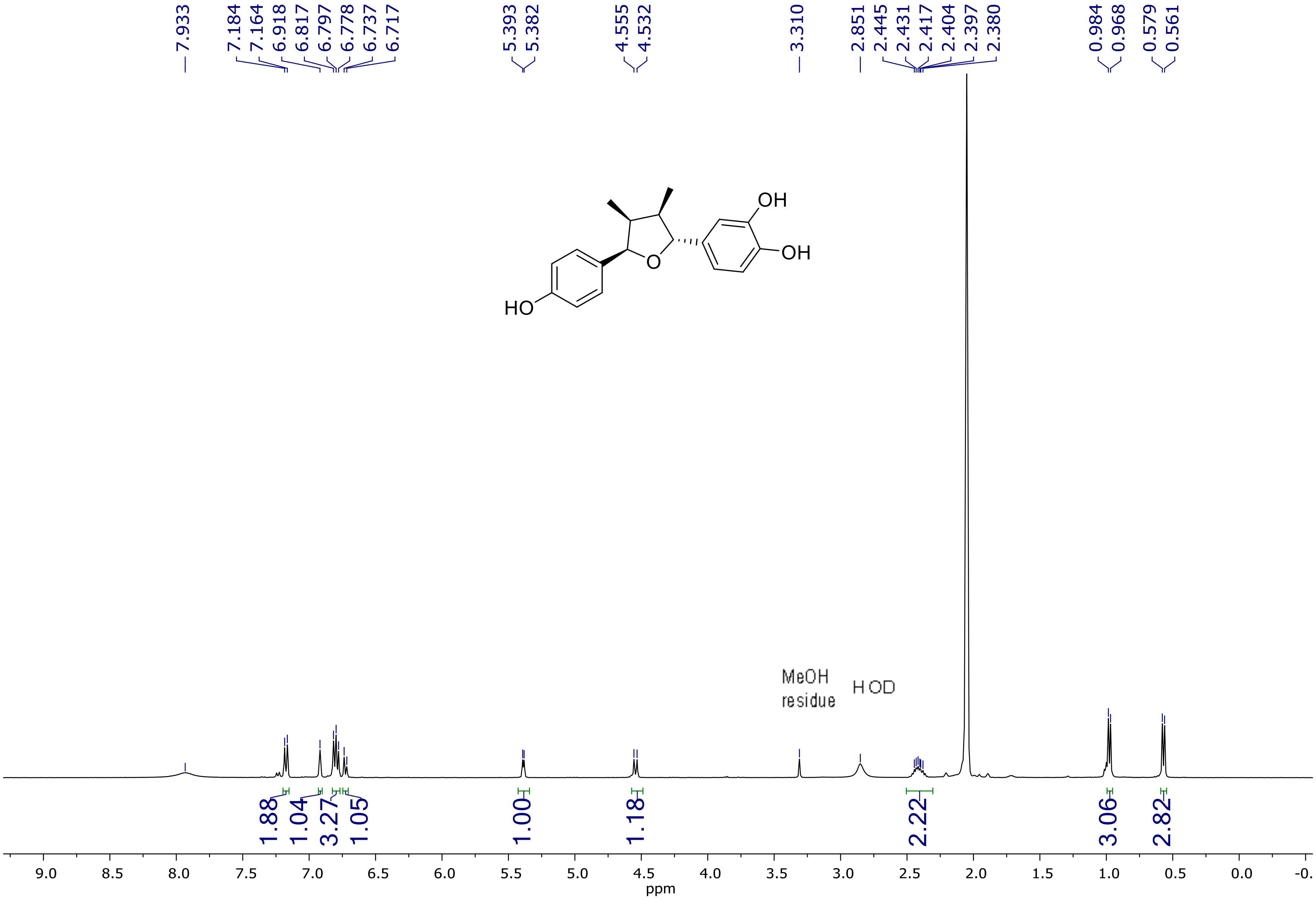

Figure S32. ^{13}C NMR spectrum of of (7R, 7'R)-7, 7'-bis(4', 3, 4-trihydroxyphenyl)-(8R, 8'S)-8, 8'-dimethyltetrahydrofuran (**8**) in $(CD_3)_2CO$

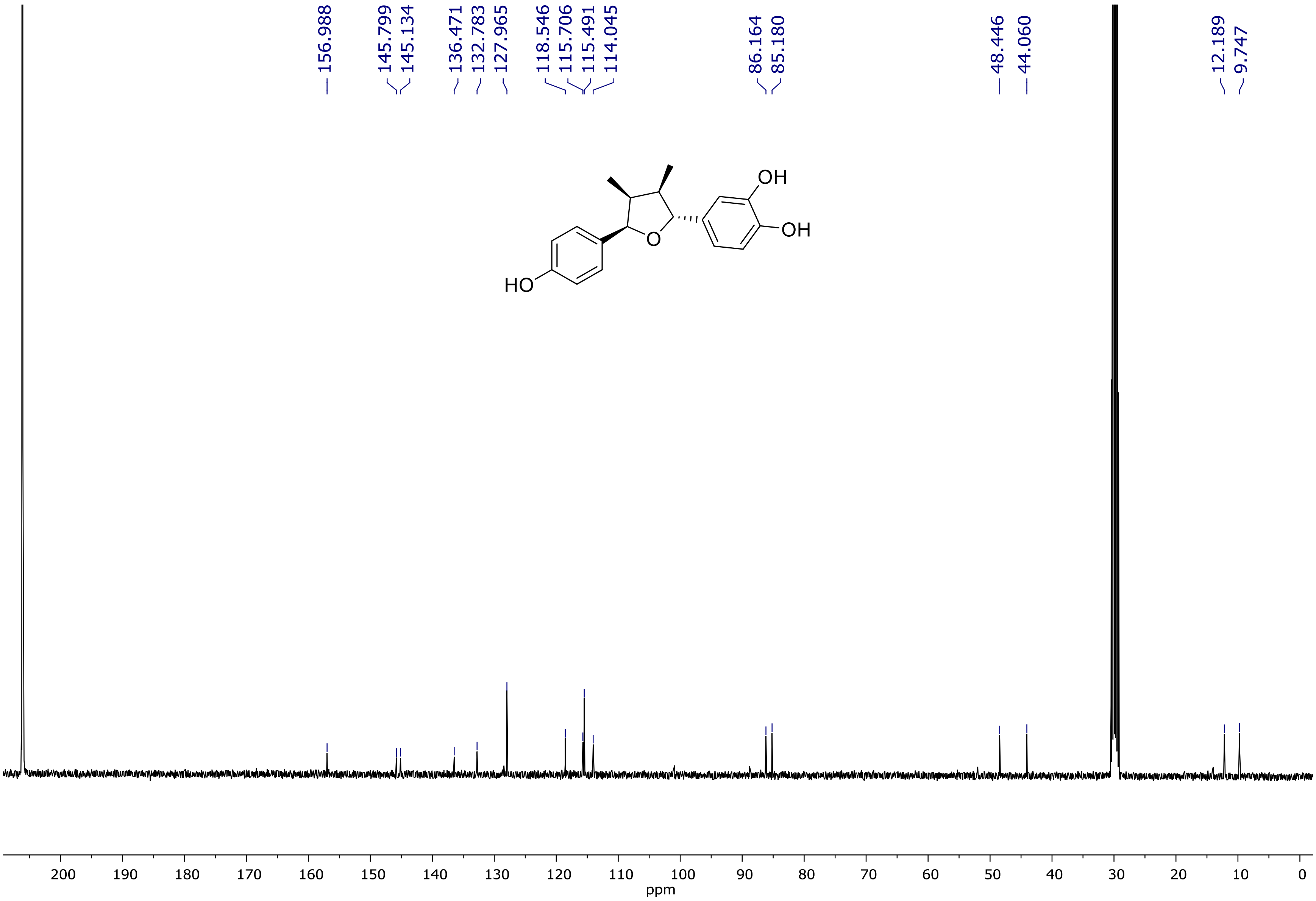

Figure S33. ^{13}C DEPT-135 NMR spectrum of of (7R, 7'R)-7, 7'-bis(4', 3, 4-trihydroxyphenyl)-(8R, 8'S)-8, 8'-dimethyltetrahydrofuran (**8**) in $(CD_3)_2CO$

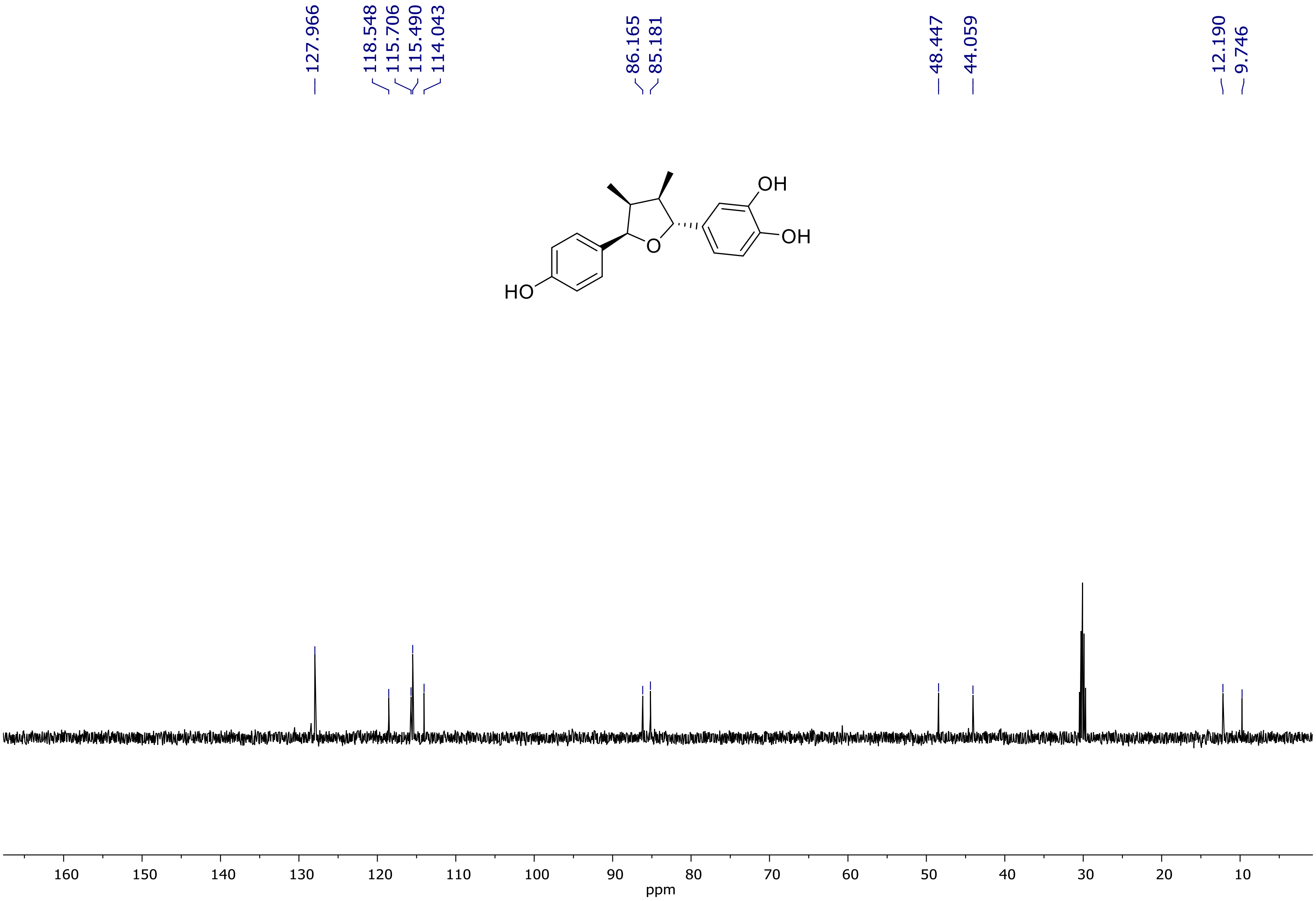

Figure S34. ^{13}C DEPT-90 NMR spectrum of of (7R, 7'R)-7, 7'-bis(4', 3, 4-trihydroxyphenyl)-(8R, 8'S)-8, 8'-dimethyltetrahydrofuran (**8**) in $(CD_3)_2CO$

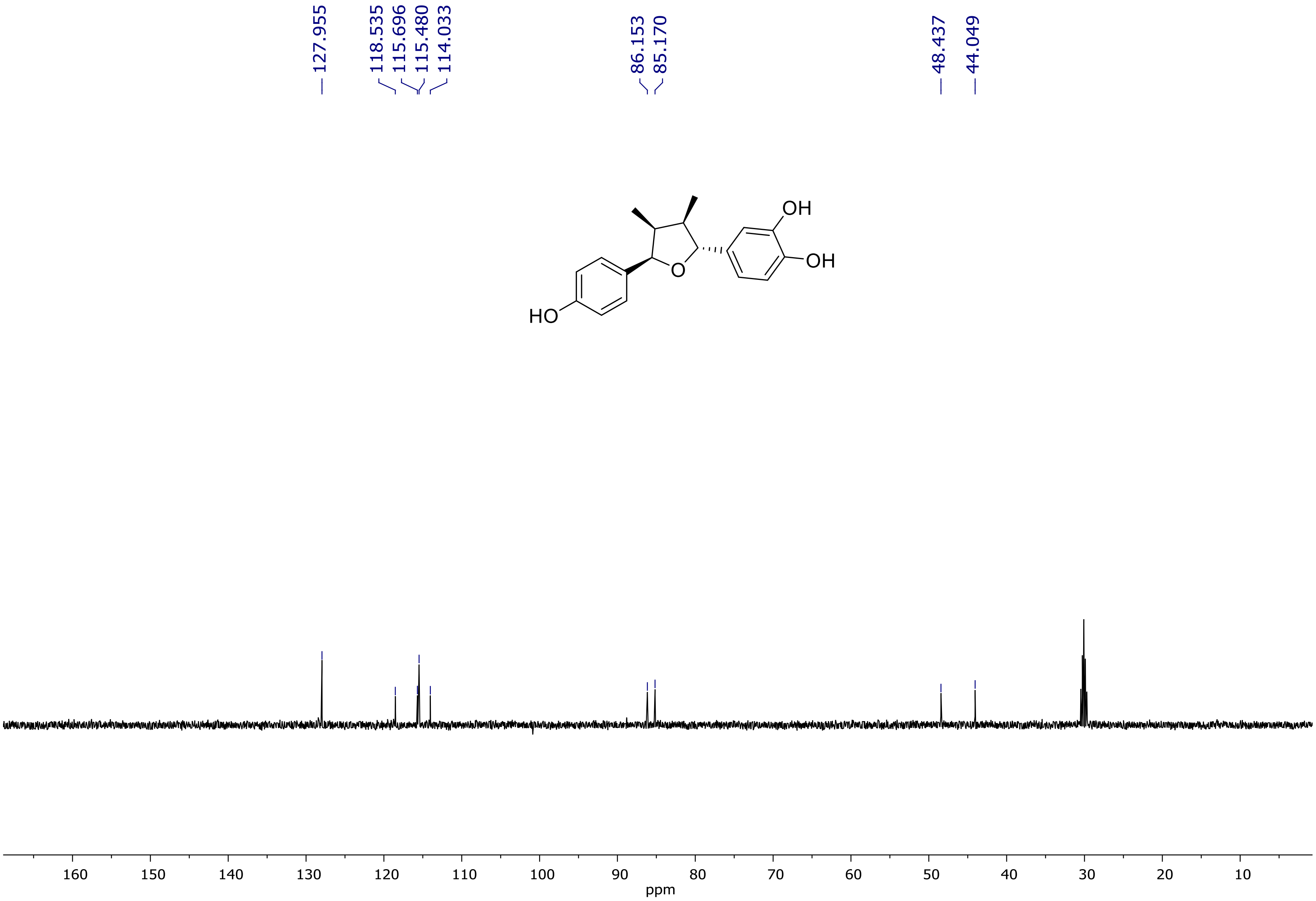

Figure S35. HMQC NMR spectrum of (7R, 7'R)-7, 7'-bis(4', 3, 4-trihydroxyphenyl)-(8R, 8'S)-8, 8'-dimethyltetrahydrofuran (**8**) in $(CD_3)_2CO$

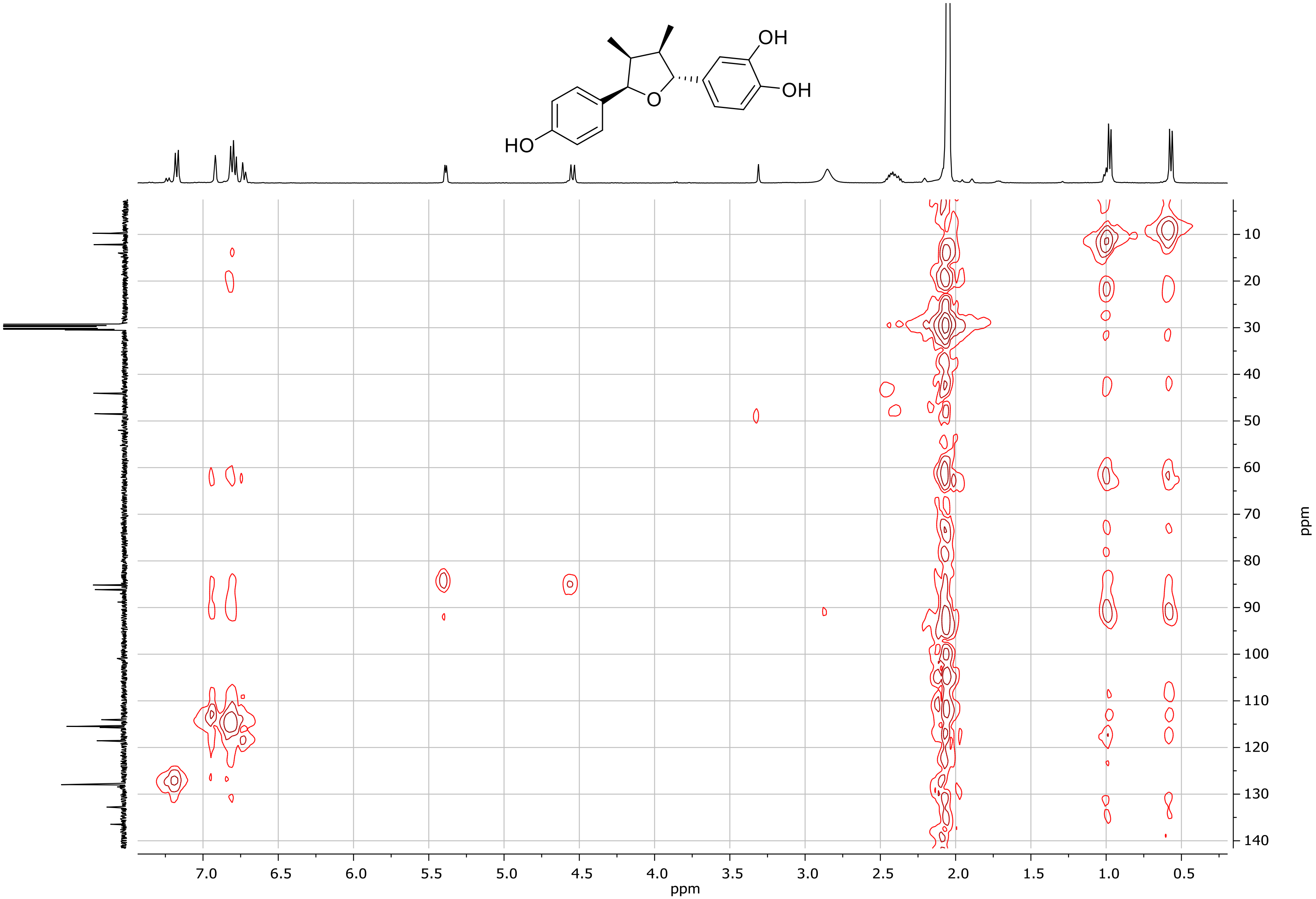

Figure S36. HMBC NMR spectrum of of (7R, 7'R)-7, 7'-bis(4', 3, 4-trihydroxyphenyl)-(8R, 8'S)-8, 8'-dimethyltetrahydrofuran (**8**) in $(CD_3)_2CO$

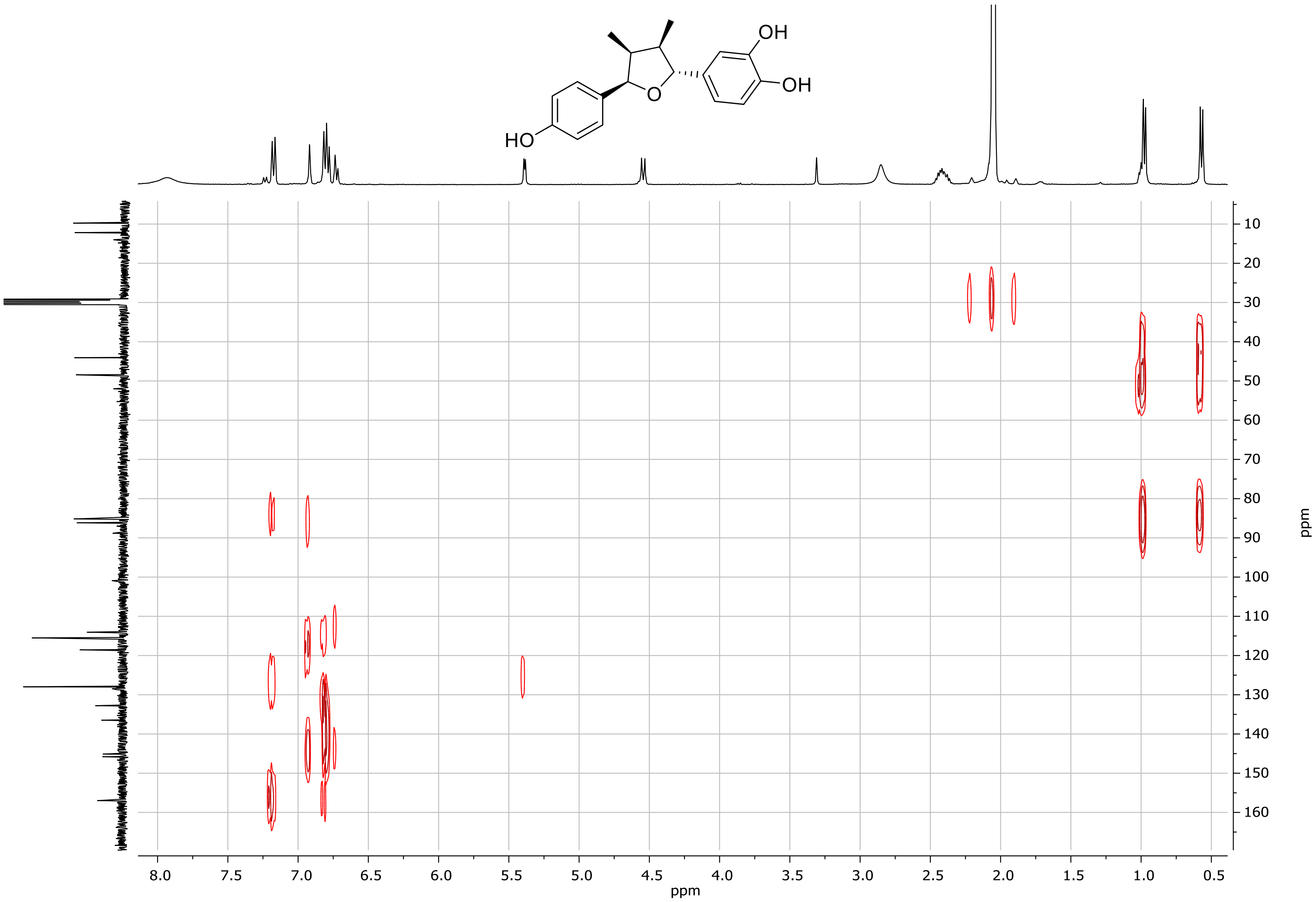

Figure S37. NOESY NMR spectrum of of (7R, 7'R)-7, 7'-bis(4', 3, 4-trihydroxyphenyl)-(8R, 8'S)-8, 8'-dimethyltetrahydrofuran (**8**) in $(CD_3)_2CO$

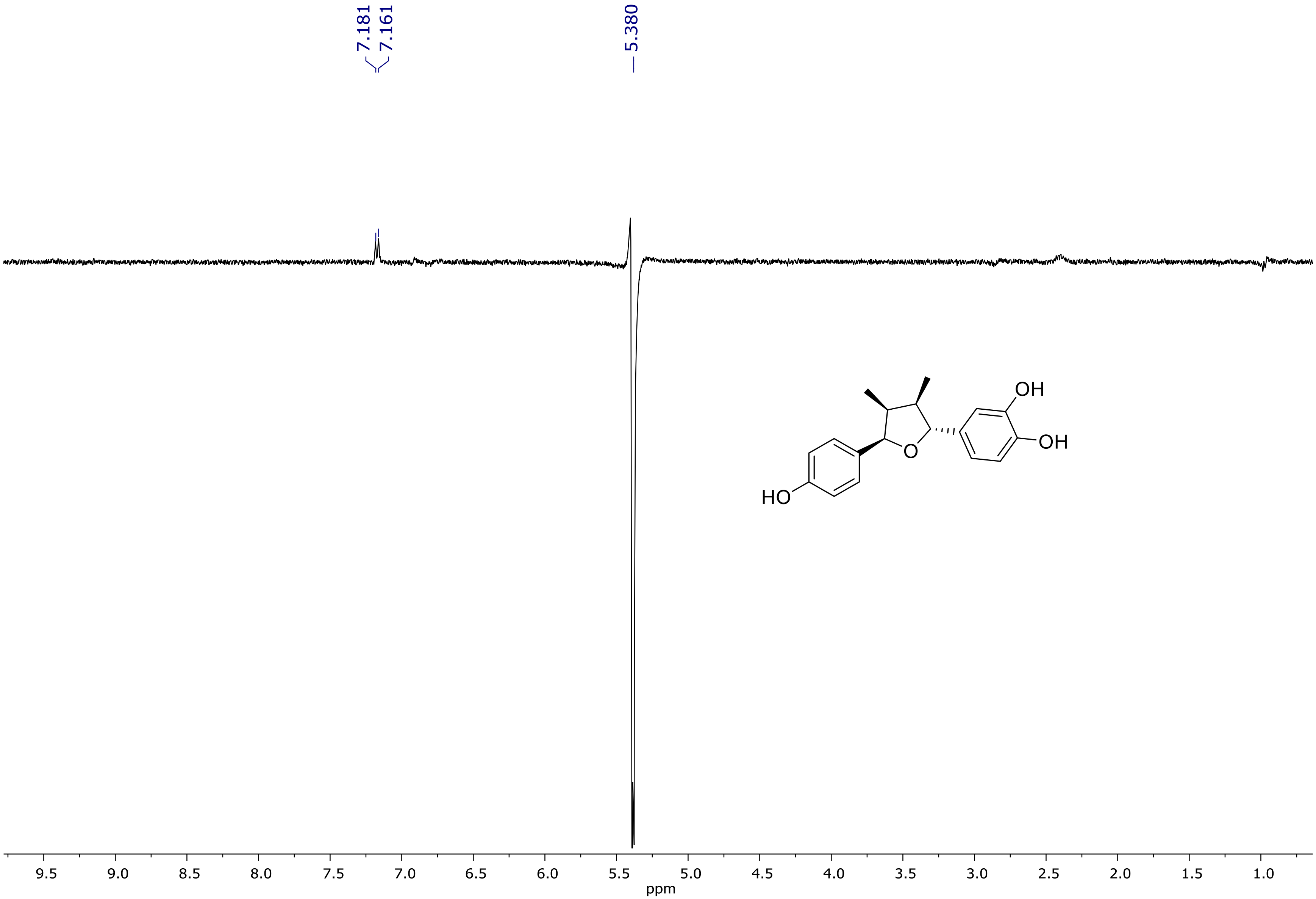

Figure S38. NOESY NMR spectrum of of (7R, 7’R)-7, 7’-bis(4’, 3, 4-trihydroxyphenyl)-(8R, 8’S)-8, 8’-dimethyltetrahydrofuran (**8**) in $(CD_3)_2CO$

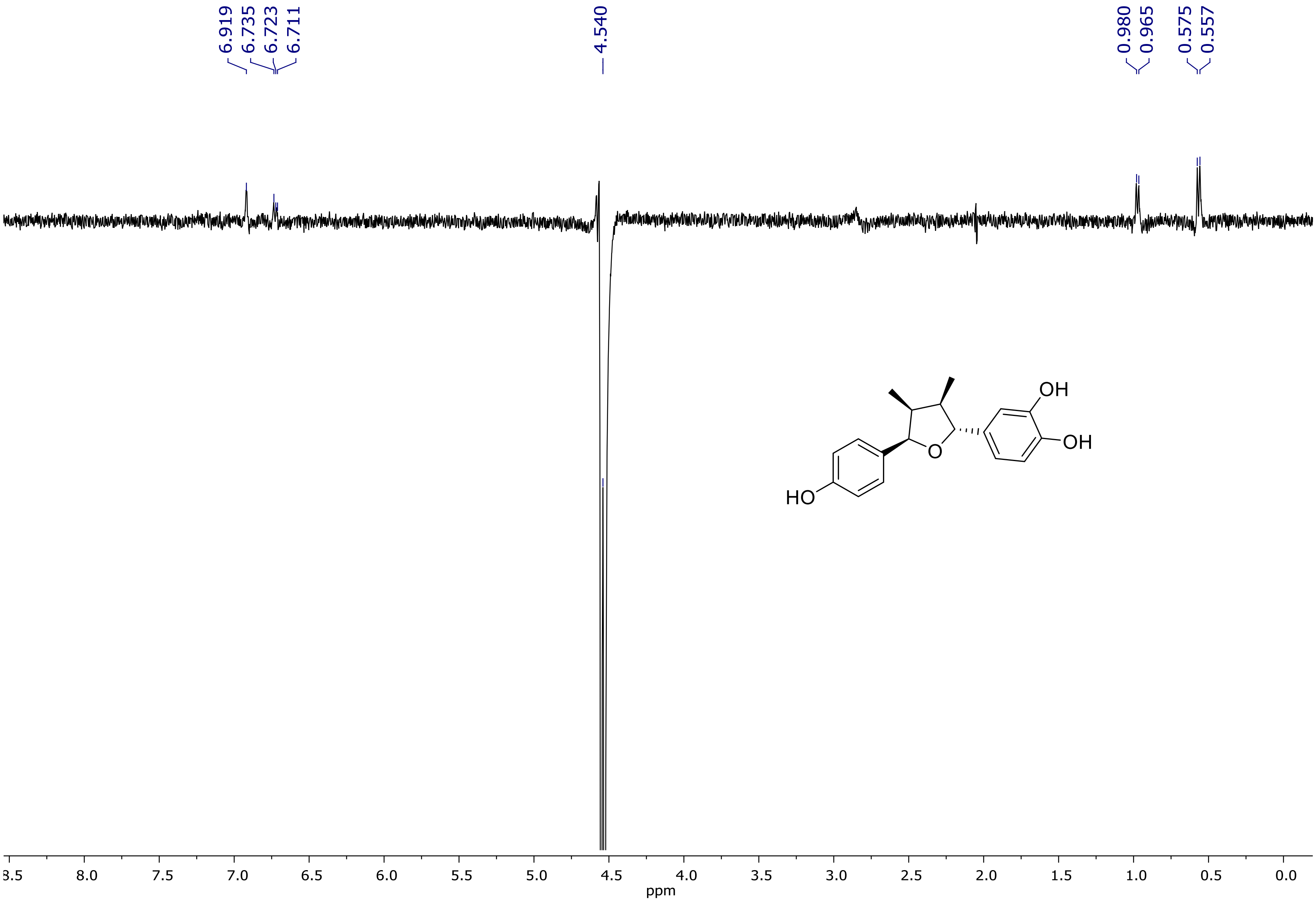

Figure S39. ¹H NMR spectrum of 3-methoxy-6, 7, 4'-trihydroxyflavone (**9**) in $CDCl_3$ and CD_3OD

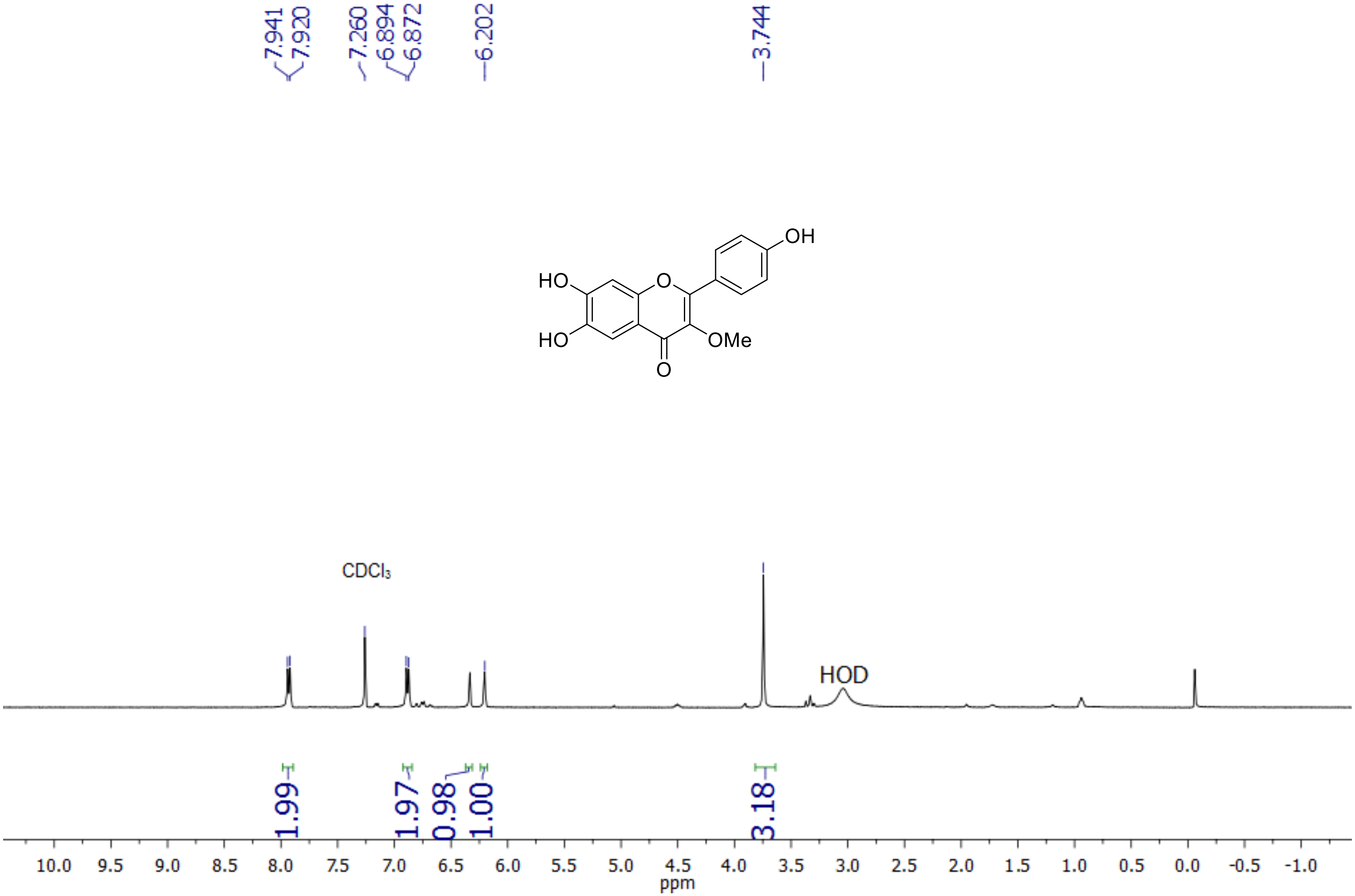

Figure S40. ^{13}C NMR spectrum of 3-methoxy-6, 7, 4'-trihydroxyflavone (**9**) in $CDCl_3$ and CD_3OD

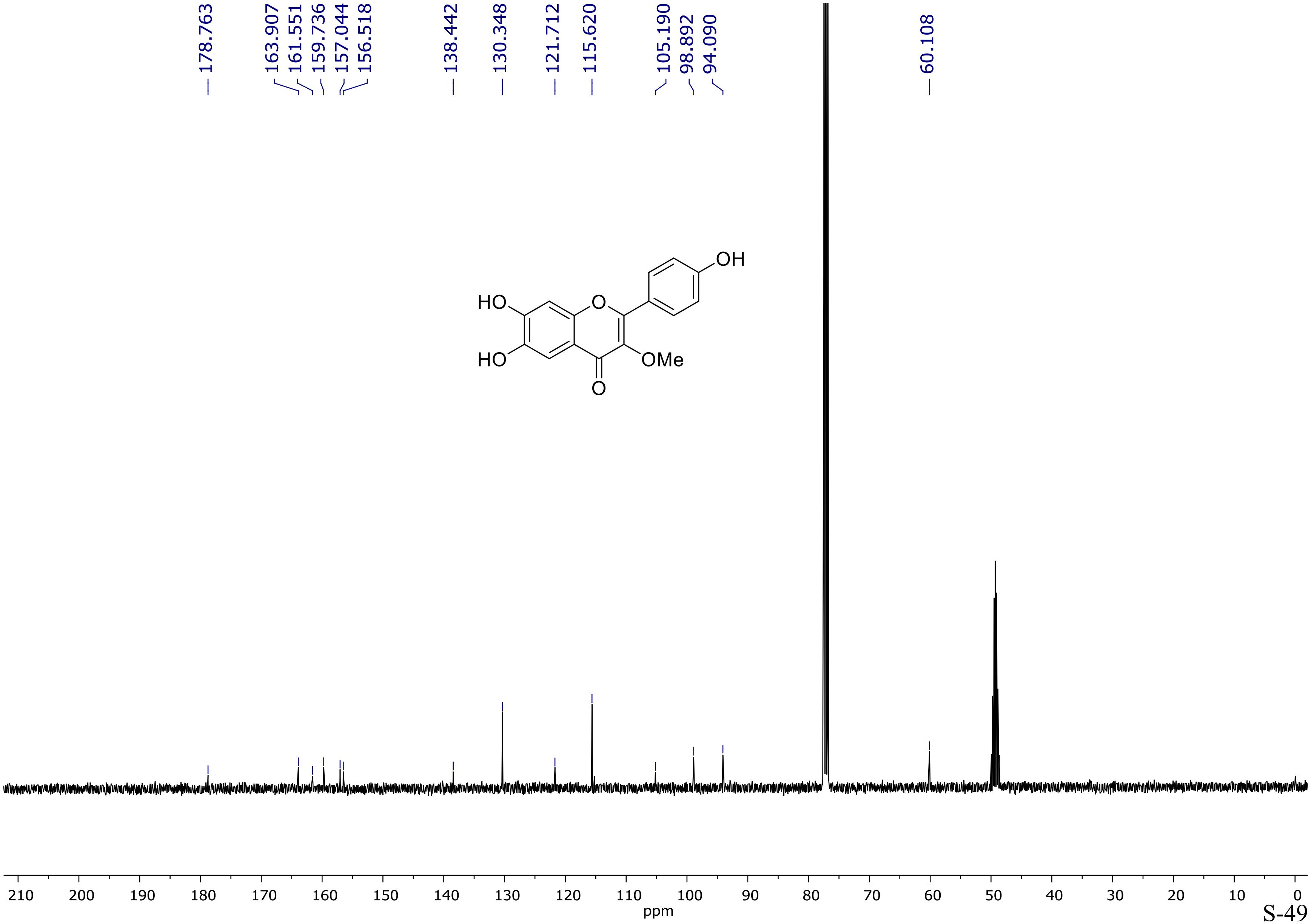

Figure S41. ^{13}C DEPT-135 NMR spectrum of 3-methoxy-6, 7, 4'-trihydroxyflavone (**9**) in $CDCl_3$ and CD_3OD

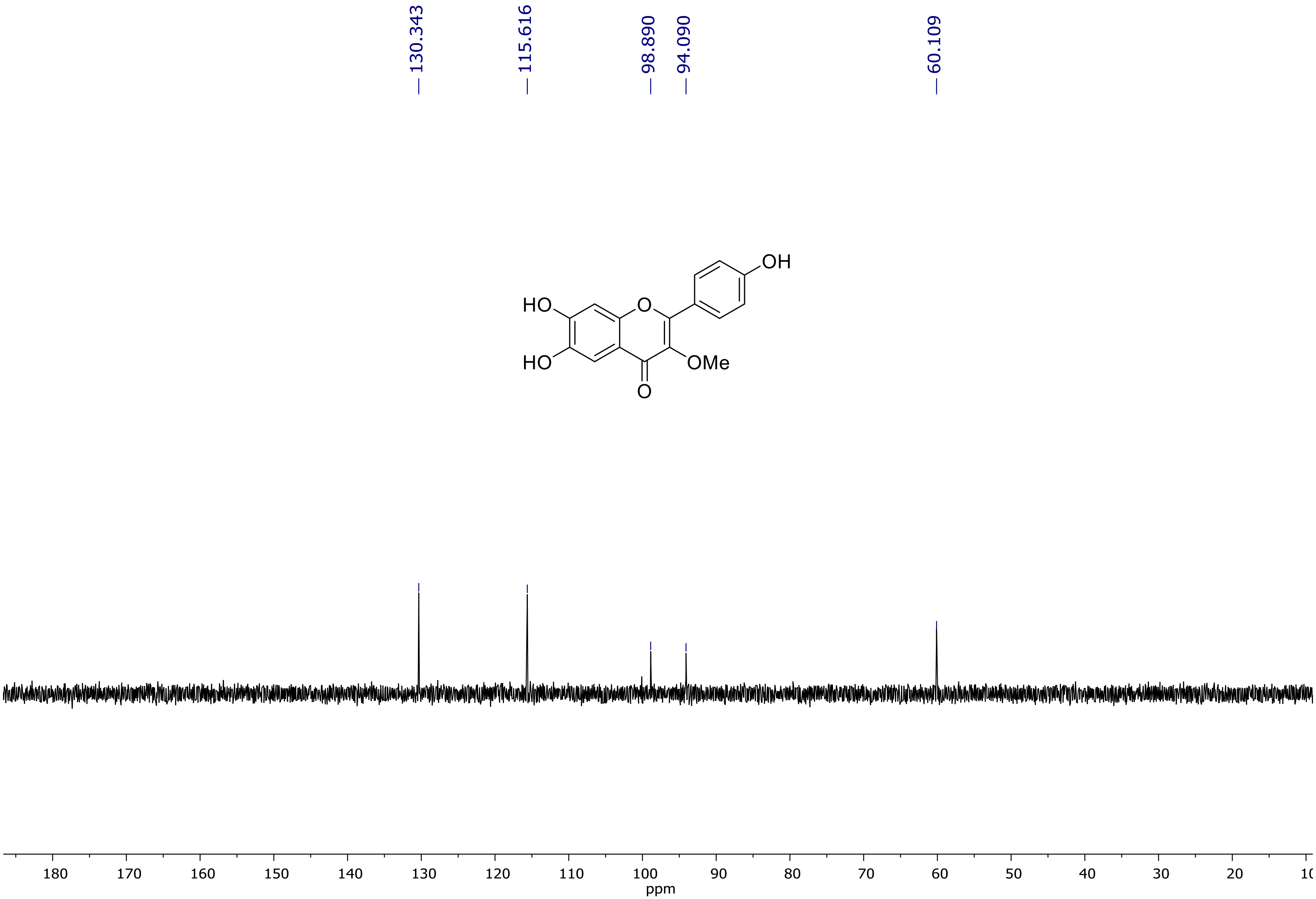

Figure S42. ^{13}C DEPT-90 NMR spectrum of 3-methoxy-6, 7, 4'-trihydroxyflavone (**9**) in $CDCl_3$ and CD_3OD

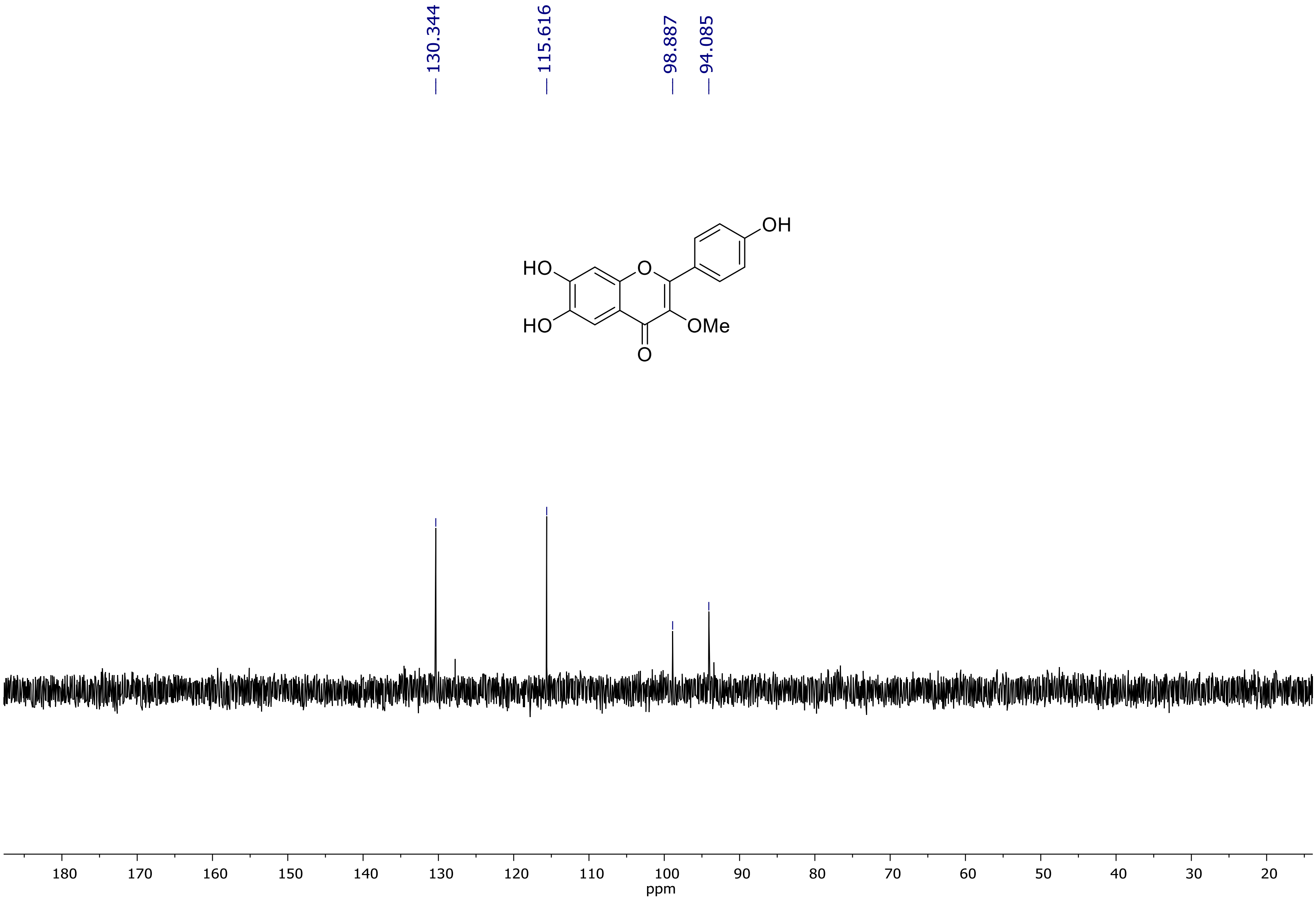

Figure S43. HMQC NMR spectrum of 3-methoxy-6, 7, 4'-trihydroxyflavone (**9**) in $CDCl_3$ and CD_3OD

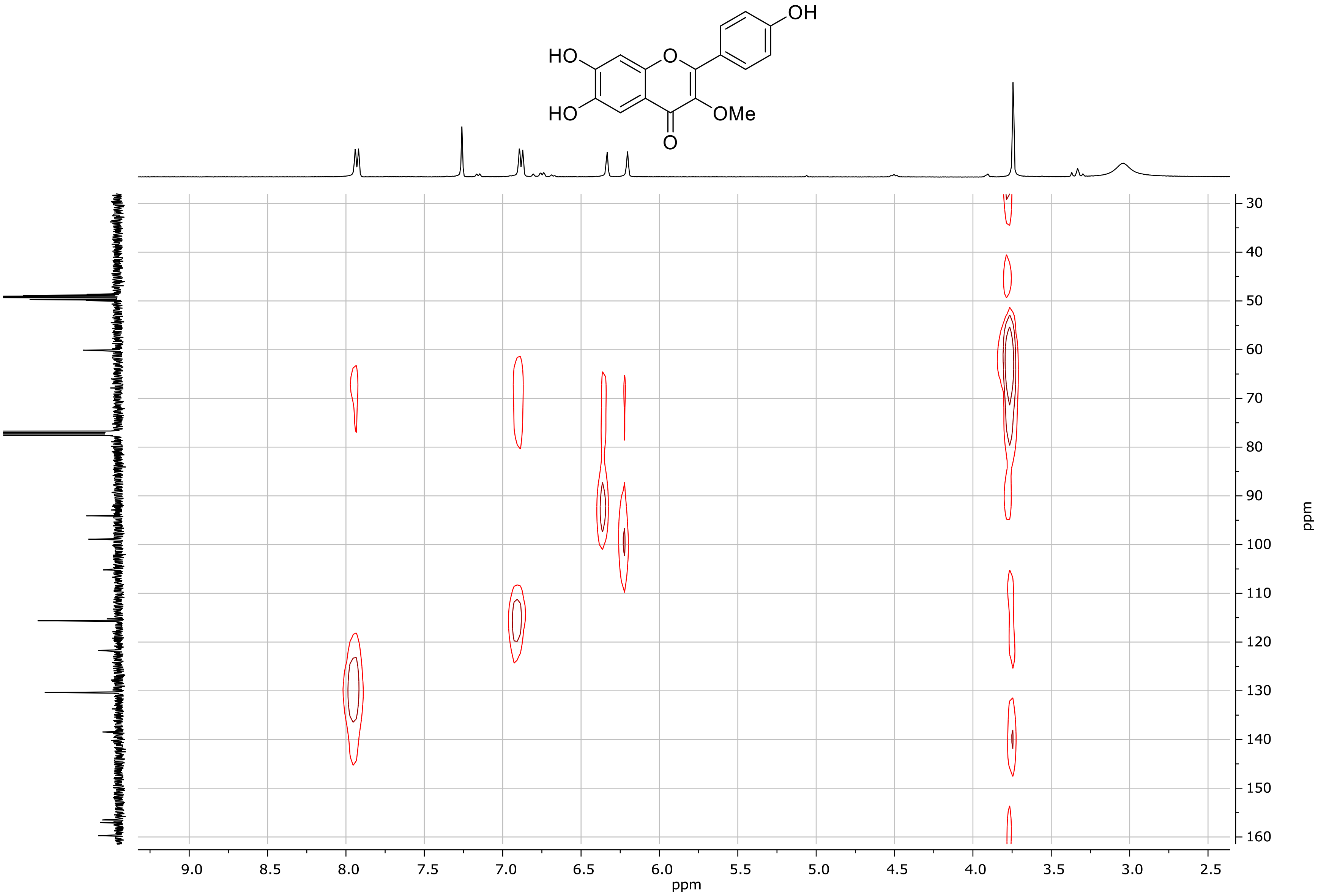

Figure S44. HMBC NMR spectrum of 3-methoxy-6, 7, 4'-trihydroxyflavone (**9**) in $CDCl_3$ and CD_3OD

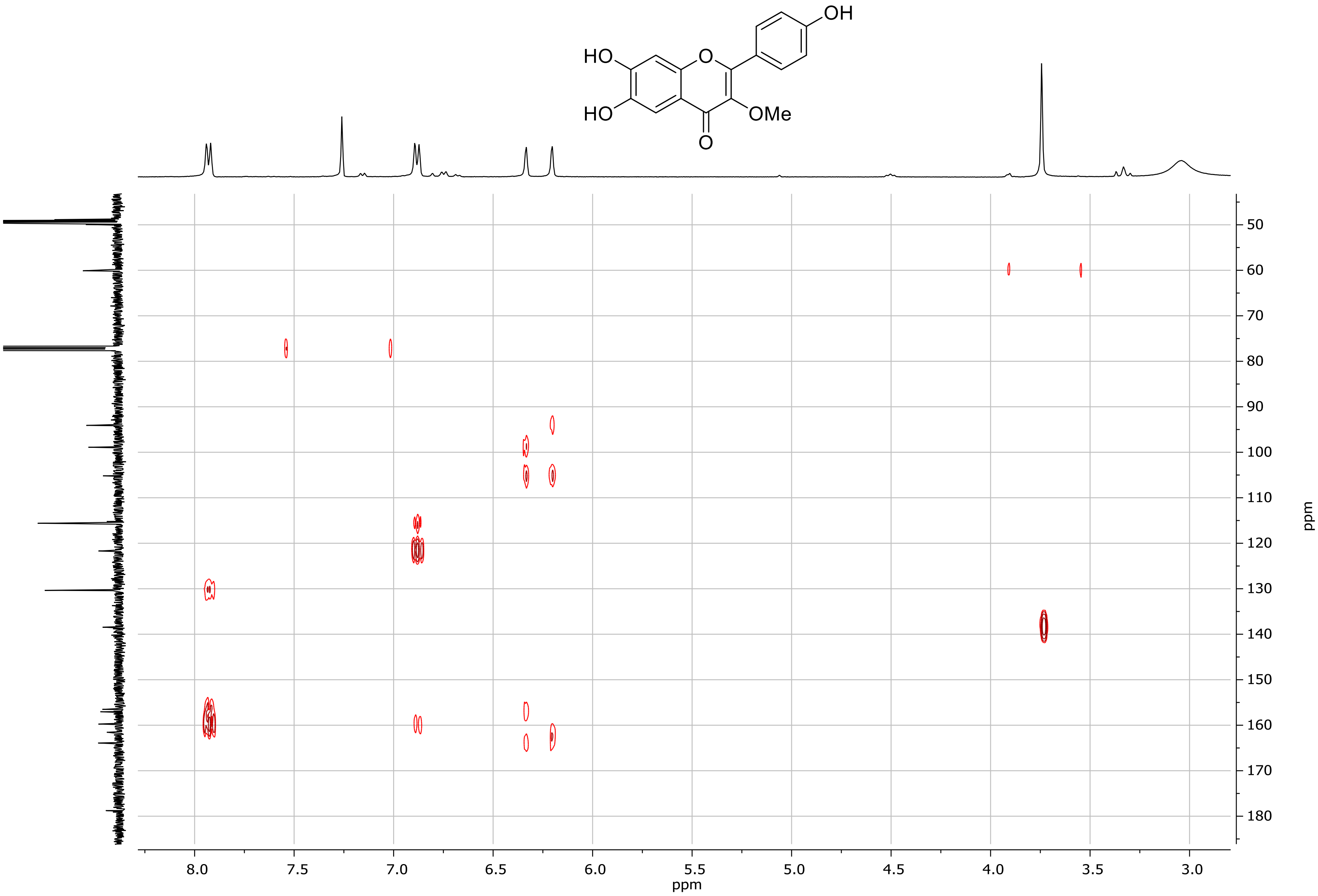

Supplement: S1 Appendix — (PDF) [file pntd.0005832.s001.pdf]
